# The role of invasive alien species in the emergence and spread of zoonoses

**DOI:** 10.1007/s10530-022-02978-1

**Published:** 2022-12-20

**Authors:** Helen E. Roy, Elena Tricarico, Richard Hassall, Charlotte A. Johns, Katy A. Roy, Riccardo Scalera, Kevin G. Smith, Bethan V. Purse

**Affiliations:** 1 UK Centre for Ecology & Hydrology, Benson Lane, Crowmarsh Gifford, Oxfordshire, OX10 8BB UK; 2 University of Florence, via Madonna del Piano 6, 50019 Sesto Fiorentino (FI), Italy; 3 Present Address: Institute of Infection, Veterinary and Ecological Sciences, University of Liverpool, Leahurst Campus, Chester High Road, Neston, CH64 7TE UK; 4 IUCN/SSC Invasive Species Specialist Group, Rome, Italy; 5 International Union for Conservation of Nature (IUCN), David Attenborough Building, Pembroke Street, Cambridge, CB2 3QZ UK

**Keywords:** Zoonotic pathogens, Invasive non-native species, Disease, Spillover, One health

## Abstract

The role of invasive alien species in the transmission dynamics of zoonotic pathogens is often overlooked, despite the rapid escalation in biological invasions globally. Here we synthesise available information on the influence of invasive alien species on zoonotic pathogen dynamics in invaded ranges, focussing on Europe, and identify key associated knowledge gaps. We identified 272 documented interactions between alien species and zoonotic pathogens within invaded ranges. The majority of these involved invasive alien mammals followed by birds with only a few occurrences of other taxa documented. A wide range of potential interactions between invasive alien species and zoonotic pathogens were identified but few studies considered transmission to humans and so there was limited evidence of actual impacts on human health. However, there is an urgent need to raise awareness of the potential risks posed to human health by the transmission of zoonotic diseases by invasive alien species; the role of invasive alien species in zoonotic disease transmission may exceed that of native wildlife and occur in a relatively short period following the arrival of an invasive alien species within a new region. Ecological and social mechanisms govern the dynamics of zoonotic disease transmission but wildlife diseases are not consistently included within animal, plant and human policies. Rapid advances in the development of systems frameworks that integrate the ecological, economic and social processes promoting spillover in rapidly changing environments will increase understanding to inform decision-making.

**Supplementary Information:**

The online version contains supplementary material available at 10.1007/s10530-022-02978-1.

## Introduction

The number of alien species arriving in new regions is escalating globally (Seebens et al. 2017) and the interaction of invasive alien species with land use change could be of similar magnitude to the threat of climate change in shifting the distribution of hosts, vectors and reservoirs of pathogens (Hulme 2014). The adverse effects of invasive alien species 1 on biodiversity and ecosystems have been widely documented (Mazza and Tricarico 2018; Pyšek et al. 2020). However, the role of alien species in the transmission dynamics of emerging zoonotic diseases^2^ is often overlooked (Dunn and Hatcher 2015; Hulme, 2017; Roy et al. 2016).

Zoonotic diseases make up 60% of emerging infectious disease events worldwide (Jones et al. 2008) and disproportionately affect tropical communities (Halliday et al. 2012) accounting for around one-quarter of Disability Life Adjusted Years lost to infectious diseases in Lower Middle Income Countries (Grace et al. 2012). The impacts of these complex, multi-host pathogens are evolving in response to social-political and environmental change including agricultural intensification, deforestation and climate change (Jones et al. 2008; Plowright et al. 2021). An outcome of ongoing social-political and environmental change is the dramatic shifts and expansion of animal hosts (Johnson et al. 2020) which can increase the probability of contact amongst humans, animal hosts and disease vectors with knock-on consequences for exposure and transmission of zoonotic diseases. For example, abundant and widely distributed species, including those that have expanded their ranges by adapting to human-dominated landscapes, were found to harbour higher loads of zoonotic viruses and pose an increased risk of contributing to human spillover worldwide than those with limited distributions (Johnson et al. 2020). Another global analysis highlighted that disturbed habitats, under substantial human use, harbour a greater richness and total abundance of known wildlife hosts of zoonotic diseases than other habitats (Gibb et al. 2020).

Empirical and review studies have highlighted a wide range of mechanisms by which social-ecological and anthropogenic environmental change, including habitat loss, degradation and fragmentation, can alter the interactions between species within a disease transmission network and promote zoonotic disease spillover (Aguirre 2017; Plowright et al. 2021). These have included effects on behaviour, social strucuture and dispersal of species and composition and diversity of communities (Estrada-Peña et al. 2014). Alterations to the composition and diversity of local communities have been shown to increase or decrease zoonotic pathogen transmission. In some cases, new alien species within a community may be suitable hosts for endemic pathogens and so can increase pathogen transmission through “spillback” to native host species including humans. The connectedness between natural and anthropogenic systems highlights the importance of whole systems approaches to understanding the changing dynamics of pathogens in response to global environmental change (Wood et al. 2012).

**Table:** 

**Panel 1: SARS-CoV-2, mink and the potential for spillback of pathogens to humans and other animals** Following an outbreak of SARS-CoV-2 on a farm of American mink, *Neogale vison*, there was strong evidence to suggest the mink had seemingly contracted the infection from spillover from the human pandemic, at least two farm workers have subsequently caught the virus from the mink (Enserink 2020). In December 2020, a wild American mink in Utah near a fur farm was found to be infected with SARS-CoV-2 representing the first case of a non-captive animal infected with this coronavirus. This is of particularly concern, considering that there are studies demonstrating a clear overlap in habitat use between free-ranging mink populations and farm animals (Hammershøj et al. 2005; Valnisty et al. 2020). A further case was reported in Spain with the capture of two American minks in the wild with SARS-CoV-2 infection, although in this case far from fur farms (Aguiló-Gisbert et al. 2021). Further study is required to examine the potential risk to other river-roaming species through indirect transmission routes.	

The extent to which invasive alien species are involved in zoonotic disease transmission in changing environments and through which mechanisms has not been well studied although a recent study demonstrated that the number of zoonosis events increase with the richness of alien zoonotic hosts (Zhang et al 2022).

It has been hypothesised that invasive alien species may have a disproportionate impact on the transmission of zoonotic pathogens for a number of reasons (Hulme 2014):

- Alien species may be more effective hosts than other species or vectors in the transmission of endemic pathogens and amplify local pathogens (Chinchio et al. 2020)
- Alien species may facilitate the establishment of new emerging diseases with which they have co-evolved with in their native range and which may be introduced with them (Dunn and Hatcher 2015)
- Alien species often thrive in anthropogenic environments so may have high encounter rates with people and often exhibit high dispersal rates including through human-mediated dispersal, with the trade in many alien species being relatively unregulated.
- The integration of a new host into an established zoonotic network can dramatically increase disease transmission.

Here we present a review of the literature on the role of alien species in the emergence and spread of zoonoses recognising the mounting evidence of links between zoonotic diseases and biodiversity change (Johnson et al. 2020; Nuñez et al. 2020). A recent analysis highlighted that the introduction of alien species is likely to have contributed to zoonosis emergences in recent history (Zhang et al 2022). Here we assess the extent to which the impacts of invasive alien species on zoonotic disease transmission have already been realised and assessed possible biases in the information available and opportunities for improving our scientific understanding of the role invasive alien species play in zoonotic disease transmission to inform interventions and policy.

## Methods

We conducted a systematic review of published literature following PRISMA guidelines using the Web of Knowledge platform, which includes references from 1960 onwards. All databases within Web of Knowledge were searched, which included Web of Science Core Collection, BIOSIS Citation Index, BIOSIS Previews, KCI Korean Journal Database, MEDLINE, Russian Science Citation Index, SciELO Citation Index (search date: 12th July 2020). We utilised six sets of search strings to retrieve literature (Table 1), which yielded 603 unique references once duplicates were removed. No special operators were used which effectively performs an AND operation between the words in the set. Our search only included papers written in the English language although it should be noted that literature in other languages would undoubtedly have add to the information available.

**Table 1:** Sets of search strings used to retrieve literature linking zoonotic diseases and invasive alien species and the number of references returned for each set

Sets of search terms	Number of references returned
Alien species zoonoses	48
Alien species zoonotic diseases	50
Invasive species zoonoses	262
Invasive species zoonotic diseases	272
Non-native species zoonoses	312
Non-native species zoonotic diseases	290

For extraction of data into summary tables, we agreed on inclusion and exclusion criteria which aligned with the scope of the review (Table 2). Full papers were identified following screening of all titles and abstracts, by one of three of the study authors (BVP, ET, HER), and further reviewed as necessary for eligibility and inclusion. Overall, 369 papers out of the 603 were excluded from the study.

**Table 2:** Inclusion and exclusion criteria used to select studies for the review

Inclusion Criteria
Contains primary data on populations of alien species established in the wild outside their native range and causing (or having potential to cause) zoonotic disease
Contains primary data on the role of alien species (or potential role) as a vector or reservoir species for a zoonotic disease in the wild outside their native range
Reviews the role of alien species in zoonotic disease transmission and spread in the wild outside their native range (either considering only the alien species or the alien species in comparison to native species)
Contains primary laboratory data on competence of alien species which are vectors or reservoirs for a zoonotic pathogen where the laboratory test populations arefrom outside the native range

Exclusion Criteria
Contains only ecological, taxonomic, genetic or physiological data on the alien species with no data on a zoonotic disease
Contains data on alien species involved as a vector or reservoir species for a zoonotic disease in the native range of the IAS only and with no information from outside the native range
Contains data on invasiveness of a pathogen inside host tissue as opposed to data on an alien species
Contains data on a non-invasive scientific method as opposed to data on alien species
Contains data on zoonotic disease in humans without linkage to ¹alien species
Contains data on zoonotic disease links to alien species hosts but the hosts are not identified to species
Contains data on alien species links to zoonotic disease where alien species populations are captive or kept as pets only with no free living alien species populations
Contains data on bites by an alien species as health problem rather than infectious zoonotic disease
Reviews that do not explicitly link alien species and zoonotic diseases (e.g. of invasion and biosecurity policy, zoonotic diseases and ecosystems, zoonotic diseases and biogeography, wildlife trade)

### Data synthesis and summary measures

Data on invasive alien species-zoonotic pathogen interactions from relevant papers was extracted into summary tables (Supplementary Information 1) including taxonomic information (order, family, species) of the invasive alien species and the species or genus name of the pathogen. The role of the invasive alien species in zoonotic disease transmission was classified within broad categories of direct and indirect roles. Direct roles in transmission included being an invasive alien species pathogen of humans, being a reservoir host for a zoonotic pathogen, being an arthropod vector for the zoonotic pathogen (biological or mechanical). Indirect roles included being a host for arthropod vectors of zoonotic pathogens, being a vector for a zoonotic pathogen, or altering vector-host–pathogen dynamics in ways that increase transmission to humans.

The type of study was also categorised for each paper. Some of the studies were laboratory-based such as those considering the competence of vectors or reservoirs within the context of zoonoses and others on molecular phylogenetics. Many of the studies were field-based including screening approaches to assess pathogen prevalence within an invasive alien species host through to ecological studies investigating the mechanisms behind zoonotic transmission. There were also a number of biogeographic studies and review papers identified. For each study and where available the following contextual factors were collected: the region and country in which the study took place, the timing of introduction of the alien species and current extent of establishment and spread in the country, information on the status of the zoonotic disease in the study region.

To clarify and quantify the extent of impact that invasive alien species are involved in transmission of the parasite or pathogen to humans, we extracted further information on the role of the invasive alien species in the pathways to zoonotic disease transmission (Plowright et al. 2017, 2021) from all the papers identified as relevant (conforming to the inclusion criteria outlined in Table 2).

The evidence for each individual invasive alien species and zoonotic pathogen interaction was revisited to evaluate the extent of impact that invasive alien species are having on transmission from a potential impact to an actual (= realised) impact along a continuum (identified by the authors) of available supporting evidence (Table 3). This provided an opportunity to give context to the interaction and specifically some measure of confidence in the extent and magnitude of the impact on humans. Pathogen type and transmission pathways were retrospectively retrieved for pathogens involved in the interactions using a wide range of literature sources (Supplementary Information 2).

**Table 3:** Continuum of potential and actual impacts of IAS on zoonotic disease spillover with types of supporting evidence

Type of impact	Certainty of impact	Type of supporting evidence
Potential	Very low	Detection of sporadic pathogen presence or low prevalence (< 5%) in populations of IAS outside their native range
Potential	Low	Detection of medium pathogen prevalence in populations of IAS (5–20%) outside their native range, especially where less than native species
Potential	Medium	High pathogen prevalence in populations of IAS outside their native range, especially where the same or higher than native species
Potential	High	High pathogen prevalence in populations of IAS outside their native range, especially where higher than native species, widespread and abundant in anthropogenic habitats, laboratory/dissection studies supporting role in transmission
Actual	Low	IAS shown to play a role in transmission to people but low case number or prevalence outside their native range
Actual	Medium	IAS shown to overlap spatially or temporally with the distribution of human outbreaks outside the native range of the IAS
Actual	High	IAS shown to have changed the distribution or spread of autochthonous transmission or human outbreaks outside the native range of the IAS
Actual	High	IAS shown to alter strain diversity and population structure of pathogens and strains shared with humans outside the native range of the IAS

Additionally, further information is provided on type of transmission and broad host associations and human health impact specifically in Europe noting this study was funded by the European Commission (Supplementary Information 2). Transmission was coded as: A = aerosol transmission, C = contact (skin and mucosal) transmission, O = oral transmission through food (F) or water (W) or vector-borne transmission (V) by either flea (F), tick (T), mite (MI), lice (LI), biting flies (BF), Triatminae i.e. kissing bugs (Tri) or mosquitoes (MOS). The annual cases and case fatality rates are also reported within the tables and all information is taken from European Centre for Disease Prevention and Control (2012–2018) where data are available

**Table 4:** Broad group of pathogen or parasite (V = Virus, B = Bacteria, F = Fungus, Pr = Protozoa, N = Nematode, C = Cestode, T = Trematode, Pl = Platyhelminth) associated with invasive alien species including the number of genera and species (where information was available) and the invaded countries in which the association has been observed. Further notes on taxonomic groups including detailed species-specific examples are provided within the Supplementary Information

Invasive alien species	Common name	V	B	F	Pr	N	C	T	Pl	Number of parasite/pathogen genera	Number of parasite/pathogen species	Countries
**Arthropoda: Arachnida**												
*Rhipicephalus microplus*	Asian blue tick									1	1	Guam
*Hyalomma marginatum*	Mediterranean Hyalomma		✓							1	1	Austria
*Rhipicephalus appendiculatus*	Brown ear tick				✓					1	1	Comoros Islands
*Rhipicephalus sanguineus*	Kennel tick		✓							1	1	Switzerland
**Arthropoda: Insecta**												
*Aedes aegypti*	Yellowfever mosquito	✓				✓				2	2	Argentina Switzerland USA
*Culex pipiens*	Common house mosquito					✓				1	2	Argentina
*Aedes japonicus*	Asian bush mosquito					✓				1	2	Switzerland
*Aedes albopictus*	Tiger mosquito					✓				1	2	Italy
*Aedes japonicus japonicus*	Asian bush mosquito	✓								5	0	USA Switzerland
*Culex quinquefasciatus*	Southern house mosquito	✓								1	0	USA
**Maxillopoda**												
*Austrominius (Elminius) modestus*	Acorn barnacle	✓								1	0	UK
**Gastropoda**												
*Biomphalaria glabrata*	Bloodfluke planorb							✓		1	1	Romania
*Biomphaliaria tenagophila*								✓		1	1	Romania
*Achatina fulica*	Giant African land snail					✓				1	2	USA
*Melanoides tuberculata*	Red-rimmed melania								✓	5	6	Costa Rica Peru Brazil Venezuela
*Alcadia striata*	Straite drop					✓				1	1	USA
*Bradybaena similaris*	Asian trampsnail					✓				1	1	USA
*Zachrysia provisoria*	Cuban brown snail					✓				1	1	USA
**Actinopterygii**												
*Oreochromis niloticus*	Nile tilapia							✓		2	0	China
*Cyprinus carpio*	European carp							✓		2	0	China
*Oncorhynchus keta*	Chum salmon					✓				1	1	Europe
**Aves**												
*Sturnus vulgaris*	Common starling		✓							4	2	USA New Zealand
*Branta canadensis*	Canada goose		✓							2	1	Belgium UK
*Alectoris chukar*	Chukar partridge		✓							1	1	USA
*Myiopsitta monachus*	Monk parakeet				✓					1	0	Chile
*Psittacula krameri*	Rose-ringed parakeet	✓	✓							1	1	France Japan
*Columba livia*	Common pigeon	✓								2	0	USA
*Streptopelia chinensis*	Spotted dove	✓								2	0	USA
*Padda oryzivora*	Java sparrow	✓								2	0	USA
*Fringilla coelebs*	Common chaffinch				✓					1	0	New Zealand
*Turdus philomelos*	Song thrush	✓			✓					2	0	USA New Zealand
*Turdus merula*	Common blackbird				✓					1	0	New Zealand
*Prunella modularis*	Dunnock				✓					1	0	New Zealand
*Passer domesticus*	House sparrow	✓			✓					3	1	USA New Zealand
**Mammalia**												
*Sus scrofa*	Wild boar	✓	✓		✓					18	24	USA Australia Georgia Brazil Chile
*Nyctereutes procyonoides*	Raccoon dog	✓	✓			✓	✓	✓		13	9	Germany Poland Estonia The Netherlands Denmark Lithuania Austria Latvia
*Canis lupus*	Grey wolf						✓			1	1	Estonia
*Canis lupus familiaris*	Domestic dog		✓							1	3	Estonia
*Canis lupus dingo*	Dingo		✓							1	1	Australia
*Vulpes vulpes*	Fox		✓				✓			3	4	Australia
*Felis catus*	Domestic cat		✓		✓	✓				10	9	Australia USA Denmark
*Neogale vison*	American mink	✓	✓		✓	✓	✓			8	3	Spain Chile Poland
*Procyon lotor*	Northen raccoon	✓	✓			✓				19	10	Japan Denmark Germany Poland Norway China Austria
*Ondatra zibethicus*	Muskrat	✓	✓		✓		✓			7	6	Germany Poland France
*Myocastor coypus*	Coypu		✓		✓	✓	✓			5	7	Korea France Japan Italy
*Mus musculus*	House mouse	✓	✓		✓	✓				10	3	New Zealand Senegal Australia Argentina Chile Puerto Rico USA Nigeria Madagascar
*Tenrec ecaudatus*	Common tenrec		✓							1	1	Mayotte
*Herpestes javanicus*	Javan mongoose	✓								1	0	Japan
*Mustela putorius furo*	Ferret				✓					1	0	New Zealand
*Nasua nasua*	Ring-tailed coati				✓					1	0	Norway
*Paguma larvata*	Masked palm civet		✓							3	1	Japan
*Didelphis marsupialis*	Common opossum		✓							1	1	USA
*Trichosurus vulpecula*	Common brushtail possum				✓					2	0	New Zealand
*Erinaceus europaeus*	European hedgehog				✓					1	0	New Zealand
*Lepus europaeus*	European hare							✓		1	1	Chile Argentina
*Oryctolagus cuniculus*	European rabbit				✓					1	0	New Zealand
*Chlorocebus aethiops sabaeus*	African green monkey		✓							1	1	St. Kitts
*Macaca mulatta*	Rhesus macaque	✓								2	0	Puerto Rico USA
*Chinchilla lanigera*	Long-tailed chinchilla						✓			1	1	Switzerland
*Gerbillus nigeriae*	Nigerian gerbil		✓							1	1	Senegal
*Herpestes auropunctatus*	Indian mongoose		✓							1	0	Puerto Rico St. Kitts
*Suncus murinus*	Asian house shrew		✓							1	0	Madagascar
*Callosciurus finlaysonii*	Finlayson's squirrel	✓								1	0	Germany
*Sciurus carolinensis*	Grey squirrel	✓								1	0	UK Italy Germany
*Sciurus variagata*	Variegated squirrel	✓								1	0	Germany
*Tamias sibiricus barberi*	Siberian chipmunk		✓							1	1	France
*Tamiops swinhoei*	Swinhoe's striped squirrel	✓								1	0	Germany
*Rattus norvegicus*	Brown rat		✓			✓	✓			40	42	USA South Africa Grenada Australia Madagascar Puerto Rico Canada Argentina Chile Nigeria
*Rattus rattus*	Black rat	✓	✓		✓	✓	✓			44	23	Australia USA Argentina South Africa Italy Chile Uganda Madagascar Senegal Diego Garcia New Zealand La Réunion Mauritius Seychelles Swaziland Mozambique Puerto Rico Malayasia Borneo Brazil Benin Nigeria
*Rattus tanezumi*	Asian house rat					✓				4	3	South Africa

## Results

Overall 272 direct interactions between invasive alien species and zoonotic pathogens in the invaded range (Supplementary Information 1), conforming to our inclusion criteria, were identified from the literature review including other recent review papers (Hulme 2014; Zhu et al. 2019). Only five species (a reptile, three plants, and a plant pathogen) were identified as having an indirect impact on zoonotic pathogen transmission, by altering host-vector pathogen dynamics (Table 4; Supplementary Information 1). The vast majority of direct interactions identified involved invasive alien vertebrates as potential or actual reservoir species for zoonotic pathogens, with 213 (78.3%) invasive alien species-pathogen genus interactions identified for mammals, 26 (9.6%) for birds, 3 (1.1%) for non-avian reptiles and 4 (1.5%) for fish. For invertebrates, 14 (5.1%) invasive alien species-pathogen interactions involved invasive ticks (4) or insects (10) as biological vectors, whilst one (0.4%) involved an invasive alien crustacean and 11 (4.0%) involved invasive alien molluscs as intermediate reservoir hosts for zoonotic pathogens. Six nematodes and five platyhelminthes were identified as zoonotic endo-parasites that had been recently introduced to Europe, with some degree of impact on human health.

**Table:** 

**Panel 2. Examples of evidence of actual impacts on human health following transmission of a zoonotic disease by an invasive alien species** There is unequivocal evidence that the spread and maintenance of serious human pathogens such as bacteria from the genera *Leptospira*, *Bartonella* and *Yersinia* followed the widespread human-mediated introduction of Norwegian rat, *Rattus norvegicus*, and the black rat, *Rattus rattus* to new areas, especially islands, through shipping during the sixteenth and seventeenth centuries. The evidence for the actual impacts on transmission was based on global biogeographical studies of pathogens detected from rats and humans, facilitated by advances in molecular methods (Kosoy and Bai 2019) alongside empirical studies on host-vector-pathogen interactions (Moseley et al*.* 2018). Other significant actual impacts come from more recent invaders to Europe, such as *Aedes* mosquito vectors including *Ae. albopictus* providing the conditions for autochthonous outbreaks of arboviruses such as Dengue and Chikungunya in Europe within a decade of initial invasion and establishment (Leta et al. 2018).	

### Evidence of an actual rather than a potential impact of the invasive alien species on the zoonotic disease transmission

Evidence of an actual rather than a potential impact of the invasive alien species on the zoonotic disease transmission and human health was only demonstrated within a few studies despite a wide range of interactions between invasive alien species and zoonotic diseases being identified (Supplementary Information 1). The following groups of pathogens were implicated (actual or potential) with alien or invasive alien vectors or hosts in transmission: 52 bacteria, 23 viruses, 33 other endoparasites and these combined with the invasive alien species to give 204 zoonotic pathogen – alien or invasive alien species interactions. Of these 22 were aerosol-transmitted, 33 were contact-transmitted, 119 orally-transmitted, 22 were water-borne, 55 were food-borne and 55 were vector-borne (details in Supplementary Information 2).

### Evidence for invasive alien species playing an equivalent or disproportionate role in zoonotic disease spillover

Many of the studies reviewed compared host–pathogen interactions between invasive alien and native host species, usually for a single pathogen at a time, but sometimes for multiple native hosts and multiple pathogen species. Of the 65 host–pathogen interactions identified from these comparative studies, 57 focused on mammalian hosts including 13 host species with the most numerous being raccoon dog, *N. procyonoides* (32%; n = 21), black rat, *R. rattus* (26%; n = 17), Norway rat, *R. norvegicus* (11%; n = 7), house mouse, *M. musculus* (5%; n = 3). The remaining eight invasive alien species were birds (8%; n = 5) and insects (5%; n = 3). Across all the comparative studies, the pathogen prevalence (derived from assessing pathogen presence using sero-prevalence or PCR-positive detections within host animals surveyed in a variety of ways) across sampled invasive alien species host individuals was lower than in the native host in 15 studies, equivalent to the native host in 14 studies, and higher than the native host in 33 studies.

## Discussion

Invasive alien species are involved in the transmission of zoonotic pathogens in a range of endemic and epidemic disease situations and geographical contexts, although noting many studies identified potential rather than actual impacts on transmission, with a wide range of ecological, environmental and social processes modulating their role in transmission.

Concerning whether invasive alien species play a stronger role in transmission than native hosts, comparisons of infection prevalence between such sympatric hosts was only widely studied in mammal species. For this taxon, pathogen prevalence was higher in the invasive alien host than the native host in around half of the studies, indicating that invasive alien populations can be more widely infected than sympatric native host populations by zoonotic pathogens. Whether this translates into a stronger role of invasive alien species in transmission and spillover of zoonotic diseases (Hulme 2014) depends on a wide range of other physiological, behavioural factors and evolutionary (including habitat and resource use, overlap with people affecting rates of contact, rate and conditions favouring pathogen susceptibility and shedding by hosts; Murray and Daszak 2013; Hartemink et al. 2015; Plowright et al. 2017). These factors will need to be unravelled through detailed empirical ecological studies of interactions between sympatric native and invasive alien hosts, people and pathogens; such studies are currently rare.

Species ecological traits determine the role that invasive alien species play in transmission relative to native species. As an example, raccoon dogs, *N. procyonoides*, were assessed in a number of studies as a reservoir host for a range of intestinal pathogens, the bacterial pathogen *Francisella tularensis* and rabies. A number of the traits that have contributed to the invasion success of raccoon dogs were also considered to be important in altering the spatial and temporal dynamics of the tapeworm *Echinococcus multilocularis*; raccoon dogs can colonise wide areas over a short time and have a high reproductive rate. They are considered to be an important definitive host for the tapeworm. The disease, alveolar echinococcosis, caused by the larval form of this tapeworm, is a highly lethal helminthic disease in humans; the prevalence of the disease is increasing in Europe (Torgerson et al. 2015), particularly in western Europe, and has been attributed to the abundance of foxes and meadow voles. Studies from Poland, Germany and Estonia all demonstrated a lower prevalence of the tapeworm in raccoon dogs compared to the native red foxes sampled in the same period. It is thought that this might be a consequence of diet. Red foxes consumed more arvicoline rodents, the main intermediate hosts of the tapeworm, particularly during the coldest period of the year when raccoon dogs are in hibernation (Laurimaa et al. 2015). However, raccoon dogs can reach very high densities and so are potentially an important additional definitive host for *E. multilocularis*.

The roles of a wide diversity of native hosts and their pathogens and parasites, including livestock, should be considered when assessing the evidence for the importance of invasive alien species (and other alien species) in disease transmission (Mazza and Tricarico 2018). The majority of emerging human diseases are known to originate from mammals which may be attributed to their vast global distributions and diversity (Han et al. 2016). Moreover particular mammalian taxa, such as bats and rodents, with traits that foster adaptation to anthropogenic environments and to transmission roles, have been identified as being particularly important in spillover (Han et al. 2016; Johnson et al. 2020). A number of mammalian invasive alien species have been very well-studied and dominate amongst vertebrate invasive alien species in terms of numbers and diversity of known potential interactions with zoonoses. Such mammalian invasive alien species are often widely distributed beyond the native range and have close association with humans because they thrive in anthropogenic habitats (rats, *Rattus* spp*.,* mice, *Mus* spp*.,* raccoon dog, *N. procyonoides,* raccoon, *P. lotor*) or are managed by humans in one way or another (wild boar, *Sus scrofa,* feral American mink, *N. vison*). Amongst the invasive alien species that are commonly associated with human-dominated landscapes, mammals have been the focus of most of the studies with reptiles and birds being less well-studied.

Rodents are known to be the most species rich of zoonotic mammalian hosts and to harbour high diversities of zoonotic pathogens, alongside bats, compared to other mammalian taxa (Johnson et al. 2020); 17 invasive alien rodents were included in the interactions with zoonotic pathogens that were identified through this literature review. Rodent reservoir hosts are characterised by high reproductive potential (reproducing early in life and with high frequency) which favours pathogen transmission and maintenance within reservoir populations (Han et al. 2015). Furthermore, many of the rodents documented as invasive alien species favour urban habitats and as such live in close proximity with humans increasing the probability of human exposure to the zoonotic pathogens they carry. Norway rats (*R. norvegicus*) and black rats (*R. rattus*) are considered to be amongst the most damaging invasive alien species globally (Feng and Himsworth 2014) and inhabit every continent except for Antarctica. Their role in transmitting a number of zoonotic pathogens has been well documented (Pépin et al. 2016). Leptospira and hantaviruses, particularly Seoul virus, are the most important in terms of human morbidity and mortality (Pépin et al. 2016). It is intriguing to note the vast variability in prevalence of pathogens borne by rats, even within limited geographical distances with interacting host populations (Pépin et al. 2016) which has implications for surveillance and monitoring. Furthermore, molecular studies are expanding the known diversity of pathogens carried by rats, for many of which the human health implications are unknown, further demonstrating the dynamic nature of the role of invasive alien species in zoonoses transmission and epidemiology.

Consistent with studies of native species-zoonotic pathogen interactions, land use practices and changes were found to modulate a number of the invasive alien host–pathogen interactions. In Chile *Leptospira* species in rodent communities inhabiting agricultural areas were almost three times more infected than in wild areas (Correa et al. 2017) and the invasive Norwegian rats were the most infected species (38.1%). Similarly, the association between the black rat and sewers resulting in high population densities of this invasive alien species was seen to be a contributing factor to the increased prevalence of *Leptospira* in the western Indian Ocean islands and neighbouring Africa (Dietrich et al. 2018). Urbanisation has led to conditions that favour highly adaptable species such as mice and rats and in a study on the cities of Southern Benin the infection rates of black rats, *R. rattus*, and Norwegian rats, *R. norvegicus*, with *Trypanosoma lewisi* was higher than in native rodents (Dobigny et al. 2019).

Human management of livestock and wildlife populations can dramatically influence the extent to which invasive alien species play a role in zoonotic disease transmission. Wild boars, *Sus scrofa*, are reservoirs for many pathogens transmissible to humans such as foodborne zoonoses including bacterial diseases (brucellosis, salmonellosis, tuberculosis and yersiniosis), parasitic diseases (toxoplasmosis and trichinellosis) and the viral 19 hepatitis E. Supplemental feeding to increase the density of wild boar for hunting can increase transmission risk and hunters are at highest risk. Pathogen prevalence also varies with habitat; incidence of *Brucella suis* and *Escherichia coli* were highest in boar from forested and agricultural regions respectively (Lama and Bachoon 2018). Furthermore, hunting with dogs compared to other management practices can increase the risk of pathogen transmission from wild boar to humans (Carr et al. 2019). It is postulated that hunting with dogs may elevate stress and birth rates, leading to high rates of pathogen excretion, but may also alter animal movements and social structure in ways that increase contact rates and pathogen transmission (Carr et al. 2019). The complexity of disease transmission and the interplay of ecological and social factors has implications for the outcomes of invasive alien species management (eradication and population reduction).

Four of the studies we reviewed addressed indirect impacts of invasive alien species on disease transmission. Invasive alien species can alter the habitat use by native species and indirectly increase the human risk of exposure to a disease (Allan et al. 2010). In Missouri (U.S.) the white-tailed deer, *Odocoileus virginianus*, the dominant host for the tick *Amblyomma americanum* carrying the bacteria *Ehrlichia* spp. (agents of human ehrlichiosis), used areas invaded by the Amur honeysuckle, *Lonicera maackii,* more frequently. This led to considerably greater numbers of ticks infected with pathogens in honeysuckle-invaded areas than the adjacent honeysuckle-uninvaded areas. When honeysuckle was experimentally removed, a decrease in deer activity and infected tick numbers was observed. In contrast, invasive alien species can reduce the quality of habitat for vector-host contacts, thus decreasing the risk. Japanese stiltgrass, *Microstegium vimineum*, changes soil surface microclimate conditions, reducing habitat quality for ticks (Civitello et al. 2008). Invasive alien species can also alter vector-host–pathogen dynamics, increasing human spillover. In Florida, the invasive alien Burmese python, *Python bivittatus*, heavily predated the large mammals of the area (i.e. deer, raccoons and opossums), inducing the native mosquito, *Culex cedecei*, to feed more on hispid cotton rat *Sigmodon hispidus* (the primary reservoir host) and even on humans (Hoyer et al. 2017).

## Conclusions and future directions

**Fig. 1:**
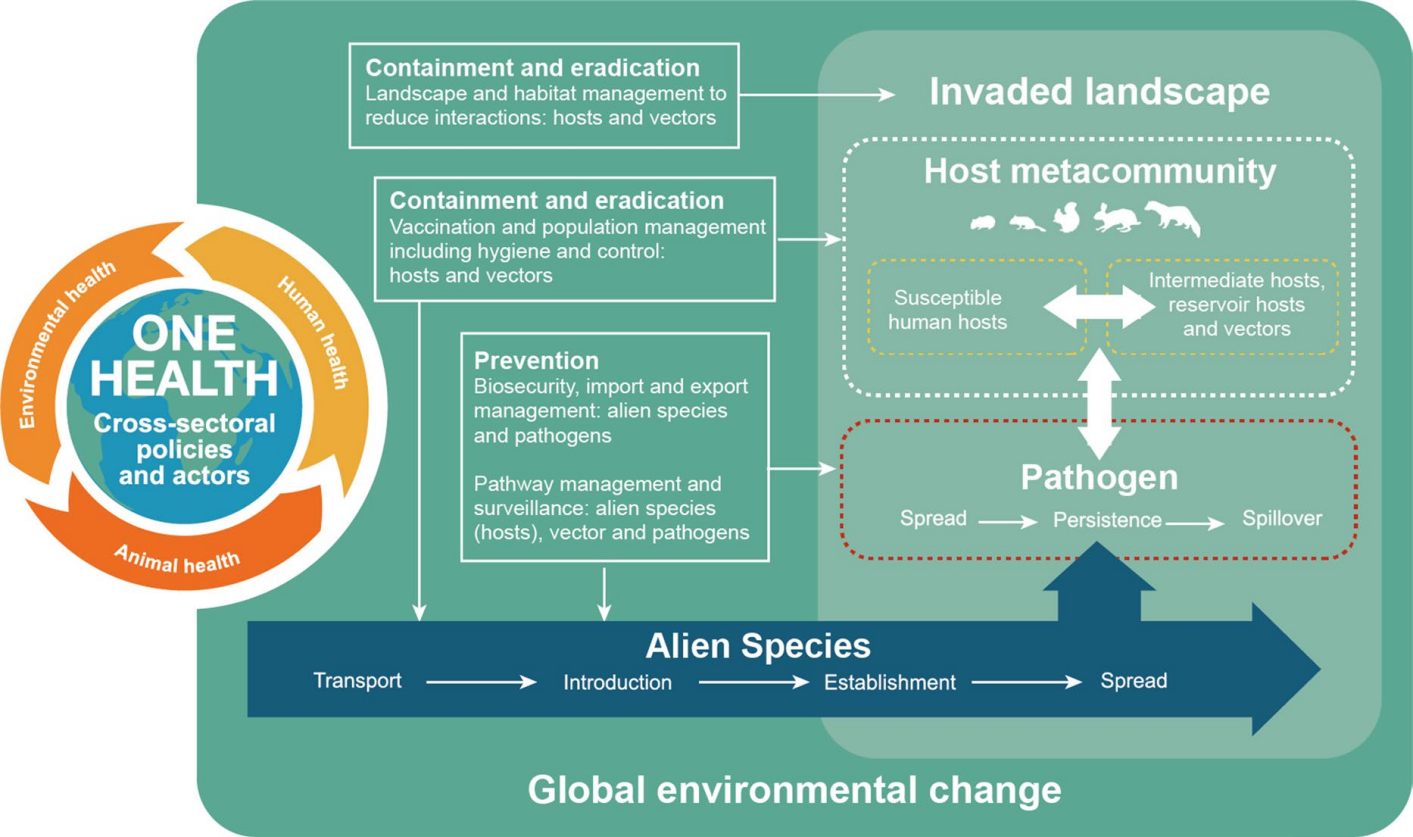
The role of invasive alien species in zoonotic disease transmission within the host metacommunity is modulated by interacting social, environmental and ecological factors operating in the invaded landscape and can be interrupted by cross-sectoral actors and policies operating on invasion or spill-over pathways, particularly if these are linked within One Health frameworks

Given that invasive alien species can play a role in transmission of zoonotic diseases that exceeds or is equivalent to the role of native wildlife, sometimes in a relatively short period following their arrival, there is an urgent need to raise awareness of the potential risks posed to human health. Ecological and social mechanisms govern the dynamics of zoonotic disease transmission but wildlife diseases are not consistently included within animal, plant and human policies (Roy et al. 2016); however the World Animal Health Organisation and Convention on International Trade in Endangered Species of Wild Fauna and Flora have agreed to collaborate on animal health and welfare issues (https://cites.org/eng/node/18857). Consideration of ecological, evolutionary and social perspectives will enhance understanding of the threat of biological invasions which adversely affect biodiversity and ecosystems but also affect human health (Fig. 1).

Most forecasts of the risk of emerging diseases neglect the potential role of alien species (Nuñez et al. 2020) and this consequently represents a gap in strategies underpinning responses for zoonoses. Modelling can inform prevention of zoonotic diseases and early warning of zoonotic pathogens carried or hosted by alien species (Cross et al. 2019). It is imperative to integrate biological invasion status and history into interpretation of host–pathogen networks, to better understand the role of invasive alien species in human spillover. Models that integrate heterogenous social and contact networks among wildlife, livestock and people (e.g. due to trade, social structure of animals and people, differential habitat use) are increasingly being used to understand infectious disease dynamics and spillover. Systems frameworks are being developed, that integrate the ecological, economic and social processes promoting spillover within ecosystems and policies and actors that interact with the disease systems in rapidly changing environments. It is critical to acknowledge and integrate the complex and diverse roles of invasive alien species into such model frameworks for zoonoses to better understand temporal and spatial trends in health risks to underpin public health reporting.

## Supplementary Information

Below is the link to the electronic supplementary material.

Supplementary file1 (DOCX 392 kb)

Supplementary file2 (DOCX 56 kb)

Supplementary file3 (DOCX 56 kb)

## Data Availability

All data generated or analysed during this study are included in this published article and its supplementary information files.

## Footnotes

1 Invasive alien species (IAS) are plants, animals and other organisms that are introduced directly or indirectly by people into places out of their natural range of distribution, and which may establish, spread and threaten or adversely impact upon biodiversity and nature’s contributions to people (including ecosystem services). Human health impacts are not always included in the definition of IAS but here we use a definition that extends to social and economic impacts including human health. There has been much debate over the inclusion of pathogens within the definition of IAS given the complexities of ascribing native or non-native status to microorganisms.

2 A zoonosis is a human disease occurring following the natural and reciprocal transmission of a pathogen or parasite between animals and humans.
