# Supplementary material for: The role of invasive alien species in the emergence and spread of zoonoses
Source: Biol Invasions. 2022 Dec 20;25(4):1249–64. doi: 10.1007/s10530-022-02978-1 (PMC9763809; doi:10.1007/s10530-022-02978-1)
Supplement: Supplementary file 1 — Supplementary file1 (DOCX 392 kb) [file 10530_2022_2978_MOESM1_ESM.docx]

Table 1. Alien arthropods as biological vectors for vector-borne zoonotic diseases (*=invasive alien species of Union concern). V = vector borne transmission by either flea (F), tick (T) or mosquito (MOS). Case fatality is mean of case fatalities reported by ECDC from 2012-2018 (where data are available). The criteria for actual or potential impact are outlined in Table 3.

| 1. **Arthropoda: Arachnida** | | | | | | | | | |
| --- | --- | --- | --- | --- | --- | --- | --- | --- | --- |
| Order: Family | Name of Alien Species | Pathogen genus impacted | | | | Evidence for role in zoonotic disease transmission | Countries of impact | Actual or potential impact  (certainty) | Refs |
|  |  | Species within genus | | Trans  mission | Case fatality rate (%) |  |  |  |  |
| Ixodida:  Ixodidae | *Rhipicephalus microplus* | *Candidatus Rickettsia senegalensis* | | V(F) | - | Pathogens found in 1-2 ticks of this IAS recovered from Philippine deer in invaded range | Guam | Potential  (very low) | (1) |
| Ixodida:  Ixodidae | *Hyalomma marginatum* | *Rickettsia aeschlimannii* | | V(T) | - | Adult *Hyalomma marginatum* tick positive for *Rickettsia aeschlimannii* | Austria | Potential  (very low) | (2) |
| Ixodida:  Ixodidae | *Rhipicephalus appendiculatus* | *Theileria parva*  (East Coast Fever) | | V(T) | - | Alien tick is a highly competent vector for East Coast fever, previously absent from island. Alien species introduction through cattle imports probably led to subsequent outbreaks | Comoros Islands, Indian Ocean | Actual  (high) | (3) |
| Ixodida:  Ixodidae | *Rhipicephalus sanguineus* | *Rickettsia conorii* | | V(T) | - | Implicated as only potential local vector of five cases of Mediterranean boutonneuse fever. Alien status in Switzerland is unclear | Switzerland | Actual  (low) | (4) |
| 1. **Arthropoda: Insecta** | | | | | | | | | |
| Order: Family | Name of Alien Species | | Pathogen genus impacted | | | Evidence for role in zoonotic disease transmission | Countries of impact | Actual or potential impact  (certainty) | Refs |
|  |  |  | Species within genus | Trans  mission | Case fatality rate (%) |  |  |  |  |
| Diptera: Culicidae | *Aedes aegypti* | | *Dirofilaria immitis Dirofilaria repens* | V(MOS) | - | Pathogens only rarely transmitted from dogs to humans. Non-infective larval stage of these pathogens found in adult *Ae. aegypti* in Argentina.  Laboratory colonies (Switzerland) found to be refractory to the pathogen | Argentina  Switzerland | Potential  (low) | (5,6)(7) |
| Diptera: Culicidae | *Culex pipiens* | | *Dirofilaria immitis Dirofilaria repens* | V(MOS) | - | Non-infective larval stage of these pathogens found in adult *Cx. pipiens* in Argentina | Argentina | Potential  (low) | (5,6) |
| Diptera: Culicidae | *Aedes japonicus* | | *Dirofilaria immitis Dirofilaria repens* | V(MOS) | - | Efficient a vector in the field for *both D. immitis* and *D. repens* as the native *Ae. geniculatus* (lab. competence of colony and field populations) | Switzerland | Potential  (medium) | (7) |
| Diptera: Culicidae | *Aedes albopictus* | | *Dirofilaria immitis Dirofilaria repens* | V(MOS) | - | The infective stage of this pathogen has been found in adult populations of *Ae. albopictus* in Italy and been anecdotally linked within an increase in human dirofilariosis | Italy | Actual  (low) | (8)(9) |
| Diptera: Culicidae | *Aedes aegypti* | | Eastern equine encephalitis virus | V(MOS) | - | Adults were found to acquire transmissible viral infections from infected starling hosts in the laboratory | United States | Potential  (low) | (10) |
| Diptera: Culicidae | *Aedes japonicus japonicus* | | Cache Valley virus | V(MOS) | - | In the lab, vector competence of *Ae. j. japonicus* mosquitoes was equivalent to other species that are part of the CVV transmission cycle | United States | Potential (high) | (11) |
| Diptera: Culicidae | *Aedes japonicus japonicus* | | West Nile Virus | V(MOS) | 9.13 | Introduced populations are highly susceptible to WNV, even more so than native vector *Culex pipiens*. Also feeds opportunistically on avian and mammalian hosts and highly abundant in late summer and autumn when human WNF cases occur, making it the ideal bridge vector | Switzerland | Potential (high) | (12,13) |
| Diptera: Culicidae | *Aedes japonicus japonicus* | | Dengue virus | V(MOS) | - |  |  |  | (14) |
| Diptera: Culicidae | *Aedes japonicus japonicus* | | Chikungunya virus | V(MOS) | - |  |  |  | (14) |
| Diptera: Culicidae | *Aedes japonicus japonicus* | | Zika virus | V(MOS) | - |  |  |  | (14) |
| Diptera: Culicidae | *Aedes albopictus* | | West Nile Virus | V(MOS) | 9.13 | *Ae. albopictus* has been found to be competent for West Nile Virus in laboratory and field populations in the US and Europe | Europe  United States |  | (15) |
| Diptera: Culicidae | *Aedes albopictus* | | Chikungunya virus | V(MOS) | - | Where *Ae. albopictus* is established, viraemic travel-related cases may generate local urban transmission of the virus as demonstrated by the sporadic events of chikungunya virus transmission since 2007. Field populations of the species have also been shown to be competent in the laboratory for this virus in Europe and the United States. | Europe  United States | Actual  (very high) | (16–19)  (9)  (20) |
| Diptera: Culicidae | *Aedes albopictus* | | Dengue Virus | V(MOS) | - | Where *Ae. albopictus* is established, viraemic travel-related cases may generate local urban transmission of the virus as demonstrated by the sporadic dengue outbreaks since 2010 in Europe. | France, Croatia | Actual  (very high) | (9) |
| Diptera: Culicidae | *Culex quinquefasciatus* | | West Nile Virus | V(MOS) | 9.13 | The species has established as a primary vector for WNV in the US, human cases shown to correlate with infection rates in this species | United States | Actual (medium) | (21) |

Table 2. Alien arthropods (Crustacea) as hosts for zoonotic diseases (*=invasive alien species of Union concern). C = transmission through contact. The criteria for actual or potential impact are outlined in Table 3.

| 1. **Maxillopoda** | | | | | | | | |
| --- | --- | --- | --- | --- | --- | --- | --- | --- |
| Order: Family | Name of Alien Species | Pathogen genus impacted | | | Evidence for role in zoonotic disease transmission | Countries of impact | Actual or potential impact  (certainty) | Refs |
|  |  | Species within genus | Trans  mission | Case fatality rate (%) |  |  |  |  |
| Sessilia: Austrobalanidae | *Austrominius (Elminius) modestus* | Colliforms | C | - | Coliform concentrations were significantly higher in the species than in the native *Mytilus edulis*, per unit area, across all surveyed sites. | United  Kingdom | Potential  (low) | (22,23) |

Table 3. Alien molluscs as hosts for zoonotic diseases (*=invasive alien species of Union concern). O =oral transmission through food (F) or water (W). Case fatality is mean of case fatalities reported by ECDC from 2012-2018 (where data are available). The criteria for actual or potential impact are outlined in Table 3.

| 1. **Gastropoda** | | | | | | | | |
| --- | --- | --- | --- | --- | --- | --- | --- | --- |
| Order: Family | Name of Alien Species | Pathogen genus impacted | | | Evidence for role in zoonotic disease transmission | Countries of impact | Actual or potential impact  (certainty) | Refs |
|  |  | Species within genus | Trans  mission | Case fatality rate (%) |  |  |  |  |
| Planorbidae | *Biomphalaria glabrata* | *Schistosoma mansoni* | O(W) | - | Intermediate hosts for *Schistosoma mansoni*  which itself is increasingly imported into Europe by immigrants from endemic areas | Romania | Potential  (low) | (24) |
| Planorbidae | *Biomphaliaria tenagophila* | *Schistosoma mansoni* | O(W) | - | Intermediate hosts for *Schistosoma mansoni*  which itself is increasingly imported into Europe by immigrants from endemic areas | Romania | Potential  (low) | (24) |
| Achatinidae | *Achatina fulica* | *Angiostrongylus cantonensis*  *A. costaricensis* | O(F) | - | Intermediate host for this pathogen (the rat lungworm), facilitating establishment in Florida | United States | Potential  (low) | (25) |
| Caenogastropoda: Thiaridae | *Melanoides tuberculata* | *Philophthalmus gralli*  *P. lucipetus*  *P. gralli* | O(W) | - | IAS established as an intermediate host for these eye trematodes. When definitive hosts were experimentally removed, pathogen prevalence decreased in the IAS intermediate host | Costa Rica  Peru  Brazil | Potential  (low) | (26)  (27)  (28) |
| Caenogastropoda: Thiaridae | *Melanoides tuberculata* | *Centrocestus formosanus* | O(F,W) | - | IAS infested with this pathogen so probably intermediate host | Brazil | Potential  (low) | (28) |
| Caenogastropoda: Thiaridae | *Melanoides tuberculata* | *Clonorchis sinensis* | O(F,W) | - | IAS established as an intermediate host though not yet linked to infections causing human cases in Brazil | Brazil | Potential  (low) | (28) |
| Caenogastropoda: Thiaridae | *Melanoides tuberculata* | *Haplorchis pumilio* | O(F) | - | IAS established as an intermediate host and linked to haplorchiasis in humans in Venezuela | Venezuela | Realised  (low) | (28) |
| Caenogastropoda: Thiaridae | *Melanoides tuberculata* | *Paragonimus westermani* | O(F) | - | IAS established as an intermediate host though not yet linked to infections causing human cases in Brazil | Brazil | Potential  (low) | (28) |
| Caenogastropoda: Thiaridae | *Melanoides tuberculata* | *Philophthalmus gralli* | O(W) | - | IAS infested with this pathogen so probably intermediate host | Brazil | Potential  (low) | (28) |
| Neritopsina: Helicinidae | *Alcadia striata* | *Angiostrongylus cantonensis* | O(F) | - | intermediate host for this pathogen (the rat lungworm), facilitating establishment in Florida | United States | Potential  (low) | (25) |
| Stylommatophora: Bradybaenidae | *Bradybaena similaris* | *Angiostrongylus cantonensis* | O(F) | - | intermediate host for this pathogen (the rat lungworm), facilitating establishment in Florida | United States | Potential  (low) | (25) |
| Stylommatophora: Bradybaenidae | *Zachrysia provisoria* | *Angiostrongylus cantonensis* | O(F) | - | intermediate host for this pathogen (the rat lungworm), facilitating establishment in Florida | United States | Potential  (low) | (25) |

Table 4. Alien fish species as hosts for food-borne zoonotic diseases (*=invasive alien species of Union concern). O = oral transmission through food (F) or water (W). Case fatality is mean of case fatalities reported by ECDC from 2012-2018 (where data are available). The criteria for actual or potential impact are outlined in Table 3.

| **Actinopterygii** | | | | | | | | |
| --- | --- | --- | --- | --- | --- | --- | --- | --- |
| Order: Family | Name of Alien Species | Pathogen genus impacted | | | Evidence for role in zoonotic disease transmission | Countries of impact | Actual or potential impact  (certainty) | Refs |
|  |  | Species within genus | Trans  mission | Case fatality rate (%) |  |  |  |  |
| Cichliformes: Cichlidae | *Oreochromis niloticus* | Echinostomatidae | O(F) | - | A very low prevalence (1.5%) of trematode infections found in farmed Nile tilapia (n=388) | China | Potential  (low) | (29) |
| Cichliformes: Cichlidae | *Oreochromis niloticus* | Heterophyidae | O(F) | - | A very low prevalence (1.5%) of trematode infections found in farmed Nile tilapia (n=388) | China | Potential  (low) | (29) |
| Cypriniformes: Cyprinidae | *Cyprinus carpio* | *Contracaecum bancrofti* | O(F,W) | - | Intermediate host for *Contracaecum bancrofti*, linked to human infections | Australia | Actual  (low) | (30) |
| Salmonidae | *Oncorhynchus keta* | *Anasakis simplex* | O(F) | - | Host for nematodes, which is prevalent in Baltic and Barent seas, causes anisakidosis | Europe | Actual  (low) | (31) |
| Salmonidae | *Oncorhynchus nerka* | *Anasakis simplex* | O(F) | - | Host for nematodes, which is prevalent in Baltic and Barent seas, causes anisakidosis | Europe | Actual  (low) | (31) |

Table 5. Alien bird species as hosts for zoonotic diseases. A = aerosol transmission. C= contact transmission. O = oral transmission through food (F) or water (W). V = vector-borne transmission by either tick (T) or mosquito (MOS). Case fatality is mean of case fatalities reported by ECDC from 2012-2018 (where data are available). The criteria for actual or potential impact are outlined in Table 3.

| **Aves** | | | | | | | | |
| --- | --- | --- | --- | --- | --- | --- | --- | --- |
| Order: Family | Name of Alien Species | Pathogen genus impacted | | | Evidence for role in zoonotic disease transmission | Countries of impact | Actual or potential impact  (certainty) | Refs |
|  |  | Species within genus | Trans  mission | Case fatality rate (%) |  |  |  |  |
| Passeriformes: Sturnidae | *Sturnus vulgaris* | *Salmonella enterica* | O(F) | 0.2 | The species is reservoir host. 2.5% prevalence in sampled individuals. | United States | Potential (medium) | (32) |
| Passeriformes: Sturnidae | *Sturnus vulgaris* | Eastern equine encephalitis (EEEV) virus | V(MOS) | - | The bird is likely maintenance host. Longer, more intense viremia than in other birds. Starlings frequently die as their viremia starts to wane, whereas other bird species generally survive at this stage. | United States | Potential (medium) | (10) |
| Anseriformes: Anatidae | *Branta canadensis* | *Chlamydia psittaci* | A | - | Role of IAS as host indicated by high prevalence (>90%) of *C. psittaci* antibodies, posing considerable risk of transmission. | Belgium | Potential (low) | (33) |
| Anseriformes: Anatidae | *Branta canadensis* | *Cryptosporidium sp.* | O(F,W) | 0.01 | Presence of Cryptosporidium found in free-living United Kingdom populations | United Kingdom | Potential (very low) | (34) |
| Galliformes: Phasianidae | *Alectoris chukar* | *Chlamydia psittaci* | A | - | Role of species as host indicated by high prevalence (>50%) of *C. psittaci* antibodies in farm setting | United States | Potential (low) | (35) |
| Psittaciformes: Psittacidae | *Myiopsitta monachus* | *Cryptosporidium spp.* | O(F,W) | 0.01 | It is a host, around 20% of sampled populations of this IAS harboured Cryptosporidium in Chile. Water acts as the primary source for contamination by *Cryptosporidium spp.* | Chile | Potential (low) | (36) |
| Psittaculidae | *Psittacula*  *krameri* | *Chlamydia psittaci* | A | - | IAS as host, low levels of shedding found in 20% of IAS individuals in urban park | France | Potential (low) | (37)  (38) |
| Psittaculidae | *Psittacula*  *krameri* | Influenza A virus (H9N2) | A;C | - | Parakeets imported (Pakistan to Japan) harbor H9N2 influenza A viruses, closely related to human strains from Hong Kong | Japan | Potential  (low) | (39) |
| Columbiformes: Columbidae | *Columba livia* | St. Louis encephalitis virus (SLEV) | V(MOS) | - | Alien bird is a host for the virus in peridomestic settings, 3.4% prevalence is greater than for native species | United States | Potential (low) | (40) |
| Columbiformes: Columbidae | *Columba livia* | western equine encephalomyelitis virus (WEEV) | V(MOS) | - | Alien bird is a host for the virus in peridomestic settings, though <1% prevalence, equivalent to native species | United States | Potential (low) | (40) |
| Columbiformes: Columbidae | *Streptopelia chinensis* | Japanese encephalitis virus (JEV)/ Saint Louise Encephalitis virus (SLEV) | V(MOS) | - | Potential host for virus, <1% of sampled populations had neutralising antibodies to JEV/SLEV in Hawaii | United States | Potential (very low) | (41) |
| Estrildidae | *Padda oryzivora* | Japanese encephalitis virus (JEV)/ Saint Louise Encephalitis virus (SLEV) | V(MOS) | - | Potential host for virus, <1% of sampled populations had neutralising antibodies to JEV/SLEV in Hawaii | United States | Potential (very low) | (41) |
| Passeriformes: Fringillidae | *Fringilla coelebs* | *Giardia* spp. | O | 0.05 | Alien bird is host, 60% of sampled birds (10) carried *Giardia spp.* in farmland settings | New Zealand | Potential (low) | (42) |
| Passeriformes: Turdidae | *Turdus philomelos* | Whataroa virus | V(MOS) | - | Whataroa virus is mosquito-borne and causes influenza like symptoms in humans. Antibodies found in 15% of sampled birds (>4000), suggesting IAS hosts plays maintenance role | United States | Potential (medium) | (43) |
| Passeriformes: Turdidae | *Turdus merula* | *Giardia* spp. | O | 0.05 | Alien bird is host, 35% of sampled birds (20) carried *Giardia* in farmland settings | New Zealand | Potential (low) | (42) |
| Passeriformes: Turdidae | *Turdus philomelos* | *Cryptosporidium spp.* | O(F,W) | 0.01 | Alien bird is host, 21.4% of sampled birds (14) carried *Cryptosporidium* in farmland settings | New Zealand | Potential (low) | (42) |
| Passeriformes: Turdidae | *Turdus philomelos* | *Giardia* spp. | O | 0.05 | Alien bird is host, 50% of sampled birds (14) carried *Giardia* spp. in farmland settings | New Zealand | Potential (low) | (42) |
| Passeriformes: Prunellidae | *Prunella modularis* | *Giardia* spp. | O | 0.05 | Alien bird is host, 14.3% of sampled birds (14) carried *Giardia spp.* in farmland settings | New Zealand | Potential (low) | (42) |
| Passeriformes: Passeridae | *Passer domesticus* | *Toxoplasma gondii* | O(F) | 2.11 | Alien bird is host, <2% of sampled birds were infected, much less than native hosts | United States | Potential (very low) | (44) |
| Passeriformes: Passeridae | *Passer domesticus* | *Cryptosporidium* spp. | O(F,W) | 0.01 | Alien bird is host, 8.2% of sampled birds (61) carried *Cryptosporidium spp.* in farmland settings | New Zealand | Potential (very low) | (42) |
| Passeriformes: Passeridae | *Passer domesticus* | *Giardia* spp. | O | 0.05 | Alien bird is host, 15.4% of sampled birds (104) carried *Giardia spp.* in farmland settings | New Zealand | Potential (low) | (42) |
| Passeriformes: Passeridae | *Passer domesticus* | Eastern equine encephalitis virus (EEEV) | V(MOS) | - | Sparrows found to be reservoir host for this mosquito-borne alphavirus equally susceptible to *Rattus* species, with viraemia lasting 4-5 days | United States | Potential (medium) | (45) |
| Passeriformes: Passeridae | *Passer domesticus* | Western equine encephalomyelitis virus (WEEV) | V(MOS) | - | Alien bird is a host for the virus in peridomestic settings, though <0.1% prevalence | United States | Potential (low) | (40) |
| Passeriformes: Passeridae | *Passer domesticus* | St. Louis encephalitis virus (SLEV) | V(MOS) | - | Alien bird is a host for the virus in peridomestic settings, though <1% prevalence | United States | Potential (low) | (40) |

Table 6. Invasive alien mammals as hosts for zoonotic diseases: A = aerosol transmission. C= contact transmission. O = oral transmission through food (F) or (W). V = vector-borne transmission by either flea (F), tick (T), mite (MI), lice (LI), biting flies (BF), Triatminae (Tri) or mosquitoes (MOS). Case fatality is mean of case fatality rate reported by ECDC from 2012-2018 (where data are available). The criteria for actual or potential impact are outlined in Table 3.

Key: Species in bold with * = IAS of Union concern, species underlined with # = IAS species listed in the Horizon scanning (46).

| **Mammalia** | | | | | | | | |
| --- | --- | --- | --- | --- | --- | --- | --- | --- |
| Order: Family | Name of Alien Species | Pathogen genus impacted | | | Evidence for role in zoonotic disease transmission | Countries of impact | Actual or potential impact  (certainty) | Refs |
|  |  | Species within genus | Trans  mission | Case fatality rate (%) |  |  |  |  |
| Artiodactyla: Suidae | *Sus scrofa* | *Brucella spp. (B. suis, B. canis* | O(F) | 0.73 | 4.3- 15% of samples tested positive  *Brucella* is a bacterium that causes brucellosis or undulant fever in humans. Hunters and those working in the livestock industries are particularly at risk. It should be noted that in Europe only an avirulent strain of *B. suis* is prevalent on wild-boars which cause abortions in pigs but a very limited number of humans cases (despite a huge exposure of hunters) in immunocompromised humans | United States  Georgia  Australia | Actual (medium) | (47)  (48)  (49)  (50)  (51–55) |
| Artiodactyla: Suidae | *Sus scrofa* | *Campylobacter spp. (Campylobacter coli, Campylobacter fetus, Campylobacter hyointestinalsis, Campylobacter jejuni, Campylobacter lanienae and Campylobacter sputorum)* | O(F) | 0.13 | Zero - 40% of samples tested positive. Food -borne *Campylobacter* infections are usually mild but considered the most common bacterial cause of human gastroenteritis in the world | United States  Georgia | Potential (medium) | (56)  (53) |
| Artiodactyla: Suidae | *Sus scrofa* | *Coxiella burnetii* | A;O(F) | 0.69 | 22- 50% of samples tested positive. Air- and food-borne infections of the bacterium.  *Coxiella burnetii* causes Q-fever. Some people are asymptomatic but others have flu-like symptoms. Livestock workers are particularly vulnerable | Australia  United States | Potential (medium) | (57)  (48) |
| Artiodactyla: Suidae | *Sus scrofa* | *Cryptosporidium spp. (C. scrofarum and C. suis)* | O(F,W) | 0.01 | 1.6-5.4% of feral pigs were shedding oocysts.  Water- and food-borne infections of the bacterium *Cryptosporidium* sp. cause gastroenteritis. Contamination of water sources is seen as increasingly problematic | United States | Potential (very low) | (58)  (59) |
| Artiodactyla: Suidae | *Sus scrofa* | *Ehrlichia chaffeensis* | V(T) | - | Possibility of antibodies in feral pigs.  Tick-borne bacterium that can cause Ehrlichiosis in humans which results in mild to moderate flu-like symptoms | United States | Potential (very low) | (60) |
| Artiodactyla: Suidae | *Sus scrofa* | *Escherichia coli* | O(F) | 0.3 | Presence in 28% of samples  Detection of the bacterium in *S. scrofa* at higher levels in agricultural regions compared to forested regions | Georgia | Potential (low) | (53) |
| Artiodactyla: Suidae | *Sus scrofa* | *Giardia sp.* | O | 0.05 | 4.3 – 7.6% of feral pigs were shedding *Giardia*.  Water- and food-borne infections can occur from a variety of livestock. Feral *S. scrofa* maybe a reservoir of *Giardia* transmitting to humans and livestock | United States | Potential (very low) | (58)  (59) |
| Artiodactyla: Suidae | *Sus scrofa* | Hepatitis E | O(F) | - | 4.4% seroprevalence  Low prevalence of this virus in feral *S. scrofa*  Feral swine acts as viral reservoir, transmission to humans by consumption of swine | United States | Potential  (very low) | (50) |
| Artiodactyla: Suidae | *Sus scrofa* | Influenza (H1N1, H3N2, Influenza A) | A;C | - | H1N1 virus has been detected in feral *S. scrofa*. 10.8% seroprevalence for influenza A  Multiple strains can circulate in swine simultaneously | United States | Potential (low) | (61)  (62)  (50) |
| Artiodactyla: Suidae | *Sus scrofa* | *Leptospira spp. (L. pomona, L. hardjo, L. interrogans, Leptospira interrogans serovar Pomona, Leptospira borgpetersenii serovar tarassovi* | A;C;O(W) | 3.08 | 10 - 87% seroprevalence.  Leptospirosis is a severe flu-like illness caused by bacteria in the genus *Leptospira*. Increased seroprevalence of *Leptospira* spp. occurred in years preceded by flooding and high rodent abundance in Australia, suggesting the potential for zoonotic infection is much greater than previously realised | Australia  United States | Potential (medium) | (52)  (48)  (52)  (63)  (64) |
| Artiodactyla: Suidae | *Sus scrofa* | *Mycobacterium avium* | A | - | 85% seroprevalence  Tuberculosis in humans due to *M. avium* most often occur in immunocompromised individuals | United States | Potential (medium) | (48) |
| Artiodactyla: Suidae | *Sus scrofa* | Pseudorabies virus (PRV) | A;O | - | 2.52 - 3% seroprevalence  PRV primarily infects swine and has several secondary hosts, including cattle, dogs, and cats. It may infect humans. Higher prevalence of pseudorabies in feral *S. scrofa* hunted with dogs compared to other harvesting methods | United States | Potential (very low) | (48)  (55)  (54) |
| Artiodactyla: Suidae | *Sus scrofa* | *Salmonella* sp. | O(F) | 0.2 | 50% of animals possessed antibodies for *Salmonella* sp. | United States | Potential (low) | (65) |
| Artiodactyla: Suidae | *Sus scrofa* | *Streptococcus suis* | O(F) | - | 94.4% of animals tested positive for *Streptococcus suis*. It should be noted that human infection can be severe, with a short incubation leading to death. Few cases have been recorded in meat production industry. Very few cases in hunters | United States | Potential (low) | (65) |
| Artiodactyla: Suidae | *Sus scrofa* | *Toxoplasma gondii* | O(F) | 2.11 | Infection levels ranged from 11% to 27.7%  Protozoan that can infect humans causing toxoplasmosis, most humans are asymptomatic but some have flu-like symptoms. More serious complications occur in immunocompromised individuals. lack of sanitary management of feral animals increases the incidence of infections, and the consumption of raw or inadequately cooked meat may become a potential source of infection for humans | Brazil  United States | Potential (low) | (66)  (67)  (48)  (68)  (69)  (70) |
| Artiodactyla: Suidae | *Sus scrofa* | *Trichnella sp. (T. spiralis)* | O(F) | 0.42 | Prevalence was low ranging from 1.8 to 13.3%  Food-borne transmission of this nematode can cause Trichinellosis which is widely distributed worldwide | Chile  United States | Potential (low) | (67)  (50)  (71)  (72)  (70)  (69) |
| Artiodactyla: Suidae | *Sus scrofa* | *Trypanosoma cruzi* | V(TRI) | - | 6% of *S. scrofa*, reservoir for the protozoan, tested positive.  Eleven species of kissing bugs (Hemiptera: Reduviidae) are found in the United States with ranges possibly expanding northward. At least eight of the species, perhaps all, vector *Trypanosoma cruzi*, the cause of Chagas disease. Infection can also occur through food- or water-borne transmission | United States | Potential (low) | (73) |
| Artiodactyla: Suidae | *Sus scrofa* | *Yersinia pestis* | A;V(FL) | - | 15% of *S. scrofa* tested positive for *Y. pestis*.  Plague causing bacterium transmitted by fleas. It should be noted that comparison between continents is difficult because of differences in the epidemiological contexts | United States | Potential (very low) | (48) |
| Carnivora: Canidae | ***Nyctereutes procyonoides**** | *Alaria alata* | O(F) | - | 30 – 94.3% infection of the trematode. | Poland  Estonia  Denmark  Lithuania  Austria | Potential  (low) | (74)  (75)  (76)  (77)  (78) |
| Carnivora: Canidae | ***Nyctereutes procyonoides**** | *Anaplasma phagocytophilum* | V(T) | - | Bacterium spread by ticks to humans causing anaplasmosis. 23% infection of raccoon dogs compared to 8.2% of red foxes | Germany | Potential (low) | (79) |
| Carnivora: Canidae | ***Nyctereutes procyonoides**** | *Apophallus sp.* | O(F) | - | 15.1% intestinal helminth infection of raccoon dogs compared to 7.6% of red foxes | Poland | Potential (low) | (74) |
| Carnivora: Canidae | ***Nyctereutes procyonoides**** | *Echinococcus multilocularis* | O | 0.64 | Low prevalence with 1.6% in Estonia, 6.6-21.1% in Latvia, 8.2% in Lithuania, 5.1-8.0% in Poland and 2.7-4.8% in Germany. Moderate prevalence in Slovakia (50% but two individuals sampled) | Poland  Germany  Netherlands  Estonia | Potential (very low) | (74)  (80)  (81)  (82) |
| Carnivora: Canidae | ***Nyctereutes procyonoides**** | *Francisella tularensis* | V(T,BF,MOS) | 3.05 | Lower prevalence of bacterium in raccoon dog (12.8%) than red fox (18.4%). Pathogen is the causative agent for tularemia | Germany | Potential (low) | (83,84) |
| Carnivora: Canidae | ***Nyctereutes procyonoides**** | *Ancylostoma spp.* | C | - | Higher prevalence of hookworm in raccoon dog (83%) than red fox (68.2%) | Poland | Potential (medium) | (74) |
| Carnivora: Canidae | ***Nyctereutes procyonoides**** | *Mesocestoides spp.* | O | - | Lower prevalence of helminth in raccoon dog (24.5%) compared to red fox (57.6%). Only approximately 3% of raccoon dogs infected in Germany | Poland  Germany | Potential (very low) | (74)  (85) |
| Carnivora: Canidae | ***Nyctereutes procyonoides**** | Rabies (Lyssavirus *spp.*) | C | 100 | Prevalence of rabies increased from 11.8% in 1994 to 28.9% in 2004 in raccoon dogs in Lithuania. Variable prevalence across studies from 0-100% infection in samples. However, raccoon dog is now a new maintenance host of rabies in North-East of Europe but the number of human cases in Europe remains less than one per year | Estonia  Lithuania  Latvia  Poland | Potential (medium) | (86)  (87)  (88)  (89)  (90)  (91)  (92) |
| Carnivora: Canidae | ***Nyctereutes procyonoides**** | *Taenia spp.* | O(F) | - | Lower prevalence of helminth in raccoon dog (1.9%) compared to red fox (40.9%) | Poland | Potential (very low) | (74) |
| Carnivora: Canidae | ***Nyctereutes procyonoides**** | *Toxocara/Toxascaris* nematodes | O | - | Lower prevalence of helminth in raccoon dog (15.1%) compared to red fox (33.3%) | Poland | Potential (very low) | (74) |
| Carnivora: Canidae | ***Nyctereutes procyonoides**** | Trematodes, cestodes, nematodes | - | - | 4 trematodes, 4 cestodes and 9 nematodes. Number and range of raccoon dogs in Europe and the relatively high number of zoonotic pathogen taxa that it harbours suggests that this species should be considered an important source of environmental contamination | Estonia | Potential (low) | (75) |
| Carnivora: Canidae | ***Nyctereutes procyonoides**** | *Trichinella spiralis (also T. britovi, T. nativa, T. pseudospiralis)* | O(F) | 0.42 | Higher prevalence of *Trichinella* sp. in foxes (28.9–40.6%) than raccoon dogs (32.5–42%) across Lithuania, Latvia and Estonia. In Poland only 0.8% infection rate; 5% in Germany; 1 out of 9 raccoon dogs in the Netherlands | Lithuania  Latvia  Estonia  Poland  Germany  Netherlands | Potential (medium) | (93)  (94)  (72)  (81)  (95)  (96)  (97) |
| Carnivora: Canidae | ***Nyctereutes procyonoides**** | *Uncinaria stenocephala* | O | - | 97.6% infection in hunted raccoon dogs. Infection intensity determined by weight | Estonia | Potential (medium) | (75) |
| Carnivora: Canidae | *Canis lupus* | *Echinococcus granulosus* | O | 0.64 | 2.2% faecal samples tested positive for *E. granulosus* | Estonia | Potential (very low) | (98) |
| Carnivora: Canidae | *Canis lupus familiaris* | *Leptospira spp. (L. mayottensis, L. borgpetersenii, L. kirschneri)* | A;C;O(W) | 3.08 | Reservoir of *L. mayottensis*; main reservoirs of L. borgpetersenii and *L. kirschneri*, both bacteria being prevalent in local clinical cases | Madagascar | Actual (medium) | (99) |
| Carnivora: Canidae | *Canis lupus dingo* | *Coxiella burnetii* | A;O(F) | 0.69 | Lower prevalence (17.3%) of the bacterium in dingoes compared to native species *Isoodon macrourus* (23.9%) but higher than possum (10.7%) | Australia | Potential (low) | (57) |
| Carnivora: Canidae | *Vulpes vulpes* | *Bartonella henselae, Bartonella clarridgeiae* | V(F) | - | *B. henselae* and *B. clarridgeiae* were detected in fleas (*Ctenocephalides felis*) from red foxes (*Vulpes vulpes*) | Australia | Potential (very low) | (100) |
| Carnivora: Canidae | *Vulpes vulpes* | *Coxiella burnetii* | A;O(F) | 0.69 | Higher prevalence (43.8%) of the bacterium in foxes compared to native species *Isoodon macrourus* (23.9%) and possum (10.7%) | Australia | Potential (medium) | (57) |
| Carnivora: Canidae | *Vulpes vulpes* | *Echinococcus granulosus* | O | 0.64 | Presence only cited, widely spread tapeworm of domestic dogs | Australia | Potential (very low) | (101) |
| Carnivora: Felidae | *Felis catus* | *Helminths (Angiostrongylus cantonensis, Toxocara cati, Ancylostoma braziliense, Taenia taeniaeformis, Moniliformis moniliformis, Hymenolepis nana)* | - | - | Sixty-one (92%) of cats harboured one or more helminth species | Australia | Potential (medium) | (102) |
| Carnivora: Felidae | *Felis catus* | *Coxiella burnetii* | A;O(F) | 0.69 | Higher prevalence (38.7%) of the bacterium in cats compared to native species *Isoodon macrourus* (23.9%) and possum (10.7%) | Australia | Potential (medium) | (57) |
| Carnivora: Felidae | *Felis catus* | *Leptospira* spp. | A;C;O(W) | 3.08 | *Leptospira spp.* was detected in the 42.4% of sampled cats in Christmas Island, but no in cats sampled in Dirk Hartog Island or southwest Western Australia | Australia | Potential (low) | (102) |
| Carnivora: Felidae | *Felis catus* | *Toxoplasma* sp. *(Toxoplasma gondii)* | O(F) | 2.11 | Antibody test to *Toxoplasma sp.,* yielded a prevalence rate of about 16%. In a further study DNA of *Toxoplasma gondii* was detected in 5% of faecal samples | United States | Potential (low) | (103) (104) |
| Carnivora: Mustelidae | *Neovison vison* | Influenza A | A;C | - | 2.2% seropositive | Spain | Potential (very low) | (105) |
| Carnivora: Mustelidae | *Neovison vison* | *Ascaridinae* | O(F,W) | - | Presence only cited | Chile | Potential (very low) | (106) |
| Carnivora: Mustelidae | *Neovison vison* | *Cryptosporidium* sp. | O(F,W) | 0.01 | Presence only cited | Chile | Potential (very low) | (106) |
| Carnivora: Mustelidae | *Neovison vison* | *Echinococcus* spp*.* | O | 0.64 | Presence only cited. Average seropositivity 14.2% for echinococcosis | Poland | Potential (very low) | (107) |
| Carnivora: Mustelidae | *Neovison vison* | *Leptospira* spp. *(L. interrogans, L. borgpetersenii)* | A;C;O(W) | 3.08 | 54.6% of samples. Lakes and rivers also contaminated | Chile  Patagonia | Potential (medium) | (106)  (108) |
| Carnivora: Mustelidae | *Neovison vison* | *Pterygodermatites (Paucipectines)* spp*.* | O | - | Presence only cited | Chile | Potential (very low) | (106) |
| Carnivora: Mustelidae | *Neovison vison* | *Toxocara* sp. | O | - | Evidence of increase in seropositivity over time for *Toxocara sp.* Average seropositivity 21.7% for toxocarosis | Poland | Potential (low) | (107) |
| Carnivora: Mustelidae | *Neovison vison* | *Toxoplasma gondii* | O(F) | 2.11 | 78.8% were seropositive | Spain | Potential (medium) | (109) |
| Carnivora: Procyonidae | ***Procyon lotor**** | Influenza H5N1 | A;C | - | Presence only cited | Japan | Potential (very low) | (110) |
| Carnivora: Procyonidae | ***Procyon lotor**** | *Baylisascaris procyonis* | O | - | 1.9 – 100 % prevalence of the helminth as it has a broad range of paratenic hosts which are affected significantly by the migrating larval stages of the parasite. | Denmark  Germany  Poland  Norway  China  Japan | Potential (medium) | (111)  (112)  (113)  (114)  (115)  (116)  (117)  (118)  (119) |
| Carnivora: Procyonidae | ***Procyon lotor**** | *Campylobacter* sp. | O(F) | 0.13 | 1.3% prevalence. Principal reservoirs of the pathogen found in wild mammals and birds with occurrence higher in birds. | Japan | Potential (very low) | (120) |
| Carnivora: Procyonidae | ***Procyon lotor**** | *Borrelia afzelii; Borrelia garinii* | V(T, LI) | - | 0.9% prevalence | Japan | Potential (very low) | (121) |
| Carnivora: Procyonidae | ***Procyon lotor**** | *Capillaria* sp*.* | O | - | 3.2% prevalence | Poland | Potential (very low) | (115) |
| Carnivora: Procyonidae | ***Procyon lotor**** | *Cryptosporidium* sp*.* | O(F,W) | 0.01 | 34.7% prevalence with variance in infection rate between female (38.9%) and male (32.3%) | Germany; Poland | Potential (medium) | (122) |
| Carnivora: Procyonidae | ***Procyon lotor**** | *Enterocytozoon bieneusi* | O(W) | - | 4.1% prevalence. Classed as major microsporidians to infect humans and animals globally. 240 different genotypes of *E. bieneusi* identified and split into eight different groups. | Germany; Poland | Potential (very low) | (122) |
| Carnivora: Procyonidae | ***Procyon lotor**** | *Francisella tularensis* | V(T,BF,MOS) | 3.05 | 0.5% prevalence | Japan | Potential (very low) | (121) |
| Carnivora: Procyonidae | ***Procyon lotor**** | *Listeria* spp*.* | O(F) | 15.91 | 2 - 5% in positive isolates found  Amongst all species of *Listeria* spp., *L. monocytogenes & L. ivanovii* are regarded as “potentially pathogenic” | Poland | Potential (very low) | (123) |
| Carnivora: Procyonidae | ***Procyon lotor**** | *Molineus* spp. | O | - | Presence only cited | Austria | Potential (very low) | (78) |
| Carnivora: Procyonidae | ***Procyon lotor**** | *Orientia tsutsugamushi* | V(MI) | - | 1.4% prevalence | Japan | Potential (very low) | (121) |
| Carnivora: Procyonidae | ***Procyon lotor**** | *Rickettsia japonica* | V(T) | - | 7.3% prevalence | Japan | Potential (low) | (121) |
| Carnivora: Procyonidae | ***Procyon lotor**** | *Salmonella* sp*. (S. enterica)* | O(F) | 0.2 | 2 – 5.7% prevalence in urban and suburban areas. | Japan  Poland | Potential (very low) | (120)  (123) |
| Carnivora: Procyonidae | ***Procyon lotor**** | *Sarcocystis kirkpatricki* | O(F) | - | 8.3% prevalence in racoons and considered to be intermediate hosts of the pathogen. | Germany | Potential (low) | (124) |
| Carnivora: Procyonidae | ***Procyon lotor**** | *Staphylococcus* spp. | A;C | - | 35% prevalence. Species in this genus were coagulase-positive and isolated most frequently. | Poland | Potential (medium) | (123) |
| Carnivora: Procyonidae | ***Procyon lotor**** | *Strongyloides procyonis* | C | - | 28.3% prevalence and infection of one person, threadworm found in raccoons | Japan | Actual (medium) | (125) |
| Carnivora: Procyonidae | ***Procyon lotor**** | *Toxascaris* sp. | O | - | 2 farmed raccoons | Norway | Potential (very low) | (116) |
| Carnivora: Procyonidae | ***Procyon lotor**** | *Toxocara* sp. | O | - | 2 farmed raccoons | Norway | Potential (very low) | (116) |
| Carnivora: Procyonidae | ***Procyon lotor**** | *Yersinia* spp. | A;O(W)(F);V(F) | 0.07 | 7 - 38.6% prevalence. All virulent strains found were *Y. pseudotberculosis* with all isolates belonging to the Far Eastern systemic pathogenicity type | Japan  Poland | Actual (medium) | (120)  (123) |
| Rodentia: Cricetidae | ***Ondatra zibethicus**** | *Anoplocephalidae* | O | - | Adult cestodes found 1.5% prevalence in the small intestine of sampled muskrats | Germany | Potential (very low) | (126) |
| Rodentia: Cricetidae | ***Ondatra zibethicus**** | *Cryptosporidium parvum* | O(F,W) | 0.01 | Heavily infested individuals. Infection in investigated rodent species were asymptomatic | Poland | Potential (very low) | (127) |
| Rodentia: Cricetidae | ***Ondatra zibethicus**** | *Echinococcus multilocularis* | O | 0.64 | 0.7 – 4.1% prevalence | Germany  France | Potential (very low) | (126)  (128)  (129) |
| Rodentia: Cricetidae | ***Ondatra zibethicus**** | *Giardia* spp. | O | 0.05 | 75.2% prevalence | Germany | Potential (medium) | (130) |
| Rodentia: Cricetidae | ***Ondatra zibethicus**** | Hantavirus | A;C | - | 8% of tested animals (n=266) were found positive to infection; 14% had antibodies | Germany | Potential (low) | (131) |
| Rodentia: Cricetidae | ***Ondatra zibethicus**** | *Leptospira* spp. | A;C;O(W) | 3.08 | Presence only cited | France | Potential (very low) | (132) |
| Rodentia: Cricetidae | ***Ondatra zibethicus**** | *Taenia* spp*. (T. taeniaeformis, T. crassiceps, T. polyacantha, T. martis)* | O(F) | - | 0.4 – 42.3% metacestodes with *T. taeniaeformis* at highest prevalence | France  Germany | Potential (medium) | (129)  (126) |
| Rodentia: Echimyidae | ***Myocastor coypus**** | *Aeromonas* spp*. (A. hydrophila, A. caviae, and A. dhakensis)* | O(W) | - | Presence only cited | Korea | Potential (very low) | (133) |
| Rodentia: Echimyidae | ***Myocastor coypus**** | *Echinococcus multilocularis* | O | 0.64 | >1% (2 of 531 individuals) | France | Potential (very low) | (129) |
| Rodentia: Echimyidae | ***Myocastor coypus**** | *Strongyloides myopotami* | C | - | 99% prevalence | Japan | Potential (medium) | (134) |
| Rodentia: Echimyidae | ***Myocastor coypus**** | *Taenia* sp. *(T. taeniaeformis)* | O(F) | - | 3.8% prevalence | France | Potential (very low) | (129) |
| Rodentia: Echimyidae | ***Myocastor coypus**** | *Toxoplasma gondii* | O(F) | 2.11 | 59.4% prevalence with higher incidence in male (68.2%) compared to female (31.8%) individuals | Italy | Potential (medium) | (135) |
| Rodentia: Muridae | *Mus musculus* | *Bartonella* sp. | V(F) | - | Presence only cited | Senegal | Potential (very low) | (136) |
| Rodentia: Muridae | *Mus musculus* | *Brucella* sp. | C; O(F) | 0.73 | Brucella was not identified, and it was concluded that prevalence was below 12% | Australia | Potential (low) | (137) |
| Rodentia: Muridae | *Mus musculus* | *Cryptosporidium spp.* | O(F,W) | 0.01 | 11.8% prevalence | New Zealand | Potential (low) | (42) |
| Rodentia: Muridae | *Mus musculus* | *Giardia* spp. | O | 0.05 | 30.5% prevalence | New Zealand | Potential (medium) | (42) |
| Rodentia: Muridae | *Mus musculus* | *Hantavirus* | A;C | - | Presence only cited | Senegal | Potential (very low) | (138) |
| Rodentia: Muridae | *Mus musculus* | *Hepatozoon canis* | V(T) | - | Presence only cited but notes lower levels of pathogen prevalence compared to native species sampled in same study | Senegal | Potential (very low) | (139) |
| Rodentia: Muridae | *Mus musculus* | *Helminths* | - | - | 8% were infected with *Syphacia obvelata*, 8% with *Hetereakis spumosa*, 12% with *Taenia taeniaeformis*, 36% with *Nippostrongylus brasiliensis* | Argentina | Potential (low) | (140) |
| Rodentia: Muridae | *Mus musculus* | *Leptospira* spp. | A;C;O(W) | 3.08 | 23 – 59% prevalence. Thought to be the most widespread zoonotic disease. Animals in agricultural areas were three times more likely to carry the pathogen than those in wild areas | Madagascar  Puerto Rico  Chile | Potential (medium) | (141)  (142)  (143) |
| Rodentia: Muridae | *Mus musculus* | *Toxoplasma gondii* | O(F) | 2.11 | 11% prevalence | Senegal  United States | Potential (low) | (144)  (44) |
| Rodentia: Muridae | *Mus musculus* | *Trypanosoma lewis* | V(F) | - | 8.6% prevalence | Nigeria | Potential (low) | (145) |
| Afrosoricida:  Tenrecidae | *Tenrec ecaudatus* | *Leptospira mayottensis* | A;C;O(W) | 3.08 | 27% prevalence; acute human infections reported | Mayotte (Indian Ocean Islands) | Actual (medium) | (99) |
| Carnivora: Herpestidae | ***Herpestes javanicus**** | Hepatitis E virus (HEV) | O(F) | - | 21% prevalence | Japan | Potential (medium) | (146) |
| Carnivora: Mustelidae | *Mustela putorius furo* | *Giardia* spp. | O | 0.05 | 33.3% prevalence | New Zealand | Potential (medium) | (42) |
| Carnivora: Procyonidae | ***Nasua nasua**** | *Coccidia* | O | - | 50% prevalence | Norway | Potential (medium) | (116) |
| Carnivora: Therapsid | *Felis catus* | *Alaria alata* | O(F) | - | 3% prevalence and increasing in wild boar | Denmark | Potential (very low) | (147) |
| Carnivora: Viverridae | *Paguma larvata* | *Campylobacter* spp. | O(F) | 0.13 | 7.2% prevalence | Japan | Potential (low) | (120) |
| Carnivora: Viverridae | *Paguma larvata* | *Salmonella enterica* | O(F) | 0.2 | 2% prevalence | Japan | Potential (very low) | (120) |
| Carnivora: Viverridae | *Paguma larvata* | *Yersinia* spp. | A;O(W)(F);V(F) | 0.07 | 10.5% prevalence | Japan | Potential (low) | (120) |
| Didelphimorphia: Didelphidae | *Didelphis marsupialis* | *Rickettsia typhi* | V(F) | - | 10.6% prevalence | United States (California) | Potential (low) | (148) |
| Diprotodontia: Phalangeridae | *Trichosurus vulpecula****^#^*** | *Cryptosporidium spp.* | O(F,W) | 0.01 | 12.8% prevalence, lower than *Giardia* spp. | New Zealand | Potential (low) | (42) |
| Diprotodontia: Phalangeridae | *Trichosurus vulpecula^#^* | *Giardia* spp. | O | 0.05 | 23.6% prevalence, no significant difference between wild animals on farmland and wild animals in the wild | New Zealand | Potential (medium) | (42) |
| Eulipotyphla: Erinaceidae | *Erinaceus europaeus* | *Giardia* spp. | O | 0.05 | 33.3% prevalence | New Zealand | Potential (medium) | (42) |
| Lagomorpha: Leporidae | *Lepus europaeus* | *Fasciola hepatica* | O(F,W) | - | 14.2% prevalence. Pathogen has ability to develop fully in wild hares, shedding normal eggs in the faeces | Patagonia | Potential (low) | (149) |
| Lagomorpha: Leporidae | *Oryctolagus cuniculus* | *Giardia* spp*.* | O | 0.05 | 20% prevalence | New Zealand | Potential (low) | (42) |
| Primates: Cercopithecidae | *Chlorocebus aethiops sabaeus* | *Klebsiella pneumoniae* | A | - | 20% prevalence, causing severe abscesses in primates | St Kitts (Caribbean islands) | Potential (low) | (150) |
| Primates: Cercopithecidae | *Macaca mulatta* | B-virus | C | - | Around 70% prevalence | Puerto Rico | Potential (high) | (151) |
| Primates: Cercopithecidae | *Macaca mulatta* | macacine herpesvirus 1 (McHV-1) | C | - | 25% prevalence | United States (Florida) | Potential (medium) | (152) |
| Rodentia: Chinchillidae | *Chinchilla lanigera* | *Taenia crassiceps* | O(F) | - | One individual infected | Switzerland | Potential (very low) | (153) |
| Rodentia: Muridae | *Gerbillus nigeriae* | *Borrelia crocidurae* | V(T, LI) | - | 5% prevalence | Senegal | Potential (low) | (139) |
| Rodentia: Muridae | *Herpestes auropunctatus^#^* | *Leptospira spp.* | A;C;O(W) | 3.08 | 13% prevalence | Puerto Rico | Potential (low) | (142) |
| Rodentia: Muridae | *Herpestes auropunctatus^#^* | *Leptospira spp.* | A;C;O(W) | 3.08 | 8.1% prevalence | St Kitts (Caribbean islands) | Potential (low) | (154) |
| Rodentia: Soricidae | *Suncus murinus* | *Leptospira* spp*.* | A;C;O(W) | 3.08 | 25% prevalence | Madagascar | Potential (medium) | (141) |
| Rodentia: Sciuridae | *Callosciurus finlaysonii^#^* | variegated squirrel bornavirus 1 (VSBV-1) | A | - | 16.7% prevalence | Germany | Potential (low) | (155) |
| Rodentia: Sciuridae | *Callosciurus prevostii* | variegated squirrel bornavirus 1 (VSBV-1) | A | - | 17.3% prevalence | Germany; Croatia | Potential (low) | (155) |
| Rodentia: Sciuridae | ***Sciurus carolinensis**** | *Borrelia burgdorferi Sensu Lato* | V(T) | - | 11.9% prevalence. Pathogen is an agent for Lyme borreliosis. Invasive rodent species shown to be high epidemiological importance and can support species-specific host strains. | Scotland | Potential (low) | (156) |
| Rodentia: Sciuridae | ***Sciurus carolinensis**** | Tick-borne encephalitis virus (TBEV) | V(T) | - | 1.9-2.5% prevalence. Considered as an emerging flavivirus in Europe. | Italy | Potential (very low) | (157) |
| Rodentia: Sciuridae | ***Sciurus carolinensis**** | Usutu virus (USUV) | V(MOS) | - | 3.2-3.8% prevalence. Considered as an emerging flavivirus in Europe. | Italy | Potential (very low) | (157) |
| Rodentia: Sciuridae | ***Sciurus carolinensis**** | West Nile virus (WNV) | V(MOS) | - | 0.6% prevalence. Considered as an emerging flavivirus in Europe. | Italy | Potential (very low) | (157) |
| Rodentia: Sciuridae | ***Sciurus carolinensis**** | variegated squirrel bornavirus 1 (VSBV-1) | A | - | 50% prevalence. Borna virus found in a captive colony. The virus is indigenous and carried by a shrew. | Germany | Potential (high) | (155) |
| Rodentia: Sciuridae | *Sciurus variagata* | variegated squirrel bornavirus 1 (VSBV-1) | A | - | Detection of pathogen in people that is same as pathogen in exotic squirrels. Borna virus found in a captive colony. The virus is indigenous and carried by a shrew | Germany | Potential  (low) | (158) |
| Rodentia: Sciuridae | ***Tamias sibiricus barberi**** | *Borrelia burgdorferi* species complex | V(T) | - | Greater flea infestation in alien species (no reported values) | France | Potential (medium) | (159) |
| Rodentia: Sciuridae | *Tamiops swinhoei* | variegated squirrel bornavirus 1 (VSBV-1) | A | - | 1.3% prevalence | Germany | Potential (very low) | (155) |
| Rodentia: Muridae | *Rattus norvegicus* | *Anaplasma phagocytophilum* | V(T) | - | Presence only cited | United States | Potential (very low) | (160) |
| Rodentia: Muridae | *Rattus norvegicus* | *Ehrlichia muris* | V(T) | - | Presence only cited | United States | Potential (very low) | (161) |
| Rodentia: Muridae | *Rattus norvegicus* | *Angiostrongylus cantonensis* | O(F) | - | Presence only cited | Grenada | Potential (very low) | (162) |
| Rodentia: Muridae | *Rattus norvegicus* | *Aspiculuris tetraptera* | O(F) | - | Presence only cited | South Africa | Potential (very low) | (163) |
| Rodentia: Muridae | *Rattus norvegicus* | *Eucoleus sp.* | O | - | Proportional abundance 3.95% Prevalence >0.1% | South Africa | Potential (very low) | (163) |
| Rodentia: Muridae | *Rattus norvegicus* | *Heterakis spumosa* | O | - | Proportional abundance 49%, prevalence 0.3% | South Africa | Potential (very low) | (163) |
| Rodentia: Muridae | *Rattus norvegicus* | *Mastophorus muris* | O | - | Proportional abundance 1.47%, prevalence >0.1% | South Africa | Potential (very low) | (163) |
| Rodentia: Muridae | *Rattus norvegicus* | *Protospirura sp.* | O | - | Presence only cited | South Africa | Potential (very low) | (163) |
| Rodentia: Muridae | *Rattus norvegicus* | *Strongyloides ratti* | C | - | Presence only cited | South Africa | Potential (very low) | (163) |
| Rodentia: Muridae | *Rattus norvegicus* | *Syphacia obvelata, Syphacia muris* | C;O | - | Presence only cited | South Africa | Potential (very low) | (163) |
| Rodentia: Muridae | *Rattus norvegicus* | *Trichuris sp.* | O | - | Presence only cited | South Africa | Potential (very low) | (163) |
| Rodentia: Muridae | *Rattus norvegicus* | *Trichosomoides crassicaud* | O | - | Prevalence >0.1% | South Africa | Potential (very low) | (163) |
| Rodentia: Muridae | *Rattus norvegicus* | *Inermicapsifer madagascariensis* | O | - | Presence only cited | South Africa | Potential (very low) | (163) |
| Rodentia: Muridae | *Rattus norvegicus* | *Hydatigera taeniaeformis* | O | - | Prevalence >0.1% | South Africa | Potential (very low) | (163) |
| Rodentia: Muridae | *Rattus norvegicus* | *Bartonella sp. (Bartonella elizabethae)* | V(F) | - | 12.5% prevalence of *Bartonella elizabethae* | United States | Potential (very low) | (164)  (165) |
| Rodentia: Muridae | *Rattus norvegicus* | *Brucella sp.* | C;O(F) | 0.73 | Not identified presumed below 12% | Australia | Potential (very low) | (137) |
| Rodentia: Muridae | *Rattus norvegicus* | *Calodium hepatica* | O | - | Presence only cited/87.9% prevalence | United States | Potential (very low) | (164) |
| Rodentia: Muridae | *Rattus norvegicus* | *Capillaria hepatica* | O | - | 25.9 – 36% *Capillaria hepatica* found in the liver/hepatic system. Also associated with lesions | Canada  Argentina  British Columbia | Potential (low) | (166)  (140)  (167) |
| Rodentia: Muridae | *Rattus norvegicus* | *Enterococcus* sp*.* | O | - | Presence only cited. Frequent bacterial species found in purulent lesions | Canada | Potential (very low) | (166) |
| Rodentia: Muridae | *Rattus norvegicus* | *Escherichia coli* | O(F) | 0.3 | Presence only cited. Most common bacterial species found in purulent lesions | Canada | Potential (very low) | (166) |
| Rodentia: Muridae | *Rattus norvegicus* | *Eucoleus* sp*.* | 0 | - | *Eucoleus* sp. in the upper gastrointestinal tract (164 of 399; 41%) as a capillarid nematode and associated with hyperkeratosis, mucosal hyperplasia, and submucosal inflammation in the forestomach | Canada | Potential (medium) | (166) |
|  |  | Hantavirus | A;C | - | Highest prevalence of the virus in rats (20%) than in other native rodents | United States | Potential (medium) | (168)  (169) |
| Rodentia: Muridae | *Rattus norvegicus* | Hepatitis E | O(F) | - | 77% of rats from Maryland, 90% from Hawaii, and 44% from Louisiana were seropositive for anti-HEV. Not considered a zoonotic strain | United States | Potential (low) | (165)  (170)  (164) |
| Rodentia: Muridae | *Rattus norvegicus* | *Hymenolepis* spp*. (Hymenolepis diminuta, Hymenolepis nana)* | O(F) | - | 7.4% with *Hymenolepis diminuta*; 33.3% with Hymenolepis nana | South Africa  Argentina  United States  British Columbia | Potential (low) | (163)  (140)  (164)  (167) |
| Rodentia: Muridae | *Rattus norvegicus* | *Leptospira* spp. *(Leptospira icterohaemorrhagiae [L. interrogans]; L. copenhageni)* | A;C;O(W) | 3.08 | 23-48% prevalence. 20% of Norway rats | Madagascar  United States  Puerto Rico  Chile  British Columbia | Potential (medium) | (141)  (164)  (142)  (143)  (167) |
| Rodentia: Muridae | *Rattus norvegicus* | *Monoliformis moniliformis* | O | - | 25.9% infected with *Monoliformis moniliformis* | Argentina | Potential (medium) | (140) |
| Rodentia: Muridae | *Rattus norvegicus* | *Nippostrongylus brasiliensis,* | C | - | 81.5% infected with *Nippostrongylus brasiliensis* | Argentina  Australia  South Africa | Potential (medium) | (163)  (137)  (140) |
| Rodentia: Muridae | *Rattus norvegicus* | *Hetereakis spumosa* | O | - | 88.9% infected with *Hetereakis spumosa* | Argentina | Potential (medium) | (140) |
| Rodentia: Muridae | *Rattus norvegicus* | *Gongylonema neoplasticum* | O | - | 3.7% infected with *Gongylonema neoplasticum* | Argentina | Potential (very low) | (140) |
| Rodentia: Muridae | *Rattus norvegicus* | *Nematospiroides dubius* | O | - | Presence only cited | British Columbia | Potential (very low) | (167) |
| Rodentia: Muridae | *Rattus norvegicus* | *Trichostrongylus* spp*.* | O(F) | - | Presence only cited | British Columbia | Potential (very low) | (167) |
| Rodentia: Muridae | *Rattus norvegicus* | *Coccidia* | O | - | Presence only cited | British Columbia | Potential (very low) | (167) |
| Rodentia: Muridae | *Rattus norvegicus* | *Entamoeba* | C;O(W) | - | Presence only cited | British Columbia | Potential (very low) | (167) |
| Rodentia: Muridae | *Rattus norvegicus* | *Salmonella panama* | O(F) | 0.2 | Presence only cited | British Columbia | Potential (very low) | (167) |
| Rodentia: Muridae | *Rattus norvegicus* | *Rickettsia typhi* | V(F) | - | 25.9% seroprevalence. Pathogen causes murine typhus | United States | Potential (very low) | (161)  (164)  (165) |
| Rodentia: Muridae | *Rattus norvegicus* | Seoul virus (Hantavirus) | A;C | - | 6.7% seroprevalence | United States | Potential (very low) | (164)  (165) |
| Rodentia: Muridae | *Rattus norvegicus* | *Staphylococcus aureus* | A;C | - | Presence only cited | Canada | Potential (very low) | (166) |
| Rodentia: Muridae | *Rattus norvegicus* | *Taenia taeniaeformis* | O | - | 22.2% with *Taenia taeniaeformis* | Argentina | Potential (medium) | (140) |
| Rodentia: Muridae | *Rattus norvegicus* | *Toxoplasma gondii* | O(F) | 2.11 | 3% of Norway rats | United States | Potential (very low) | (44) |
| Rodentia: Muridae | *Rattus norvegicus* | *Trichosomoides crassicauda* | O | - | *Trichosomoides crassicauda* in the urinary bladder (59 of 194; 30%) | Canada | Potential (medium) | (166) |
| Rodentia: Muridae | *Rattus norvegicus* | *Trypanosoma lewis* | V(F) | - | 68.8% of the sampled individuals. *Rattus* spp. Plays particular role in the continuous circulation of the pathogen | Nigeria | Potential (medium) | (145) |
| Rodentia: Muridae | *Rattus rattus* | *Angiostrongylus species* | O(F) | - | 84% of rats harboured helminths | Australia  United States | Potential (medium) | (171)  (172)  (173) |
| Rodentia: Muridae | *Rattus rattus* | *Toxocara cati* | O | - | 84% of rats harboured helminths | Australia | Potential (medium) | (171) |
| Rodentia: Muridae | *Rattus rattus* | *Ancylostoma braziliense* | C | - | 84% of rats harboured helminths | Australia | Potential (medium) | (171) |
| Rodentia: Muridae | *Rattus rattus* | *Toxocara cati* | O | - | 84% of rats harboured helminths | Australia | Potential (medium) | (171) |
| Rodentia: Muridae | *Rattus rattus* | *Hetereakis spumosa* | O | - | 14.3% with *Hetereakis spumosa* | Argentina  Australia  South Africa | Potential (low) | (140)  (163)  (171) |
| Rodentia: Muridae | *Rattus rattus* | *Hymenolepis spp.* | O(F) | - | 14.3% with *Hymenolepis diminuta*  3.6% *Hymenolepis nana* from South Africa | Argentina  Australia  South Africa | Potential (low) | (140)  (163)  (171) |
| Rodentia: Muridae | *Rattus rattus* | *Moniliformis moniliformis* | O | - | 7.1% with *Moniliformis moniliformis* | Argentina  South Africa | Potential (low) | (140)  (163) |
| Rodentia: Muridae | *Rattus rattus* | *Aspiculuris tetraptera* | O(F) | - | Presence only cited | South Africa | Potential (very low) | (163) |
| Rodentia: Muridae | *Rattus rattus* | *Eucoleus* sp. | O | - | Presence only cited | South Africa | Potential (very low) | (163) |
| Rodentia: Muridae | *Rattus rattus* | *Heterakis spumosa* | O | - | Presence only cited | South Africa | Potential (very low) | (163) |
| Rodentia: Muridae | *Rattus rattus* | *Mastophorus muris* | O | - | Presence only cited | South Africa | Potential (very low) | (163) |
| Rodentia: Muridae | *Rattus rattus* | *Strongyloides ratti* | C | - | Presence only cited | South Africa | Potential (very low) | (163) |
| Rodentia: Muridae | *Rattus rattus* | *Trichuris* sp. | O | - | Presence only cited | South Africa | Potential (very low) | (163) |
| Rodentia: Muridae | *Rattus rattus* | *Protospirura* sp. | O | - | Presence only cited. *Protospirura chabaudi* only species in the genus to be found in host species in African continent. Genus appears to be geographically restricted | South Africa | Potential (very low) | (163) |
| Rodentia: Muridae | *Rattus rattus* | *Trichosomoides crassicauda* | O | - | Presence only cited | South Africa | Potential (very low) | (163) |
| Rodentia: Muridae | *Rattus rattus* | *Inermicapsifer madagascariensis* | O | - | Presence only cited | South Africa | Potential (very low) | (163) |
| Rodentia: Muridae | *Rattus rattus* | *Hydatigera taeniaeformis* | O | - | Presence only cited. Metacestode presence confirms the role of synanthropic rodents to act as intermediate hosts. Cosmopolitan distribution and is a commonly found taeniid in domestic cats | South Africa | Potential (very low) | (163) |
| Rodentia: Muridae | *Rattus rattus* | *Babesia* sp. | V(T) | - | Presence only cited | Italy | Potential (very low) | (174) |
| Rodentia: Muridae | *Rattus rattus* | *Bartonella* spp. | V(F) | - | 1.3% (three of 228). In Chile, no data are available identifying fleas from synanthropic rodents as *Bartonella* spp. vectors | Chile  Uganda  Madagascar  Senegal | Potential (very low) | (175)  (176)  (177)  (136) |
| Rodentia: Muridae | *Rattus rattus* | *Borrelia burgorferi* | V(T) | - | Highest prevalence of the bacterium compared to native species (two out of six rats: 33.3%) | United States | Potential (medium) | (178) |
| Rodentia: Muridae | *Rattus rattus* | *Brucella* spp. | C; O(F) | 0.73 | Not identified presumed below 12%. Recently genotyped strain and a unique species. Large reservoir of pathogen in rodents in tropical Far North Queensland | Australia | Potential (very low) | (137) |
| Rodentia: Muridae | *Rattus rattus* | *Coxiella burnetii* | A; O(F) | - | Not identified presumed below 12% | Australia | Potential (very low) | (137) |
| Rodentia: Muridae | *Rattus rattus* | *Salmonella* spp. | O(F) | 0.2 | *Salmonella choleraesuis* ssp*. arizonae* (14.29%) | Australia | Potential (low) | (137) |
| Rodentia: Muridae | *Rattus rattus* | *Syphacia obvelata; Syphacia muris* | C;O | - | *Syphacia obvelata* (2.86%) | Australia  South Africa | Potential (very low) | (137)  (140) |
| Rodentia: Muridae | *Rattus rattus* | *Nippostrongylus brasiliensis* | C | - | *Nippostrongylus brasiliensis* (14.3 - 85.71%) | Australia  Argentina  South Africa | Potential (medium) | (137)  (140)  (163) |
| Rodentia: Muridae | *Rattus rattus* | Various parasites | - | - | *Laelaps* spp. (41.17%), *Polyplax* spp. (23.53%), *Hoplopleura* spp. (17.65%), *Ixodes holocyclus* (17.64%) and *Stephanocircus harrisoni* (5.88%) | Australia | Potential (medium) | (137) |
| Rodentia: Muridae | *Rattus rattus* | *Capillaria hepatica* | O | - | Histology revealed 15 (75%) of the rats sampled had a current or previous infection with *C. hepatica* | Diego Garcia - British Overseas Territories | Potential (medium) | (179) |
| Rodentia: Muridae | *Rattus rattus* | *Cryptosporidium* spp. | O(F,W) | 0.01 | 37.5% carried *Cryptosporidium spp.* | New Zealand | Potential (medium) | (42) |
| Rodentia: Muridae | *Rattus rattus* | *Giardia* spp. | O | 0.05 | 42.1% carried *Giardia spp.* | New Zealand | Potential (medium) | (42) |
| Rodentia: Muridae | *Rattus rattus* | Hantavirus | A;C | - | Presence only cited | Senegal | Potential (very low) | (180) |
| Rodentia: Muridae | *Rattus rattus* | *Hymenolepis* spp. | O(F) | - | 3.6% with *Hymenolepis nana*, 14.3% with *Hymenolepis diminuta* | South Africa  Argentina | Potential (low) | (163)  (140) |
| Rodentia: Muridae | *Rattus rattus* | *Leishmania* spp. | V(BF) | - | 17.5% infected | Italy  Senegal | Potential (low) | (174)  (180) |
| Rodentia: Muridae | *Rattus rattus* | *Leptospira* spp. | A;C;O(W) | 3.08 | 2.9 - 42.5% prevalence. Results indicate a high prevalence therefore considered a species of significant concern for public health | Madagascar  La Réunion Mauritius Seychelles South Africa  Swaziland Mozambique  Madagascar  Puerto Rico  Australia  Malayasia Borneo | Potential (medium) | (137)  (141)  (181)  (142)  (99)  (102)  (182) |
| Rodentia: Muridae | *Rattus rattus* | Mammarenavirus | A | - | Presence only cited | Senegal | Potential (very low) | (138) |
| Rodentia: Muridae | *Rattus rattus* | *Aspicularis tetraptera* | O(F) | - | 25% prevalence | Argentina | Potential (medium) | (140) |
| Rodentia: Muridae | *Rattus rattus* | Sin Nombre Virus (Hantavirus) | A;C | - | 1.7% of infected individuals, less than native rodents | United States | Potential (very low) | (183) |
| Rodentia: Muridae | *Rattus rattus* | *Taenia taeniaeformis* | O | - | Presence only cited | Argentina  Australia | Potential (very low) | (140)  (171) |
| Rodentia: Muridae | *Rattus rattus* | *Toxoplasma gondii* | O(F) | 2.11 | 38.2% prevalence. Particularly prevalent in Brazil, where up to 90% of humans have been exposed to the parasite. Strains are genetically and biologically different in Brazil compared to ones in North America and Europe | Italy  Brazil | Potential (medium) | (174)  (184) |
| Rodentia: Muridae | *Rattus rattus* | *Trypanosoma* sp. | O;V(TRI) | - | 25.2% - 71% prevalence | Nigeria  Benin  Senegal | Potential (medium) | (145)  (185)  (186)  (180) |
| Rodentia: Muridae | *Rattus rattus* | West Nile Virus | V(MOS) | 9.13 | 30% prevalence as an emerging mosquito-borne flavivirus | United States | Potential (medium) | (187) |
| Rodentia: Muridae | *Rattus tanezumi* | Oxyurids | - | - | 90% abundance of the helminth with 0.139% prevalence | South Africa | Potential (medium) | (163) |
| Rodentia: Muridae | *Rattus tanezumi* | *Mastophorus muris* | O | - | 0.45% abundance of the helminth with 0.02% prevalence | South Africa | Potential (very low) | (163) |
| Rodentia: Muridae | *Rattus tanezumi* | *Nippostrongylus brasiliensis* | C | - | 2.86% abundance of the helminth with 0.17% prevalence | South Africa | Potential (very low) | (163) |
| Rodentia: Muridae | *Rattus tanezumi* | *Hydatigera taeniaeformis* | O | - | 6.7% bundance of the helminth with 0.05% prevalence | South Africa | Potential (low) | (163) |

Table 7. Nematode parasites alien in Europe as causal agents for zoonotic diseases. C= contact transmission. O = oral transmission through food (F) or water (W). V = vector-borne transmission by mosquitoes (MOS). The criteria for actual or potential impact are outlined in Table 3.

| **Nematoda** | | | | | | | |
| --- | --- | --- | --- | --- | --- | --- | --- |
| Order: Family | Parasite | Transmission type | Health impacts | Evidence for introduction into Europe and links to human disease in Europe | Countries of impact | Actual or potential impact  (impact level) | Refs |
| Rhabditida: Onchocercidae | *Dirofilaria immities*  *Dirofilaria repens* | V(MOS) | Filarioid helminths with domestic and wild canids as main hosts and mosquitoes as vectors. Cause primarily pulmonary *(D. immitis*), ocular (*D. repens*), and subcutaneous (*D. repens*) dirofilariosis | Prior cases tended to be introduced but finding of D. repens in mosquito vectors now suggests the parasite has established in some parts of Europe.  Burden: 33 cases reported since 2012 | Austria | Actual  (low) | (188) |
| Secernentea: Ascarididae | *Baylisascaris procyonis* | O | Larvae of the Raccoon roundworm causes a severe or fatal neural larva migrans called baylisascariasis, in humans. Eggs excreted in raccoon feces are infective after 2–4 weeks and can remain so for years | Raccoon roundworm has been found in raccoon populations in Germany, Norway, Denmark and Poland (refs in Table 7).  Despite widespread transmission in its introduced host, only 1 human case has been notified in Europe | Germany  Denmark  Norway  Poland | Actual  (low) | (189) |
| Secernentea: Strongyloididae | *Strongyloides myopotami*  *Strongyloides stercoralis*  *Strongyloides procyonis* | C | Nutria/coypu roundworm where larvae burrow into skin causing nutria or swimmer’s itch.  Threadworm causing strongyloidiasis with respiratory, skin and/or digestive symptoms, complications in immune-compromised individuals.  Raccoon threadworm demonstrated can cause short-lived intestinal infection in healthy humans | Infection rates of *S. stercoralis* are increasing in Europe, nearing those in endemic areas. Domesticated small ruminants act as reservoir hosts and are thought to be enhancing establishment | central, south and east Europe | Actual  (medium) | (190) |

Table 8. Platyhelminthes parasites alien in Europe as causal agents for zoonotic diseases C= contact transmission. O = oral transmission through food (F) or water (W). V = vector-borne transmission by mosquitoes (MOS). The criteria for actual or potential impact are outlined in Table 3.

| **Platyhelminthes** | | | | | | | |
| --- | --- | --- | --- | --- | --- | --- | --- |
| Order: Family | Parasite | Transmission type | Health impacts | Evidence for introduction into Europe and links to human disease in Europe | Countries of impact | Actual or potential impact  (certainty) | Refs |
| Plagiorchiida: Heterophyidae | *Centrocestus formosanus* | O(F,W) | In humans, *C. formosanus* infections result in epigastric pain, indigestion, and diarrhea | Species spreading in freshwater fish trade (ornamental and research), sporadically reported in European fish populations. Human cases have not been recorded in Europe | Italy | Potential  (low) | (191) |
| Cestoda: Diphyllobothriidea | *Diphyllobothrium pacificum*  *D. dendriticum*  *D. nihonkaiens*  *D. balanopterae* | O(F) | Tapeworm causing diphyllobothriasis due to human consumption of raw or undercooked freshwater fish | Only *D. latum* is autochthonous in north-east Europe. Most of the cases in Europe are imported or caused by consumption of fish imported from endemic areas, particularly where fish have not been frozen. Very few cases reported overall. | Spain  Switzerland | Actual  (low) | (192) |

Table 9. Alien species with indirect impacts on zoonotic disease transmission. V = vector-borne transmission by tick (T) or mosquitoes (MOS). Case fatality is mean of case fatality rate reported by ECDC from 2012-2018 (where data are available). The criteria for actual or potential impact are outlined in Table 3.

| Order: Family | Name of Alien Species | Pathogen genus impacted | | | Evidence for role in zoonotic disease transmission | Countries of impact | Actual or potential impact  (certainty) | Refs |
| --- | --- | --- | --- | --- | --- | --- | --- | --- |
|  |  | Species within genus | Trans  mission | Case fatality rate (%) |  |  |  |  |
| Poales: Poaceae | *Phragmites australis* | West Nile Virus | V(MOS) | 9.13 | Management of the species increases disease risk by altering habitat quality for vectors (immatures) and avian host (roosts) | United States | Actual (low) | (193) |
| Poales: Typhaceae | *Typha* spp*.* | West Nile Virus | V(MOS) | 9.13 | IAS management increases disease risk by altering habitat quality for vectors (immatures) and avian host (roosts) | United States | Actual (low) | (193) |
| Peronosporales: Peronosporaceae | *Phytophthora ramorum* | *Borrelia burgdorferi* | V(T) | - | Introduced forest oomycete pathogen found in models to reduce nymphal infection prevalence through its impacts on forest structure, affecting hosts and tick abundance in coastal woodlands | United States | Actual (low) | (194) |
| Squamata: Pythonidae | *Python bivittatus* | Everglades virus, Venezuelan equine encephalitis complex | V(MOS) | 9.13 | Invasive python heavily predated the large mammals (i.e. deer, raccoons and opossums), inducing native mosquito *Culex cedecei* to feed more on hispid cotton rat *Sigmodon hispidus* (the primary reservoir host) and even on humans | United States | Actual (low) | (195) |
| Dipsacales: Caprifoliaceae | *Lonicera maackill* | *Ehrlichia* spp | V(T) | - | White-tailed deer, *Odocoileus virginianus*, the dominant host for the tick *Amblyomma americanum* carrying the bacteria *Ehrlichia* spp. (agents of human ehrlichiosis), used areas invaded by the Amur honeysuckle*, Lonicera maackii*, more frequently than uninvaded habitats | United States | Actual (low) | (196) |

# Appendix 1. Summary of studies in which pathogen / parasite prevalence and roles in transmission were compared between IAS (and other alien species) and native hosts

| **Class** | **Order: Family** | **Invasive Alien Species** | **Common name** | **Native Species** | **Common name** | | **Pathogen** | **Class of pathogen** | **Outcome** | **Country of study** | **Reference** |
| --- | --- | --- | --- | --- | --- | --- | --- | --- | --- | --- | --- |
| Insecta | Diptera: Culicidae | *Aedes aegypti* | Yellow fever mosquito | *Ae. triseriatus* | Eastern tree hole mosquito | | Eastern equine encephalitis | Virus | IAS ~ native | United States (Massachussets) | (10) |
| Insecta | Diptera: Culicidae | *Aedes albopictus* | Asian tiger mosquito | *Ae. triseriatus* | Eastern tree hole mosquito | | Eastern equine encephalitis | Virus | IAS ~ native | United States (Massachussets) | (10) |
| Insecta | Diptera: Culicidae | *Aedes japonicus japonicus* | Rock pool mosquito | *Ae. triseriatus* | Eastern tree hole mosquito | | Cache Valley virus | Virus | IAS ~ native | United States (Virginia) | (11) |
| Aves | Columbiformes: Columbidae | *Columba livia* | Pigeon | *Carpodacus mexicanus* | House finches | | St. Louis encephalitis (SLE) | Virus | IAS > native | United States (California) | (40) |
| Aves | Columbiformes: Columbidae | *Columba livia* | Pigeon | *Carpodacus mexicanus* | House finches | | Western equine encephalomyelitis (WEE) | Virus | IAS > native | United States (California) | (40) |
| Aves | Galliformes: Phasianidae | *Alectoris chukar* | chukar partridge | *Colinus virginianus* | Bobwhite quail | | *Chlamydia psittaci* | Bacteria | IAS ~ native | United States (Kentucky) | (35) |
| Aves | Passeriformes: Passeridae | *Passer domesticus* | House sparrow | *Carpodacus mexicanus* | House finches | | St. Louis encephalitis (SLE) | Virus | IAS ~ native | United States (California) | (40) |
| Aves | Passeriformes: Passeridae | *Passer domesticus* | House sparrow | *Carpodacus mexicanus* | House finches | | Western equine encephalomyelitis (WEE) | Virus | IAS ~ native | United States (California) | (40) |
| Mammalia | Artiodactyla: Suidae | *Sus scrofa* | feral swine | *Isoodon macrourus* | Northern brown bandicoot | | *Coxiella burnetii* | Bacteria | IAS ~ native | Australia | (57) |
| Mammalia | Carnivora: Canidae | *Canis lupus dingo* | dingo | *Isoodon macrourus* | Northern brown bandicoot | | *Coxiella burnetii* | Bacteria | IAS < native | Australia | (57) |
| Mammalia | Carnivora: Canidae | *Nyctereutes procyonoides* | Raccoon dog | *Vulpes vulpes* | Red fox | | *Alaria alata* | Intestinal helminths | IAS ~ native | Poland | (74) |
| Mammalia | Carnivora: Canidae | *Nyctereutes procyonoides* | Raccoon dog | *Vulpes vulpes* | Red fox | | *Alaria alata*; *Brachylaima tokudai*; *Cryptocotyle* spp.; *Dipylidium caninum*; *Echinococcus multilocularis*; *Mesocestoides* spp.; *Mesorchis denticulatus*; *Pygidiopsis summa*; *Taenia* spp.; *Toxocara canis*; *Trichinella* spp.; *Uncinaria stenocephala* | Platyhelminthes | Multiple pathogens and varies with pathogens | Denmark | (76) |
| Mammalia | Carnivora: Canidae | *Nyctereutes procyonoides* | Raccoon dog | *Vulpes vulpes* | Red fox | | *Alaria alata*; *Capillaria plica*; *Capillaria putorii*; *Crenosoma vulpis*; *Echinococcus multilocularis*; *Eucoleus aerophilus*; *Mesocestoides* spp.; *Taenia polyacantha*, *T. crassiceps*; *Toxocara canis*; *Trichinella* spp.; *Uncinaria stenocephala* | Nematoda; Platyhelminthes | Multiple pathogens and varies with pathogen | Lithuania | (77) |
| Mammalia | Carnivora: Canidae | *Nyctereutes procyonoides* | Raccoon dog | *Vulpes vulpes* | Red fox | | *Anaplasma phagocytophilum* | Bacteria | IAS > native | Germany | (79) |
| Mammalia | Carnivora: Canidae | *Nyctereutes procyonoides* | Raccoon dog | *Vulpes vulpes* | Red fox | | *Apophallus* spp. | Intestinal helminths | IAS > native | Poland | (74) |
| Mammalia | Carnivora: Canidae | *Nyctereutes procyonoides* | Raccoon dog | *Vulpes vulpes* | Red fox | | *Echinoccocus multilocularis* | Intestinal helminths | IAS < native | Poland | (74) |
| Mammalia | Carnivora: Canidae | *Nyctereutes procyonoides* | Raccoon dog | *Vulpes vulpes* | Red fox | | *Echinococcus multilocularis* | Tapeworm | IAS < native | Germany | (80) |
| Mammalia | Carnivora: Canidae | *Nyctereutes procyonoides* | Raccoon dog | *Vulpes vulpes* | Red fox | | *Echinococcus multilocularis* | Tapeworm | IAS < native | Estonia | (82) |
| Mammalia | Carnivora: Canidae | *Nyctereutes procyonoides* | Raccoon dog | *Vulpes vulpes* | Red fox | | *Francisella tularensis* | Bacteria | IAS < native | Germany | (84) |
| Mammalia | Carnivora: Canidae | *Nyctereutes procyonoides* | Raccoon dog | *Vulpes vulpes* | Red fox | | hookworms | Intestinal helminths | IAS > native | Poland | (74) |
| Mammalia | Carnivora: Canidae | *Nyctereutes procyonoides* | Raccoon dog | *Vulpes vulpes* | Red fox | | *Mesocestoides* spp. | Intestinal helminths | IAS < native | Poland | (74) |
| Mammalia | Carnivora: Canidae | *Nyctereutes procyonoides* | Raccoon dog | *Vulpes vulpes* | Red fox | | Rabies | Virus | IAS ~ native | Lithuania | (87) |
| Mammalia | Carnivora: Canidae | *Nyctereutes procyonoides* | Raccoon dog | *Vulpes vulpes* | Red fox | | Rabies | Virus | IAS < native | Lithuania | (88) |
| Mammalia | Carnivora: Canidae | *Nyctereutes procyonoides* | Raccoon dog | *Vulpes vulpes* | Red fox | | Rabies | Virus | IAS < native | Poland | (89) |
| Mammalia | Carnivora: Canidae | *Nyctereutes procyonoides* | Raccoon dog | *Martes martes* | Pine marten | | Rabies | Virus | IAS > native | Poland | (89) |
| Mammalia | Carnivora: Canidae | *Nyctereutes procyonoides* | Raccoon dog | *Vulpes vulpes* | Red fox | | Rabies | Virus | IAS > native | Poland | (90) |
| Mammalia | Carnivora: Canidae | *Nyctereutes procyonoides* | Raccoon dog | *Vulpes vulpes* | Red fox | | Rabies | Virus | IAS < native | Poland | (91) |
| Mammalia | Carnivora: Canidae | *Nyctereutes procyonoides* | Raccoon dog | *Vulpes vulpes* | Red fox | | rabies (Genotype 1, RABV) | Virus | IAS ~ native | Lithuania | (92) |
| Mammalia | Carnivora: Canidae | *Nyctereutes procyonoides* | Raccoon dog | *Vulpes vulpes* | Red fox | | *Taenia* spp. | Intestinal helminths | IAS < native | Poland | (74) |
| Mammalia | Carnivora: Canidae | *Nyctereutes procyonoides* | Raccoon dog | *Vulpes vulpes* | Red fox | | *Toxicara*/*Toxicaris* | Intestinal helminths | IAS < native | Poland | (74) |
| Mammalia | Carnivora: Canidae | *Nyctereutes procyonoides* | Raccoon dog | *Vulpes vulpes* | Red fox | | *Trichinella* | Roundworm | IAS ~ native | Lithuania, Lativa, Estonia | (93) |
| Mammalia | Carnivora: Canidae | *Vulpes vulpes* | Red fox | *Isoodon macrourus* | Northern brown bandicoot | | *Coxiella burnetii* | Bacteria | IAS > native | Australia | (57) |
| Mammalia | Carnivora: Felidae | *Felis catus* | Feral cat | *Isoodon macrourus* | Northern brown bandicoot | | *Coxiella burnetii* | Bacteria | IAS > native | Australia | (57) |
| Mammalia | Rodentia: Muridae | *Gerbillus nigeriae* | Nigerian gerbil | *Various* |  | | *Borrelia crocidurae* | Bacteria | IAS ~ native | Senegal | (139) |
| Mammalia | Rodentia: Muridae | *Mus musculus* | House mouse | *Various* |  | | *Hepatozoon canis* | Protozoa | IAS > native | Senegal | (139) |
| Mammalia | Rodentia: Muridae | *Mus musculus* | House mouse | *Various* |  | | *Leptospira* | Bacteria | IAS > native | Madagascar | (141) |
| Mammalia | Rodentia: Muridae | *Mus musculus* | House mouse | *Abrothrix olivaceus* | Olive grass mouse | | *Leptospira* spp; | Bacteria | IAS > native | Chile | (143) |
| Mammalia | Rodentia: Muridae | *Rattus norvegicus* | Norway rat | *Cricetomys gambianus* | Gambian pouch rat | | *Trypanosoma lewis* | Protozoa | IAS > native | Niger; Nigeria | (145) |
| Mammalia | Rodentia: Muridae | *Rattus norvegicus* | Norway rat | *Mastomys coucha* | Southern multimammate mouse | | *Aspiculuris tetraptera*; *Eucoleus* sp.; *Heterakis spumosa*; *Mastophorus muris*; *Nippostrongylus brasiliensis*; *Protospirura* sp.; *Strongyloides ratti*; *Syphacia obvelata*; *Syphacia muris*; *Trichuris* sp.; *Trichosomoides crassicauda*; *Hymenolepis diminuta*; *Hymenolepis nana*; *Inermicapsifer madagascariensis*; *Hydatigera taeniaeformis* | Nematoda; Platyhelminthes | IAS > native | South Africa | (163) |
| Mammalia | Rodentia: Muridae | *Rattus norvegicus* | Norway rat | *Various* |  | | Hantavirus | Virus | IAS > native | United States (Minnesota, Winsconsin) | (169) |
| Mammalia | Rodentia: Muridae | *Rattus norvegicus* | Norway rat | *Mastomys coucha* | Southern multimammate mouse | | *Hymenolepis diminuta*; *Hymenolepis nana* | Nematoda; Platyhelminthes | IAS > native | South Africa |  |
| Mammalia | Rodentia: Muridae | *Rattus norvegicus* | Norway rat | *Various* |  | | *Leptospira* | Bacteria | IAS > native | Madagascar | (141) |
| Mammalia | Rodentia: Muridae | *Rattus norvegicus* | Norway rat | *Abrothrix olivaceus* | Olive grass mouse | | *Leptospira* spp; | Bacteria | IAS > native | Chile | (143) |
| Mammalia | Rodentia: Muridae | *Rattus norvegicus* | Norway rat | *Cricetomys gambianus* | Gambian pouch rat | | *Trypanosoma lewis* | Protozoa | IAS > native | Niger; Nigeria | (145) |
| Mammalia | Rodentia: Muridae | *Rattus rattus* | Black rat | *Cricetomys gambianus* | Gambian pouch rat | | *Trypanosoma lewis* | Protozoa | IAS < native | Niger; Nigeria | (145) |
| Mammalia | Rodentia: Muridae | *Rattus rattus* | Black rat | *Mastomys coucha* | Southern multimammate mouse | | *Aspiculuris tetraptera*; *Eucoleus* sp.; *Heterakis spumosa*; *Mastophorus muris*; *Nippostrongylus brasiliensis*; *Protospirura* sp.; *Strongyloides ratti*; *Syphacia obvelata*; *Syphacia muris*; *Trichuris* sp.; *Trichosomoides crassicauda*; *Hymenolepis diminuta*; *Hymenolepis nana*; *Inermicapsifer madagascariensis*; *Hydatigera taeniaeformis*; *Moniliformis moniliformis* | Nematoda; Platyhelminthes; Acanthocephala | IAS > native | South Africa | (163) |
| Mammalia | Rodentia: Muridae | *Rattus rattus* | Black rat | *Arvicanthis niloticus* | | African grass rat | *Bartonella* spp. | Bacteria | IAS < native | Also Cricetomys gambianus | (176) |
| Mammalia | Rodentia: Muridae | *Rattus rattus* | Black rat | *Various* |  | | *Borrelia burgorferi* | Bacteria | IAS > native | United States (California) | (178) |
| Mammalia | Rodentia: Muridae | *Rattus rattus* | Black rat | *Mastomys coucha* | Southern multimammate mouse | | *Hymenolepis diminuta*; *Hymenolepis nana* | Nematoda; Platyhelminthes; Acanthocephala | IAS > native | South Africa | (163) |
| Mammalia | Rodentia: Muridae | *Rattus rattus* | Black rat | *Various* |  | | *Leishmania* spp. | Trypanosome | IAS > native | Senegal | (180) |
| Mammalia | Rodentia: Muridae | *Rattus rattus* | Black rat | *Various* |  | | *Leptospira* | Bacteria | IAS > native | Madagascar | (141) |
| Mammalia | Rodentia: Muridae | *Rattus rattus* | Black rat | *Various* |  | | *Leptospira* | Bacteria | IAS > native | La Réunion, Mauritius, Seychelles, South Africa, Swaziland, Mozambique, and Madagascar | (181) |
| Mammalia | Rodentia: Muridae | *Rattus rattus* | Black rat | *Sundamys muelleri* | Müller's giant Sunda rat | | *Leptospira* spp; | Bacteria | IAS > native | Malayian Borneo | (182) |
| Mammalia | Rodentia: Muridae | *Rattus rattus* | Black rat | *Various* |  | | Sin Nombre Virus | Virus | IAS < native | United States (Florida) | (183) |
| Mammalia | Rodentia: Muridae | *Rattus rattus* | Black rat | *Bubulcus ibis* | Cattle egret | | *Toxoplasma gondii* | Protozoa | IAS < native | Brazil (islands) | (184) |
| Mammalia | Rodentia: Muridae | *Rattus rattus* | Black rat | *Cricetomys gambianus* | Gambian pouch rat | | *Trypanosoma lewis* | Protozoa | IAS < native | Niger; Nigeria | (145) |
| Mammalia | Rodentia: Muridae | *Rattus rattus* | Black rat | *Various* |  | | *Trypanosoma lewisi* | Protozoa | IAS > native | Benin | (186) |
| Mammalia | Rodentia: Muridae | *Rattus rattus* | Black rat | *Various* |  | | *Trypanosoma lewisi* | Protozoa | IAS > native | Senegal | (180) |
| Mammalia | Rodentia: Muridae | *Rattus rattus* | Black rat | *Various* |  | | *Trypanosoma lewis* | Protozoa | IAS > native | Niger | (185) |
| Mammalia | Rodentia: Muridae | *Rattus rattus* | Black rat | *Sigmodon hispidus* | hispid cotton rat | | West Nile Virus | Virus | IAS ~ native | United States (Louisiana) | (187) |
| Mammalia | Rodentia: Muridae | *Rattus rattus* | Black rat | *Sciurus carolinensis* | Eastern grey squirrel | | West Nile Virus | Virus | IAS ~ native | United States (Louisiana) | (187) |
| Mammalia | Rodentia: Muridae | *Rattus tanezumi* | Tanezumi rat | *Mastomys coucha* | Southern multimammate mouse | | *Aspiculuris tetraptera*; *Eucoleus* sp.; *Heterakis spumosa*; *Mastophorus muris*; *Nippostrongylus brasiliensis*; *Protospirura* sp.; *Strongyloides ratti*; *Syphacia obvelata*; *Syphacia muris*; *Trichuri*s sp.; *Trichosomoides crassicauda*; *Hymenolepis diminuta*; *Hymenolepis nana*; *Inermicapsifer madagascariensis*; *Hydatigera taeniaeformis* | Nematoda; Platyhelminthes | IAS > native | South Africa | (163) |
| Mammalia | Rodentia: Muridae | *Rattus tanezumi* | Tanezumi rat | *Mastomys coucha* | Southern multimammate mouse | | *Hymenolepis diminuta*; Hymenolepis nana | Nematoda; Platyhelminthes | IAS > native | South Africa | (163) |
| Mammalia | Rodentia: Muridae | *Suncus murinus* | shrew | *Various* |  | | *Leptospira* | Bacteria | IAS > native | Madagascar | (141) |
| Mammalia | Rodentia: Sciuridae | *Tamias sibiricus barberi* | Siberian chipmunk | *Myodes glareolus* | Bank voles | | *Borrelia burgdorferi* species complex; | Bacteria | IAS > native | France | (159) |

Bibliography

1. Cleveland CA, Swanepoel L, Box EK, De Nicola A, Yabsley MJ. Rickettsia species in ticks collected from wild pigs (Sus scrofa) and Philippine deer (Rusa marianna) on Guam, Marianna Islands, USA. Acta Trop. 2019 Jun;194:89–92.

2. Duscher GG, Hodžić A, Hufnagl P, Wille-Piazzai W, Schötta A-M, Markowicz MA, et al. Adult Hyalomma marginatum tick positive for Rickettsia aeschlimannii in Austria, October 2018. Euro Surveill. 2018 Nov;23(48).

3. Yssouf A, Lagadec E, Bakari A, Foray C, Stachurski F, Cardinale E, et al. Colonization of Grande Comore Island by a lineage of Rhipicephalus appendiculatus ticks. Parasit Vectors. 2011 Mar 17;4:38.

4. Chamot E, Chatelanat P, Humair L, Aeschlimann A, Bowessidjaou J. [5 cases of Mediterranean boutonneuse fever in Switzerland]. Ann Parasitol Hum Comp. 1987;62(5):371–379.

5. Vezzani D, Eiras DF, Wisnivesky C. Dirofilariasis in Argentina: historical review and first report of Dirofilaria immitis in a natural mosquito population. Vet Parasitol. 2006 Mar 31;136(3-4):259–273.

6. Vezzani D, Mesplet M, Eiras DF, Fontanarrosa MF, Schnittger L. PCR detection of Dirofilaria immitis in Aedes aegypti and Culex pipiens from urban temperate Argentina. Parasitol Res. 2011 Apr;108(4):985–989.

7. Silaghi C, Beck R, Capelli G, Montarsi F, Mathis A. Development of Dirofilaria immitis and Dirofilaria repens in Aedes japonicus and Aedes geniculatus. Parasit Vectors. 2017 Feb 20;10(1):94.

8. Cancrini G, Frangipane di Regalbono A, Ricci I, Tessarin C, Gabrielli S, Pietrobelli M. Aedes albopictus is a natural vector of Dirofilaria immitis in Italy. Vet Parasitol. 2003 Dec;118(3-4):195–202.

9. Schaffner F, Medlock JM, Van Bortel W. Public health significance of invasive mosquitoes in Europe. Clin Microbiol Infect. 2013 Aug;19(8):685–692.

10. Komar N, Dohm DJ, Turell MJ, Spielman A. Eastern equine encephalitis virus in birds: relative competence of European starlings (Sturnus vulgaris). Am J Trop Med Hyg. 1999 Mar;60(3):387–391.

11. Yang F, Chan K, Marek PE, Armstrong PM, Liu P, Bova JE, et al. Cache Valley Virus in Aedes japonicus japonicus Mosquitoes, Appalachian Region, United States. Emerging Infect Dis. 2018;24(3):553–557.

12. Wagner S, Mathis A, Schönenberger AC, Becker S, Schmidt-Chanasit J, Silaghi C, et al. Vector competence of field populations of the mosquito species Aedes japonicus japonicus and Culex pipiens from Switzerland for two West Nile virus strains. Med Vet Entomol. 2018;32(1):121–124.

13. Schönenberger AC, Wagner S, Tuten HC, Schaffner F, Torgerson P, Furrer S, et al. Host preferences in host-seeking and blood-fed mosquitoes in Switzerland. Med Vet Entomol. 2016 Mar;30(1):39–52.

14. Schaffner F, Kaufmann C, Hegglin D, Mathis A. The invasive mosquito Aedes japonicus in Central Europe. Med Vet Entomol. 2009 Dec;23(4):448–451.

15. Martinet J-P, Ferté H, Failloux A-B, Schaffner F, Depaquit J. Mosquitoes of North-Western Europe as Potential Vectors of Arboviruses: A Review. Viruses. 2019 Nov 14;11(11).

16. Lounibos LP, Kramer LD. Invasiveness of Aedes aegypti and Aedes albopictus and Vectorial Capacity for Chikungunya Virus. J Infect Dis. 2016 Dec 15;214(suppl 5):S453–S458.

17. Paupy C, Kassa Kassa F, Caron M, Nkoghé D, Leroy EM. A chikungunya outbreak associated with the vector Aedes albopictus in remote villages of Gabon. Vector Borne Zoonotic Dis. 2012 Feb;12(2):167–169.

18. McTighe SP, Vaidyanathan R. Vector competence of Aedes albopictus from Virginia and Georgia for chikungunya virus isolated in the Comoros Islands, 2005. Vector Borne Zoonotic Dis. 2012 Oct;12(10):867–871.

19. Faraji A, Egizi A, Fonseca DM, Unlu I, Crepeau T, Healy SP, et al. Comparative host feeding patterns of the Asian tiger mosquito, Aedes albopictus, in urban and suburban Northeastern USA and implications for disease transmission. PLoS Negl Trop Dis. 2014 Aug 7;8(8):e3037.

20. Tsetsarkin KA, Chen R, Weaver SC. Interspecies transmission and chikungunya virus emergence. Curr Opin Virol. 2016 Feb;16:143–150.

21. Dennett JA, Bala A, Wuithiranyagool T, Randle Y, Sargent CB, Guzman H, et al. ASSOCIATIONS BETWEEN TWO MOSQUITO POPULATIONS AND WEST NILE VIRUS IN HARRIS COUNTY, TEXAS, 2003–06^1^. J Am Mosq Control Assoc. 2007 Sep;23(3):264–275.

22. Clements MF, Gidwani K, Kumar R, Hostomska J, Dinesh DS, Kumar V, et al. Measurement of recent exposure to Phlebotomus argentipes, the vector of Indian visceral Leishmaniasis, by using human antibody responses to sand fly saliva. Am J Trop Med Hyg. 2010 May;82(5):801–807.

23. Clements K, Giménez L, Jones DL, Wilson J, Malham SK. Epizoic barnacles act as pathogen reservoirs on shellfish beds. J Shellfish Res. 2013 Aug;32(2):533–538.

24. Majoros G, Fehér Z, Deli T, Földvári G. Establishment of Biomphalaria tenagophila snails in Europe. Emerging Infect Dis. 2008 Nov;14(11):1812–1814.

25. Stockdale-Walden HD, Slapcinsky J, Qvarnstrom Y, McIntosh A, Bishop HS, Rosseland B. Angiostrongylus cantonensis in Introduced Gastropods in Southern Florida. J Parasitol. 2015 Apr;101(2):156–159.

26. Heneberg P, Rojas A, Bizos J, Kocková L, Malá M, Rojas D. Focal Philophthalmus gralli infection possibly persists in Melanoides tuberculata over two years following the definitive hosts’ removal. Parasitol Int. 2014 Dec;63(6):802–807.

27. Literák I, Heneberg P, Sitko J, Wetzel EJ, Cardenas Callirgos JM, Čapek M, et al. Eye trematode infection in small passerines in Peru caused by Philophthalmus lucipetus, an agent with a zoonotic potential spread by an invasive freshwater snail. Parasitol Int. 2013 Aug;62(4):390–396.

28. Coelho P. Updated distribution and range expansion of the gastropod invader Melanoides tuberculata (Müller, 1774) in Brazilian waters. Bioinvasions Rec. 2018;7(4):405–409.

29. Li K, Clausen JH, Murrell KD, Liu L, Dalsgaard A. Risks for fishborne zoonotic trematodes in tilapia production systems in Guangdong province, China. Vet Parasitol. 2013 Nov 15;198(1-2):223–229.

30. Shamsi S, Turner A, Wassens S. Description and genetic characterization of a new Contracaecum larval type (Nematoda: Anisakidae) from Australia. J Helminthol. 2018 Mar;92(2):216–222.

31. Kuhn T, García-Màrquez J, Klimpel S. Adaptive radiation within marine anisakid nematodes: a zoogeographical modeling of cosmopolitan, zoonotic parasites. PLoS One. 2011 Dec 13;6(12):e28642.

32. Carlson JC, Franklin AB, Hyatt DR, Pettit SE, Linz GM. The role of starlings in the spread of Salmonella within concentrated animal feeding operations. J Appl Ecol. 2011 Apr;48(2):479–486.

33. Dickx V, Kalmar ID, Tavernier P, Vanrompay D. Prevalence and genotype distribution of Chlamydia psittaci in feral Canada geese (Branta canadensis) in Belgium. Vector Borne Zoonotic Dis. 2013 Jun;13(6):382–384.

34. Feare CJ, Sanders MF, Blasco R, Bishop JD. Canada goose (Branta canadensis) droppings as a potential source of pathogenic bacteria. J R Soc Promot Health. 1999 Sep;119(3):146–155.

35. Erbeck DH, Nunn SA. Chlamydiosis in Pen-Raised Bobwhite Quail (Colinus virginianus) and Chukar Partridge (Alectoris chukar) with High Mortality. Avian Dis. 1999 Oct;43(4):798.

36. Briceño C, Surot D, González-Acuña D, Martínez FJ, Fredes F. Parasitic survey on introduced monk parakeets (Myiopsitta monachus) in Santiago, Chile. Rev Bras Parasitol Vet. 2017 Jun;26(2):129–135.

37. Pisanu B, Laroucau K, Aaziz R, Vorimore F, Le Gros A, Chapuis J-L, et al. Chlamydia avium' ' detection from a ring-necked parakeet ( psittacula krameri ) in france. Journal of Exotic Pet Medicine. 2018 Feb;0(0):68–74.

38. Madani SA, Peighambari SM. PCR-based diagnosis, molecular characterization and detection of atypical strains of avian Chlamydia psittaci in companion and wild birds. Avian Pathol. 2013 Feb;42(1):38–44.

39. Mase M, Imada T, Sanada Y, Etoh M, Sanada N, Tsukamoto K, et al. Imported parakeets harbor H9N2 influenza A viruses that are genetically closely related to those transmitted to humans in Hong Kong. J Virol. 2001 Apr;75(7):3490–3494.

40. Gruwell JA, Fogarty CL, Bennett SG, Challet GL, Vanderpool KS, Jozan M, et al. Role of peridomestic birds in the transmission of St. Louis encephalitis virus in southern California. J Wildl Dis. 2000 Jan;36(1):13–34.

41. Nemeth NM, Bosco-Lauth AM, Sciulli RH, Gose RB, Nagata MT, Bowen RA. Serosurveillance for Japanese encephalitis and West Nile viruses in resident birds in Hawai’i. J Wildl Dis. 2010 Apr;46(2):659–664.

42. Chilvers BL, Cowan PE, Waddington DC, Kelly PJ, Brown TJ. The prevalence of infection of Giardia spp. and Cryptosporidium spp. in wild animals on farmland, southeastern North Island, New Zealand. Int J Environ Health Res. 1998 Mar;8(1):59–64.

43. Miles JA. The ecology of Whataroa virus, an alphavirus, in South Westland, New Zealand. J Hyg (Lond). 1973 Dec;71(4):701–713.

44. TIZARD IR, HARMESON J, LAI CH. PREVALENCE OF SERUM ANTIBODIES TO TOXOPLASMA-GONDII IN ONTARIO MAMMALS. Canadian Journal of Comparative Medicine-Revue Canadienne De Medecine Comparee. 1978;42(2):177–183.

45. Arrigo NC, Adams AP, Watts DM, Newman PC, Weaver SC. Cotton rats and house sparrows as hosts for North and South American strains of eastern equine encephalitis virus. Emerging Infect Dis. 2010 Sep;16(9):1373–1380.

46. Roy HE, Bacher S, Essl F, Adriaens T, Aldridge DC, Bishop JDD, et al. Developing a list of invasive alien species likely to threaten biodiversity and ecosystems in the European Union. Glob Change Biol. 2019;25(3):1032–1048.

47. Bigler WJ, Hoff GL, Hemmert WH, Tomas JA, Janowski HT. Trends of brucellosis in Florida. An epidemiologic review. Am J Epidemiol. 1977 Mar;105(3):245–251.

48. CLARK RK, JESSUP DA, HIRD DW, RUPPANNER R, MEYER ME. SEROLOGIC SURVEY OF CALIFORNIA WILD HOGS FOR ANTIBODIES AGAINST SELECTED ZOONOTIC DISEASE AGENTS. J Am Vet Med Assoc. 1983;183(11):1248–1251.

49. Musser JMB, Schwartz AL, Srinath I, Waldrup KA. Use of serology and bacterial culture to determine prevalence of Brucella spp. In feral Swine (sus scrofa) in proximity to a beef cattle herd positive for Brucella suis and Brucella abortus. J Wildl Dis. 2013 Apr;49(2):215–220.

50. Bevins SN, Pedersen K, Lutman MW, Gidlewski T, Deliberto TJ. Consequences Associated with the Recent Range Expansion of Nonnative Feral Swine. Bioscience. 2014 Apr 1;64(4):291–299.

51. Eales KM, Norton RE, Ketheesan N. Brucellosis in northern Australia. Am J Trop Med Hyg. 2010 Oct;83(4):876–878.

52. Ridoutt C, Lee A, Moloney B, Massey P, Charman N, Jordan D. Detection of brucellosis and leptospirosis in feral pigs in New South Wales. Aust Vet J. 2014 Sep;92(9):343–347.

53. Lama JK, Bachoon DS. Detection of Brucella suis, Campylobacter jejuni, and Escherichia coli Strains in Feral Pig (Sus scrofa) Communities of Georgia. Vector Borne Zoonotic Dis. 2018 Apr 26;18(7):350–355.

54. Carr AN, Milleson MP, Hernández FA, Merrill HR, Avery ML, Wisely SM. Wildlife Management Practices Associated with Pathogen Exposure in Non-Native Wild Pigs in Florida, U.S. Viruses. 2018 Dec 26;11(1).

55. Muller L, Poudyal N, Applegate RD, Yoest C. Control efforts and serologic survey of pseudorabies and brucellosis in wild pigs of Tennessee. Human-Wildlife Interactions. 2019;13(1):167–175.

56. Jay-Russell MT, Bates A, Harden L, Miller WG, Mandrell RE. Isolation of Campylobacter from feral swine (Sus scrofa) on the ranch associated with the 2006 Escherichia coli O157:H7 spinach outbreak investigation in California. Zoonoses Public Health. 2012 Aug;59(5):314–319.

57. Cooper A, Goullet M, Mitchell J, Ketheesan N, Govan B. Serological evidence of Coxiella burnetii exposure in native marsupials and introduced animals in Queensland, Australia. Epidemiol Infect. 2012 Jul;140(7):1304–1308.

58. Atwill ER, Sweitzer RA, Pereira MG, Gardner IA, Van Vuren D, Boyce WM. Prevalence of and associated risk factors for shedding Cryptosporidium parvum oocysts and Giardia cysts within feral pig populations in California. Appl Environ Microbiol. 1997 Oct;63(10):3946–3949.

59. Rodriguez-Rivera LD, Cummings KJ, McNeely I, Suchodolski JS, Scorza AV, Lappin MR, et al. Prevalence and diversity of cryptosporidium and giardia identified among feral pigs in texas. Vector Borne Zoonotic Dis. 2016 Oct 20;16(12):765–768.

60. Castellaw AH, Chenney EF, Varela-Stokes AS. Tick-borne disease agents in various wildlife from Mississippi. Vector Borne Zoonotic Dis. 2011 Apr;11(4):439–442.

61. Clavijo A, Nikooienejad A, Esfahani MS, Metz RP, Schwartz S, Atashpaz-Gargari E, et al. Identification and analysis of the first 2009 pandemic H1N1 influenza virus from U.S. feral swine. Zoonoses Public Health. 2013 Aug;60(5):327–335.

62. Hall JS, Minnis RB, Campbell TA, Barras S, Deyoung RW, Pabilonia K, et al. Influenza exposure in United States feral swine populations. J Wildl Dis. 2008 Apr;44(2):362–368.

63. Irwin MJ, Massey PD, Walker B, Durrheim DN. Feral pig hunting: a risk factor for human brucellosis in north-west NSW? N S W Public Health Bull. 2009 Dec;20(11-12):192–194.

64. Heise-Pavlov PM, Heise-Pavlov SR. Feral pigs in tropical lowland rainforest of northeastern Australia: ecology, zoonoses and management. Wildlife Biology. 2003;9:21–27.

65. Baroch JA, Gagnon CA, Lacouture S, Gottschalk M. Exposure of feral swine (Sus scrofa) in the United States to selected pathogens. Canadian Journal of Veterinary Research-Revue Canadienne De Recherche Veterinaire. 2015;79(1):74–78.

66. Brandão LNS, Rosa JMA, Kramer B, Sousa ATHI de, Trevisol IM, Silva VS, et al. Detection of Toxoplasma gondii infection in feral wild boars (Sus scrofa) through indirect hemagglutination and PCR. Cienc Rural. 2019 Mar 14;49(3).

67. Sandfoss M, DePerno C, Patton S, Flowers J, Kennedy-Stoskopf S. Prevalence of antibody to Toxoplasma gondii and Trichinella spp. in feral pigs (Sus scrofa) of eastern North Carolina. J Wildl Dis. 2011 Apr;47(2):338–343.

68. Dubey JP, Rollor EA, Smith K, Kwok OCH, Thulliez P. Low Seroprevalence of Toxoplasma gondii in Feral Pigs from a Remote Island Lacking Cats. J Parasitol. 1997 Oct;83(5):839.

69. Diderrich V, New JC, Noblet GP, Patton S. Serologic survey of Toxoplasma gondii antibodies in free-ranging wild hogs (Sus scrofa) from the Great Smoky Mountains National Park and from sites in South Carolina. J Eukaryot Microbiol. 1996 Oct;43(5):122S.

70. Hill DE, Dubey JP, Baroch JA, Swafford SR, Fournet VF, Hawkins-Cooper D, et al. Surveillance of feral swine for Trichinella spp. and Toxoplasma gondii in the USA and host-related factors associated with infection. Vet Parasitol. 2014 Oct 15;205(3-4):653–665.

71. Hidalgo A, Villanueva J, Becerra V, Soriano C, Melo A, Fonseca-Salamanca F. Trichinella spiralis Infecting Wild Boars in Southern Chile: Evidence of an Underrated Risk. Vector Borne Zoonotic Dis. 2019 Mar 5;19(8):625–629.

72. Nockler K. Prevalence and importance of Trichinella in Germany. Wiener Tierarztliche Monatsschrift. 2005;92(12):301–307.

73. Comeaux JM, Curtis-Robles R, Lewis BC, Cummings KJ, Mesenbrink BT, Leland BR, et al. Survey of Feral Swine ( Sus scrofa ) Infection with the Agent of Chagas Disease ( Trypanosoma cruzi ) in Texas, 2013-14. J Wildl Dis. 2016 Jul;52(3):627–630.

74. Karamon J, Samorek-Pieróg M, Moskwa B, Różycki M, Bilska-Zając E, Zdybel J, et al. Intestinal helminths of raccoon dogs (Nyctereutes procyonoides) and red foxes (Vulpes vulpes) from the Augustów Primeval Forest (north-eastern Poland). J Vet Res. 2016 Sep 1;60(3):273–277.

75. Laurimaa L, Süld K, Davison J, Moks E, Valdmann H, Saarma U. Alien species and their zoonotic parasites in native and introduced ranges: The raccoon dog example. Vet Parasitol. 2016 Mar 30;219:24–33.

76. Al-Sabi MNS, Chriél M, Jensen TH, Enemark HL. Endoparasites of the raccoon dog (Nyctereutes procyonoides) and the red fox (Vulpes vulpes) in Denmark 2009-2012 - A comparative study. Int J Parasitol Parasites Wildl. 2013 Dec;2:144–151.

77. Bružinskaitė-Schmidhalter R, Šarkūnas M, Malakauskas A, Mathis A, Torgerson PR, Deplazes P. Helminths of red foxes (Vulpes vulpes) and raccoon dogs (Nyctereutes' ' procyonoides) in Lithuania. Parasitology. 2012 Jan;139(1):120–127.

78. Duscher T, Hodžić A, Glawischnig W, Duscher GG. The raccoon dog (Nyctereutes procyonoides) and the raccoon (Procyon lotor)-their role and impact of maintaining and transmitting zoonotic diseases in Austria, Central Europe. Parasitol Res. 2017 Apr;116(4):1411–1416.

79. Härtwig V, von Loewenich FD, Schulze C, Straubinger RK, Daugschies A, Dyachenko V. Detection of Anaplasma phagocytophilum in red foxes (Vulpes vulpes) and raccoon dogs (Nyctereutes procyonoides) from Brandenburg, Germany. Ticks Tick Borne Dis. 2014 Apr;5(3):277–280.

80. Schwarz S, Sutor A, Staubach C, Mattis R, Tackmann K, Conraths FJ. Estimated prevalence of Echinococcus multilocularis in raccoon dogs Nyctereutes procyonoides in northern Brandenburg, Germany. Curr Zool. 2011 Oct 1;57(5):655–661.

81. Maas M, van den End S, van Roon A, Mulder J, Franssen F, Dam-Deisz C, et al. First findings of Trichinella spiralis and DNA of Echinococcus multilocularis in wild raccoon dogs in the Netherlands. Int J Parasitol Parasites Wildl. 2016 Dec;5(3):277–279.

82. Laurimaa L, Süld K, Moks E, Valdmann H, Umhang G, Knapp J, et al. First report of the zoonotic tapeworm Echinococcus multilocularis in raccoon dogs in Estonia, and comparisons with other countries in Europe. Vet Parasitol. 2015 Sep 15;212(3-4):200–205.

83. Schulze C, Heuner K, Myrtennäs K, Karlsson E, Jacob D, Kutzer P, et al. High and novel genetic diversity of Francisella tularensis in Germany and indication of environmental persistence. Epidemiol Infect. 2016 Jun 30;144(14):3025–3036.

84. Kuehn A, Schulze C, Kutzer P, Probst C, Hlinak A, Ochs A, et al. Tularaemia seroprevalence of captured and wild animals in Germany: the fox (Vulpes vulpes) as a biological indicator. Epidemiol Infect. 2013 Apr;141(4):833–840.

85. Schwarz S, Sutor A, Mattis R, Conraths FJ. [The raccoon roundworm (Baylisascaris procyonis)--no zoonotic risk for Brandenburg?]. Berl Munch Tierarztl Wochenschr. 2015 Feb;128(1-2):34–38.

86. Robardet E, Picard-Meyer E, Dobroštana M, Jaceviciene I, Mähar K, Muižniece Z, et al. Rabies in the baltic states: decoding a process of control and elimination. PLoS Negl Trop Dis. 2016 Feb 5;10(2):e0004432.

87. Maciulskis P, Lukauskas K, Sederevicius A, Kiudulas V, Pockevicius A. Epidemiology of enzootic rabies in Lithuania. Medycyna Weterynaryjna-Veterinary Medicine-Science and Practice. 2006;62(7):769–772.

88. Zienius D, Bagdonas J, Dranseika A. Epidemiological situation of rabies in Lithuania from 1990 to 2000. Vet Microbiol. 2003 May;93(2):91–100.

89. Lyczak A, Tomasiewicz K, Krawczuk G, Modrzewska R. Epizootic situation and risk of rabies exposure in Polish population in 2000, with special attention to Lublin province. Annals of Agricultural and Environmental Medicine. 2001;8(2):131–135.

90. Ostrowska JD, HermanowskaSzpakowicz T. Rabies and its prevention in people. Medycyna Weterynaryjna. 1997;53(3):144–147.

91. Lis H. Rabies of animals, in Poland. Medycyna Weterynaryjna. 1996;52(3):170–172.

92. Zienius D, Sajute K, Žilinskas H, Stankevicius A. Phylogenetic Analysis of the Rabies Virus N-coding Region in Lithuanian Rabies Isolates. Acta Vet Brno. 2009;78(2):273–280.

93. Malakauskas A, Paulauskas V, Järvis T, Keidans P, Eddi C, Kapel CMO. Molecular epidemiology of Trichinella spp. in three Baltic countries: Lithuania, Latvia, and Estonia. Parasitol Res. 2007 Mar;100(4):687–693.

94. Osten-Sacken N, Solarczyk P. Trichinella spiralis in road-killed raccoon dogs (Nyctereutes procyonoides) in western Poland. Ann Parasitol. 2016;62(1):77–79.

95. Deksne G, Segliņa Z, Jahundoviča I, Esīte Z, Bakasejevs E, Bagrade G, et al. High prevalence of Trichinella spp. in sylvatic carnivore mammals of Latvia. Vet Parasitol. 2016 Nov 15;231:118–123.

96. Mayer-Scholl A, Reckinger S, Schulze C, Nöckler K. Study on the occurrence of Trichinella spp. in raccoon dogs in Brandenburg, Germany. Vet Parasitol. 2016 Nov 15;231:102–105.

97. Cybulska A, Kornacka A, Moskwa B. The occurrence and muscle distribution of Trichinella britovi in raccoon dogs (Nyctereutes procyonoides) in wildlife in the Głęboki Bród Forest District, Poland. Int J Parasitol Parasites Wildl. 2019 Aug;9:149–153.

98. Laurimaa L, Davison J, Süld K, Plumer L, Oja R, Moks E, et al. First report of highly pathogenic Echinococcus granulosus genotype G1 in dogs in a European urban environment. Parasit Vectors. 2015 Mar 26;8:182.

99. Lagadec E, Gomard Y, Le Minter G, Cordonin C, Cardinale E, Ramasindrazana B, et al. Identification of Tenrec ecaudatus, a Wild Mammal Introduced to Mayotte Island, as a Reservoir of the Newly Identified Human Pathogenic Leptospira mayottensis. PLoS Negl Trop Dis. 2016 Aug 30;10(8):e0004933.

100. Kaewmongkol G, Kaewmongkol S, Fleming PA, Adams PJ, Ryan U, Irwin PJ, et al. Zoonotic Bartonella species in fleas and blood from red foxes in Australia. Vector Borne Zoonotic Dis. 2011 Dec;11(12):1549–1553.

101. Jenkins DJ, Fraser A, Bradshaw H, Craig PS. DETECTION OF *echinococcus granulosus* COPROANTIGENS IN AUSTRALIAN CANIDS WITH NATURAL OR EXPERIMENTAL INFECTION. Journal of Parasitology. 2000 Feb;86(1):140–145.

102. Dybing NA, Jacobson C, Irwin P, Algar D, Adams PJ. Leptospira Species in Feral Cats and Black Rats from Western Australia and Christmas Island. Vector Borne Zoonotic Dis. 2017 Mar 6;17(5):319–324.

103. Peterson DR, Cooney MK, Beasley RP. Prevalence of antibody to Toxoplasma among Alaskan natives: relation to exposure to the felidae. J Infect Dis. 1974 Dec;130(6):557–563.

104. Davis AA, Lepczyk CA, Haman KH, Morden CW, Crow SE, Jensen N, et al. *Toxoplasma gondii* Detection in Fecal Samples from Domestic Cats (*Felis catus* ) in Hawai‘i. Pac Sci. 2018 Oct;72(4):501–511.

105. Gholipour H, Busquets N, Fernández-Aguilar X, Sánchez A, Ribas MP, De Pedro G, et al. Influenza A Virus Surveillance in the Invasive American Mink (Neovison vison) from Freshwater Ecosystems, Northern Spain. Zoonoses Public Health. 2017;64(5):363–369.

106. Ramírez-Pizarro F, Silva-de la Fuente C, Hernández-Orellana C, López J, Madrid V, Fernández Í, et al. Zoonotic pathogens in the american mink in its southernmost distribution. Vector Borne Zoonotic Dis. 2019 Jul 17;19(12):908–914.

107. Kołodziej-Sobocińska M, Dvorožňáková E, Hurníková Z, Reiterová K, Zalewski A. Seroprevalence of Echinococcus spp. and Toxocara spp. in Invasive Non-native American Mink. Ecohealth. 2020 Mar;17(1):13–27.

108. Barros M, Sáenz L, Lapierre L, Nuñez C, Medina-Vogel G. High prevalence of pathogenic Leptospira in alien American mink (Neovison vison) in Patagonia. Rev chil hist nat. 2014 Dec;87(1):19.

109. Ribas MP, Almería S, Fernández-Aguilar X, De Pedro G, Lizarraga P, Alarcia-Alejos O, et al. Tracking Toxoplasma gondii in freshwater ecosystems: interaction with the invasive American mink (Neovison vison) in Spain. Parasitol Res. 2018 Jul;117(7):2275–2281.

110. Horimoto T, Maeda K, Murakami S, Kiso M, Iwatsuki-Horimoto K, Sashika M, et al. Highly pathogenic avian influenza virus infection in feral raccoons, Japan. Emerging Infect Dis. 2011 Apr;17(4):714–717.

111. Al-Sabi MNS, Chriél M, Hansen MS, Enemark HL. Baylisascaris procyonis in wild raccoons (Procyon lotor) in Denmark. Vet Parasitol Reg Stud Reports. 2015 Dec;1-2:55–58.

112. Rentería-Solís Z, Birka S, Schmäschke R, Król N, Obiegala A. First detection of Baylisascaris procyonis in wild raccoons (Procyon lotor) from Leipzig, Saxony, Eastern Germany. Parasitol Res. 2018 Oct;117(10):3289–3292.

113. Bauer C. Baylisascariosis (Baylisascaris procyonis) - a rare parasitic zoonosis in Europe. Berliner Und Munchener Tierarztliche Wochenschrift. 2011;124(11-12):465–472.

114. Xie Y, Zhou X, Li M, Liu T, Gu X, Wang T, et al. Zoonotic*Baylisascaris procyonis* Roundworms in Raccoons, China. Emerging Infect Dis. 2014 Dec;20(12):2170–2172.

115. Karamon J, Kochanowski M, Cencek T, Bartoszewicz M, Kusyk P. Gastrointestinal helminths of raccoons (Procyon lotor) in western Poland (Lubuskie province) - with particular regard to Baylisascaris procyonis. Bulletin of the Veterinary Institute in Pulawy. 2014 Dec 1;58(4):547–552.

116. Davidson RK, Øines Ø, Hamnes IS, Schulze JE. Illegal wildlife imports more than just animals--Baylisascaris procyonis in raccoons (Procyon lotor) in Norway. J Wildl Dis. 2013 Oct;49(4):986–990.

117. Sato H, Kamiya H, Furuoka H. Epidemiological aspects of the first outbreak of Baylisascaris procyonis larva migrans in rabbits in Japan. Journal of Veterinary Medical Science. 2003;65(4):453–457.

118. Küchle M, Knorr HL, Medenblik-Frysch S, Weber A, Bauer C, Naumann GO. Diffuse unilateral subacute neuroretinitis syndrome in a German most likely caused by the raccoon roundworm, Baylisascaris procyonis. Graefes Arch Clin Exp Ophthalmol. 1993;231(1):48–51.

119. Asakawa M, Matoba Y, Yamada D, Kamiyama T. Review of the parasitological state of feral raccoons captured in Nopporo National Park and its proximity, Hokkaido. Journal of Rakuno Gakuen University Natural Science. 2000;25(1):1–8.

120. Lee K, Iwata T, Nakadai A, Kato T, Hayama S, Taniguchi T, et al. Prevalence of Salmonella, Yersinia and Campylobacter spp. in feral raccoons (Procyon lotor) and masked palm civets (Paguma larvata) in Japan. Zoonoses Public Health. 2011 Sep;58(6):424–431.

121. Inoue K, Kabeya H, Fujita H, Makino T, Asano M, Inoue S, et al. Serological survey of five zoonoses, scrub typhus, Japanese spotted fever, tularemia, Lyme disease, and Q fever, in feral raccoons (Procyon lotor) in Japan. Vector Borne Zoonotic Dis. 2011 Jan;11(1):15–19.

122. Leśniańska K, Perec-Matysiak A, Hildebrand J, Buńkowska-Gawlik K, Piróg A, Popiołek M. Cryptosporidium spp. and Enterocytozoon bieneusi in introduced raccoons (Procyon lotor)-first evidence from Poland and Germany. Parasitol Res. 2016 Dec;115(12):4535–4541.

123. Nowakiewicz A, Zięba P, Ziółkowska G, Gnat S, Muszyńska M, Tomczuk K, et al. Free-Living Species of Carnivorous Mammals in Poland: Red Fox, Beech Marten, and Raccoon as a Potential Reservoir of Salmonella, Yersinia, Listeria spp. and Coagulase-Positive Staphylococcus. PLoS One. 2016 May 12;11(5):e0155533.

124. Stolte M, Odening K, Walter G. The raccoon as intermediate host of three Sarcocystis species in Europe. Journal of the Helminthological Society of Washington. 1996;63(1):145–149.

125. Sato H, Suzuki K, Osanai A, Kamiya H, Furuoka H. Identification and characterization of the threadworm, Strongyloides procyonis, from feral raccoons (Procyon lotor) in Japan. J Parasitol. 2006 Feb;92(1):63–68.

126. Baumeister S, Pohlmeyer K, Kuschfeldt S, Stoye M. On the prevalence of Echinococcus multilocularis and other metacestodes and cestodes in the muskrat (Ondatra zibethicus Link, 1795) in Lower Saxony. Deutsche Tierarztliche Wochenschrift. 1997;104(10):448–452.

127. Sinski E, Bednarska M, Bajer A. The role of wild rodents in ecology of cryptosporidiosis in Poland. Folia Parasitologica. 1998;45(2):173–174.

128. Boussinesq M, Bresson S, Liance M, Houin R. [A new natural intermediate host of Echinococcus multilocularis in France: the muskrat (Ondatra zibethicus L.)]. Ann Parasitol Hum Comp. 1986;61(4):431–434.

129. Umhang G, Richomme C, Boucher J-M, Guedon G, Boué F. Nutrias and muskrats as bioindicators for the presence of Echinococcus multilocularis in new endemic areas. Vet Parasitol. 2013 Oct 18;197(1-2):283–287.

130. Karanis P, Opiela K, Renoth S, Seitz HM. Possible contamination of surface waters with Giardia spp through muskrats. Zentralblatt Fur Bakteriologie-International Journal of Medical Microbiology Virology Parasitology and Infectious Diseases. 1996;284(2-3):302–306.

131. Vahlenkamp M, Müller T, Tackmann K, Löschner U, Schmitz H, Schreiber M. The muskrat (Ondatra zibethicus) as a new reservoir for puumala-like hantavirus strains in Europe. Virus Res. 1998 Oct;57(2):139–150.

132. Lataste-Dorolle C, Fiocre B. [Muskrats, Ondatra (Fiber zibethicus, Linnaeus), carriers in France of various leptospirian serotypes: isolation of the 1st French strain related to the Hebdomadis serogroup]. Bull Soc Pathol Exot Filiales. 1969;62(2):312–20.

133. Lim SR, Lee D-H, Park SY, Lee S, Kim HY, Lee M-S, et al. Wild Nutria (Myocastor coypus) Is a Potential Reservoir of Carbapenem-Resistant and Zoonotic Aeromonas spp. in Korea. Microorganisms. 2019 Jul 30;7(8).

134. Asakawa M, Sato M, Sone K, Tatsuzawa S, Oda S. Further helminthological survey on alien rodents, coypu (Myocastor coypus: Myocastoridae) in Aichi and Hyogo Prefectures, Japan. Journal of Rakuno Gakuen University Natural Science. 2009;33(2):291–292.

135. Nardoni S, Angelici MC, Mugnaini L, Mancianti F. Prevalence of Toxoplasma gondii infection in Myocastor coypus in a protected Italian wetland. Parasit Vectors. 2011 Dec 23;4:240.

136. Diagne C, Galan M, Tamisier L, d Ambrosio J, Dalecky A, Bâ K, et al. Ecological and sanitary impacts of bacterial communities associated to biological invasions in African commensal rodent communities. Sci Rep. 2017 Nov 3;7(1):14995.

137. Chakma S, Picard J, Duffy R, Constantinoiu C, Gummow B. A Survey of Zoonotic Pathogens Carried by Non-Indigenous Rodents at the Interface of the Wet Tropics of North Queensland, Australia. Transbound Emerg Dis. 2017 Feb;64(1):185–193.

138. Diagne CA, Charbonnel N, Henttonen H, Sironen T, Brouat C. Serological survey of zoonotic viruses in invasive and native commensal rodents in senegal, west africa. Vector Borne Zoonotic Dis. 2017 Sep 5;17(10):730–733.

139. Dahmana H, Granjon L, Diagne C, Davoust B, Fenollar F, Mediannikov O. Rodents as hosts of pathogens and related zoonotic disease risk. Pathogens. 2020 Mar 10;9(3).

140. Hancke D, Suárez OV. Helminth Diversity in Synanthropic Rodents from an Urban Ecosystem. Ecohealth. 2017 Apr 17;14(3):603–613.

141. Moseley M, Rahelinirina S, Rajerison M, Garin B, Piertney S, Telfer S. Mixed Leptospira Infections in a Diverse Reservoir Host Community, Madagascar, 2013-2015. Emerging Infect Dis. 2018;24(6):1138–1140.

142. Benavidez KM, Guerra T, Torres M, Rodriguez D, Veech JA, Hahn D, et al. The prevalence of Leptospira among invasive small mammals on Puerto Rican cattle farms. PLoS Negl Trop Dis. 2019 May 20;13(5):e0007236.

143. Correa JP, Bucarey SA, Cattan PE, Landaeta-Aqueveque C, Ramírez-Estrada J. Renal carriage of Leptospira species in rodents from Mediterranean Chile: The Norway rat (Rattus norvegicus) as a relevant host in agricultural lands. Acta Trop. 2017 Dec;176:105–108.

144. Galal L, Sarr A, Cuny T, Brouat C, Coulibaly F, Sembène M, et al. The introduction of new hosts with human trade shapes the extant distribution of Toxoplasma gondii lineages. PLoS Negl Trop Dis. 2019 Jul 11;13(7):e0007435.

145. Tatard C, Garba M, Gauthier P, Hima K, Artige E, Dossou DKHJ, et al. Rodent-borne Trypanosoma from cities and villages of Niger and Nigeria: A special role for the invasive genus Rattus? Acta Trop. 2017 Jul;171:151–158.

146. Nakamura M, Takahashi K, Taira K, Taira M, Ohno A, Sakugawa H, et al. Hepatitis E virus infection in wild mongooses of Okinawa, Japan: Demonstration of anti-HEV antibodies and a full-genome nucleotide sequence. Hepatol Res. 2006 Mar;34(3):137–140.

147. Takeuchi-Storm N, Al-Sabi MNS, Thamsborg SM, Enemark HL. *Alaria alata* Mesocercariae among Feral Cats and Badgers, Denmark. Emerging Infect Dis. 2015 Oct;20(10):1872–1874.

148. Adams WH, Emmons RW, Brooks JE. The changing ecology of murine (endemic) typhus in Southern California. Am J Trop Med Hyg. 1970 Mar;19(2):311–318.

149. Cuervo PF, Cataldo SD, Fantozzi MC, Deis E, Isenrath GD, Viberti G, et al. Liver fluke (Fasciola hepatica) naturally infecting introduced European brown hare (Lepus europaeus) in northern Patagonia: phenotype, prevalence and potential risk. Acta Parasitol. 2015 Sep;60(3):536–543.

150. Whitehouse CA, Keirstead N, Taylor J, Reinhardt JL, Beierschmitt A. Prevalence of hypermucoid Klebsiella pneumoniae among wild-caught and captive vervet monkeys (Chlorocebus aethiops sabaeus) on the island of St. Kitts. J Wildl Dis. 2010 Jul;46(3):971–976.

151. Jensen K, Alvarado-Ramy F, González-Martínez J, Kraiselburd E, Rullán J. B-virus and free-ranging macaques, Puerto Rico. Emerging Infect Dis. 2004 Mar;10(3):494–496.

152. Wisely SM, Sayler KA, Anderson CJ, Boyce CL, Klegarth AR, Johnson SA. Macacine Herpesvirus 1 Antibody Prevalence and DNA Shedding among Invasive Rhesus Macaques, Silver Springs State Park, Florida, USA. Emerging Infect Dis. 2018;24(2):345–351.

153. Basso W, Rütten M, Deplazes P, Grimm F. Generalized Taenia crassiceps cysticercosis in a chinchilla (Chinchilla lanigera). Vet Parasitol. 2014 Jan 17;199(1-2):116–120.

154. Shiokawa K, Llanes A, Hindoyan A, Cruz-Martinez L, Welcome S, Rajeev S. Peridomestic small Indian mongoose: An invasive species posing as potential zoonotic risk for leptospirosis in the Caribbean. Acta Trop. 2019 Feb;190:166–170.

155. Schlottau K, Hoffmann B, Homeier-Bachmann T, Fast C, Ulrich RG, Beer M, et al. Multiple detection of zoonotic variegated squirrel bornavirus 1 RNA in different squirrel species suggests a possible unknown origin for the virus. Arch Virol. 2017 Sep;162(9):2747–2754.

156. Millins C, Magierecka A, Gilbert L, Edoff A, Brereton A, Kilbride E, et al. An Invasive Mammal (the Gray Squirrel, Sciurus carolinensis) Commonly Hosts Diverse and Atypical Genotypes of the Zoonotic Pathogen Borrelia burgdorferi Sensu Lato. Appl Environ Microbiol. 2015 Jul;81(13):4236–4245.

157. Romeo C, Lecollinet S, Caballero J, Isla J, Luzzago C, Ferrari N, et al. Are tree squirrels involved in the circulation of flaviviruses in Italy? Transbound Emerg Dis. 2018 Oct;65(5):1372–1376.

158. Tappe D, Schmidt-Chanasit J, Rauch J, Allartz P, Herden C. Immunopathology of Fatal Human Variegated Squirrel Bornavirus 1 Encephalitis, Germany, 2011-2013. Emerging Infect Dis. 2019;25(6):1058–1065.

159. Jacquot M, Abrial D, Gasqui P, Bord S, Marsot M, Masseglia S, et al. Multiple independent transmission cycles of a tick-borne pathogen within a local host community. Sci Rep. 2016 Aug 8;6:31273.

160. Reeves WK, Durden LA, Dasch GA. A Spotted Fever Group *Rickettsia* from an Exotic Tick Species, *Amblyomma exornatum* (Acari: Ixodidae), in a Reptile Breeding Facility in the United States. ME. 2006 Sep 1;43(5):1099–1101.

161. Reeves WK, Easterbrook JD, Loftis AD, Glass GE. Serologic evidence for Rickettsia typhi and an ehrlichial agent in Norway rats from Baltimore, Maryland, USA. Vector Borne Zoonotic Dis. 2006;6(3):244–247.

162. Chikweto A, Bhaiyat MI, Macpherson CNL, Deallie C, Pinckney RD, Richards C, et al. Existence of Angiostrongylus cantonensis in rats (Rattus norvegicus) in Grenada, West Indies. Vet Parasitol. 2009 May 26;162(1-2):160–162.

163. Julius RS, Schwan EV, Chimimba CT. Helminth composition and prevalence of indigenous and invasive synanthropic murid rodents in urban areas of Gauteng Province, South Africa. J Helminthol. 2017 Sep 4;1–10.

164. Easterbrook JD, Kaplan JB, Vanasco NB, Reeves WK, Purcell RH, Kosoy MY, et al. A survey of zoonotic pathogens carried by Norway rats in Baltimore, Maryland, USA. Epidemiol Infect. 2007 Oct;135(7):1192–1199.

165. Smith HM, Reporter R, Rood MP, Linscott AJ, Mascola LM, Hogrefe W, et al. Prevalence study of antibody to ratborne pathogens and other agents among patients using a free clinic in downtown Los Angeles. J Infect Dis. 2002 Dec 1;186(11):1673–1676.

166. Rothenburger JL, Himsworth CG, La Perle KMD, Leighton FA, Nemeth NM, Treuting PM, et al. Pathology of wild Norway rats in Vancouver, Canada. J Vet Diagn Invest. 2019 Mar;31(2):184–199.

167. HARVEY DA, MACNEILL AC. A SURVEY OF ZOONOTIC DISEASES AND ARTHROPOD VECTORS ISOLATED FROM LIVE-TRAPPED NORWAY RATS (RATTUS-NORVEGICUS) IN THE MUNICIPALITY OF RICHMOND, BRITISH-COLUMBIA. Canadian Journal of Public Health-Revue Canadienne De Sante Publique. 1984;75(5):374–378.

168. YANAGIHARA R. HANTAVIRUS INFECTION IN THE UNITED-STATES - EPIZOOTIOLOGY AND EPIDEMIOLOGY. Reviews of Infectious Diseases. 1990;12(3):449–457.

169. Burek KA, Rossi CA, Leduc JW, Yuill TM. Serologic and virologic evidence of a Prospect Hill-like hantavirus in Wisconsin and Minnesota. Am J Trop Med Hyg. 1994 Sep;51(3):286–294.

170. Kabrane-Lazizi Y, Fine JB, Elm J, Glass GE, Higa H, Diwan A, et al. Evidence for widespread infection of wild rats with hepatitis E virus in the United States. Am J Trop Med Hyg. 1999 Aug;61(2):331–335.

171. Dybing NA, Jacobson C, Irwin P, Algar D, Adams PJ. Challenging the dogma of the “Island Syndrome”: a study of helminth parasites of feral cats and black rats on Christmas Island. Australasian Journal of Environmental Management. 2018 Jan 2;25(1):99–118.

172. Stokes VL, Spratt DM, Banks PB, Pech RP, Williams RL. Occurrence of Angiostrongylus species (Nematoda) in populations of Rattus rattus and Rattus fuscipes in coastal forests of south-eastern Australia. Aust J Zool. 2007;55(3):177.

173. Wallace GD, Rosen L. Studies on eosinophilic meningitis. i. observations on the geographic distribution of angiostrongylus cantonensis in the pacific area and its prevalence in wild rats. Am J Epidemiol. 1965 Jan;81:52–62.

174. Fratini S, Natali C, Zanet S, Iannucci A, Capizzi D, Sinibaldi I, et al. Assessment of rodenticide resistance, eradication units, and pathogen prevalence in black rat populations from a Mediterranean biodiversity hotspot (Pontine Archipelago). Biol Invasions. 2020 Apr;22(4):1379–1395.

175. Moreno Salas L, Espinoza-Carniglia M, Lizama Schmeisser N, Torres LG, Silva-de la Fuente MC, Lareschi M, et al. Fleas of black rats (Rattus rattus) as reservoir host of Bartonella spp. in Chile. PeerJ. 2019 Aug 1;7:e7371.

176. Billeter SA, Borchert JN, Atiku LA, Mpanga JT, Gage KL, Kosoy MY. Bartonella species in invasive rats and indigenous rodents from Uganda. Vector Borne Zoonotic Dis. 2014 Mar;14(3):182–188.

177. Brook CE, Bai Y, Yu EO, Ranaivoson HC, Shin H, Dobson AP, et al. Elucidating transmission dynamics and host-parasite-vector relationships for rodent-borne Bartonella spp. in Madagascar. Epidemics. 2017 Mar 16;20:56–66.

178. Peavy CA, Lane RS, Kleinjan JE. Role of small mammals in the ecology of Borrelia burgdorferi in a peri-urban park in north coastal California. Exp Appl Acarol. 1997 Aug;21(8):569–584.

179. Berentsen AR, Vogt S, Guzman AN, Vice DS, Pitt WC, Shiels AB, et al. Capillaria hepatica infection in black rats (Rattus rattus) on Diego Garcia, British Indian Ocean Territory. J Vet Diagn Invest. 2015 Mar;27(2):241–244.

180. Cassan C, Diagne CA, Tatard C, Gauthier P, Dalecky A, Bâ K, et al. Leishmania major and Trypanosoma lewisi infection in invasive and native rodents in Senegal. PLoS Negl Trop Dis. 2018 Jun 29;12(6):e0006615.

181. Dietrich M, Gomard Y, Lagadec E, Ramasindrazana B, Le Minter G, Guernier V, et al. Biogeography of Leptospira in wild animal communities inhabiting the insular ecosystem of the western Indian Ocean islands and neighboring Africa. Emerg Microbes Infect. 2018 Apr 4;7(1):57.

182. Blasdell KR, Morand S, Perera D, Firth C. Association of rodent-borne Leptospira spp. with urban environments in Malaysian Borneo. PLoS Negl Trop Dis. 2019 Feb 27;13(2):e0007141.

183. Glass GE, Livingstone W, Mills JN, Hlady WG, Fine JB, Biggler W, et al. Black Creek Canal Virus infection in Sigmodon hispidus in southern Florida. Am J Trop Med Hyg. 1998 Nov;59(5):699–703.

184. Costa DGC, Marvulo MFV, Silva JSA, Santana SC, Magalhães FJR, Filho CDFL, et al. Seroprevalence of Toxoplasma gondii in domestic and wild animals from the Fernando de Noronha, Brazil. J Parasitol. 2012 Jun;98(3):679–680.

185. Dobigny G, Poirier P, Hima K, Cabaret O, Gauthier P, Tatard C, et al. Molecular survey of rodent-borne Trypanosoma in Niger with special emphasis on T. lewisi imported by invasive black rats. Acta Trop. 2011 Mar;117(3):183–188.

186. Dobigny G, Gauthier P, Houéménou G, Dossou HJ, Badou S, Etougbétché J, et al. Spatio-temporal survey of small mammal-borne Trypanosoma lewisi in Cotonou, Benin, and the potential risk of human infection. Infect Genet Evol. 2019 Jul 22;75:103967.

187. Dietrich G, Montenieri JA, Panella NA, Langevin S, Lasater SE, Klenk K, et al. Serologic evidence of west nile virus infection in free-ranging mammals, Slidell, Louisiana, 2002. Vector Borne Zoonotic Dis. 2005;5(3):288–292.

188. Fuehrer H-P, Auer H, Leschnik M, Silbermayr K, Duscher G, Joachim A. Dirofilaria in Humans, Dogs, and Vectors in Austria (1978-2014)-From Imported Pathogens to the Endemicity of Dirofilaria repens. PLoS Negl Trop Dis. 2016 May 19;10(5):e0004547.

189. Dunbar M, Lu S, Chin B, Huh L, Dobson S, Al-Rawahi GN, et al. Baylisascariasis: A young boy with neural larva migrans due to the emerging raccoon round worm. Ann Clin Transl Neurol. 2019;6(2):397–400.

190. Puthiyakunnon S, Boddu S, Li Y, Zhou X, Wang C, Li J, et al. Strongyloidiasis--an insight into its global prevalence and management. PLoS Negl Trop Dis. 2014 Aug 14;8(8):e3018.

191. Pace A, Dipineto L, Aceto S, Censullo MC, Valoroso MC, Varriale L, et al. Diagnosis of Centrocestus formosanus Infection in Zebrafish (Danio rerio) in Italy: A Window to a New Globalization-Derived Invasive Microorganism. Animals (Basel). 2020 Mar 9;10(3).

192. Kuchta R, Esteban J-G, Brabec J, Scholz T. Misidentification of diphyllobothrium species related to global fish trade, Europe. Emerging Infect Dis. 2014 Nov;20(11):1955–1957.

193. Mackay AJ, Muturi EJ, Ward MP, Allan BF. Cascade of ecological consequences for West Nile virus transmission when aquatic macrophytes invade stormwater habitats. Ecol Appl. 2016 Jan;26(1):219–232.

194. Swei A, Briggs CJ, Lane RS, Ostfeld RS. Impacts of an introduced forest pathogen on the risk of Lyme disease in California. Vector Borne Zoonotic Dis. 2012 Aug;12(8):623–632.

195. Hoyer IJ, Blosser EM, Acevedo C, Thompson AC, Reeves LE, Burkett-Cadena ND. Mammal decline, linked to invasive Burmese python, shifts host use of vector mosquito towards reservoir hosts of a zoonotic disease. Biol Lett. 2017 Oct;13(10).
