# Supplementary material for: The role of invasive alien species in the emergence and spread of zoonoses
Source: Biol Invasions. 2022 Dec 20;25(4):1249–64. doi: 10.1007/s10530-022-02978-1 (PMC9763809; doi:10.1007/s10530-022-02978-1)
Supplement: Supplementary file 2 — Supplementary file2 (DOCX 56 kb) [file 10530_2022_2978_MOESM2_ESM.docx]

# Supplementary Information 2

Table of parasites encompassed in the IAS-parasite interactions, with organism, type of transmission and broad host associations and human health impact in Europe. A = aerosol transmission. C= contact transmission. O = oral transmission through food (F) or (W). V = vector-borne transmission by either flea (F), tick (T), mite (MI), lice (LI), biting flies (BF), *Triatminae* or kissing bugs (Tri) or mosquitoes (MOS). Annual Cases is the mean number of cases reported each year to ECDC from 2012-2018 and case fatality rate is mean of case fatality rate per year reported by ECDC from 2012-2018 (where data are available).

| **Organism** | **Class** | **Transmission** | **Hosts** | **Cases** | **Case fatality (%)** | **References** |
| --- | --- | --- | --- | --- | --- | --- |
| *Aeromonas* spp. | Bacteria | O(W) | Fish, Mollusca, Amphibians, Reptiles, Shrimp, Livestock | - | - | 1 |
| *Alaria alata* | Trematode | O(F) | Red fox, wild boar, mustelids | - | - | 2 |
| *Anaplasma phagocytophilum* | Bacteria | V(T) | Ruminants, Rodents, Horse, Rodents, Canines, Felines | - | - | 3 |
| *Anasakis simplex* | Nematode | O(F) | Fish, Cephalopods, Sea mammals and birds | - | - | 4 |
| *Ancylostoma* spp. | Nematode | C | Felines, Canines | - | - | 5 |
| *Angiostrongylus* spp. | Nematode | O(F) | Rodents, Mollusca, Felines, Canids, Mustelids | - | - | 6,7 |
| Anoplocephalidae | Cestode | O | Wide host range including mites, reptiles, birds and mammals. | - | - | 8 |
| *Apophallus* spp. | Trematode | O(F) | Molluscs and Fish | - | - | 9 |
| Ascaridinae | Nematode | O(F)(W) | Wide host range including Cats, birds, pigs | - | - | 10 |
| *Aspicularis tetraptera* | Nematode | O(F) | Rodents | - | - | 11 |
| *Babesia* spp. | Apicomplexa | V(T) | Dogs, Rodents | - | - | 12,13,14 |
| Bartonella spp. | Bacteria | V(F) | Rodents, Felines, Ruminants, Canines | - | - | 15 |
| *Baylisascaris procyonis* | Nematode | O | Raccoon | - | - | 16 |
| *Borrelia burgdorferi* | Bacteria | V(T) | Rodents | - | - | 17 |
| *Borrelia* spp. | Bacteria | V(T, LI) | Broad host range | - | - | 18,19 |
| *Brucella* spp. | Bacteria | C;O(F) | Ruminants, Canines, Pigs, Rodents | 388 | 0.73 | 20 |
| B-virus | Virus | C | Primates | - | - | 21 |
| Cache Valley Virus | Virus | V(MOS) | Broad host range including goats, sheep and cattle | - | - | 22 |
| *Calodium hepatica* | Nematode | O | Primarily rodent but wide host range | - | - | 23 |
| *Campylobacte*r spp. | Bacteria | O(F) | Poultry, Ruminants, Pigs, Wild birds | 233533 | 0.13 | 24 |
| *Rickettsia senegalensis* | Bacteria | V(F) |  | - | - | 25 |
| *Capillaria hepatica* | Nematode | O | Rodents, Carnivores | - | - | 26 |
| *Centrocestus formosanus* | Trematode | O(W)(F) | Wide range of hosts including birds, mammals, fish, Mollusca | - | - | 27 |
| Chikungunya virus | Virus | V(MOS) | Primates | - | - | 28 |
| *Chlamydia* spp. | Bacteria | A;C | Broad host range including Birds, Poultry and Livestock | - | - | 29,30 |
| *Clonorchis sinensis* | Trematode | O(W)(F) | Fish, Crustaceans, Mollusca | - | - | 31 |
| Coccidia | Apicomplexa | O | Poultry, Livestock, Domestic animals | - | - | 32 |
| Coliforms | Bacteria | C | Broad host range | - | - | 33 |
| *Contracaecum bancrofti* | Nematode | O(W)(F) | Fish | - | - | 34 |
| *Coxiella burnetii* | Bacteria | A;O(F) | Ruminants | 810 | 0.69 | 35 |
| *Cryptosporidium* spp. | Apicomplexa | O(W)(F) | Broad host range including mammals and birds | 11043 | 0.01 | 36 |
| Dengue | Virus | V(MOS) | Primates | 16* | NA | 37 |
| *Dicrocoelium dendriticum* | Trematode | O | Ants, molluscs, ruminants | - | - | 38 |
| *Diphyllobothrium* spp. | Cestode | O(F) | Fish, Copepods, Carnivores | - | - | 39 |
| *Diplogonoporus balaenopterae* | Cestode | O(F) | Fish, Copepods, Carnivores | - | - | 40 |
| *Dirofilaria* spp. | Nematode | V(MOS) | Canines, Felines | - | - | 41,42 |
| Eastern equine encephalitis virus | Virus | V(MOS) | Horses, Birds | - | - | 43 |
| *Echinococcus* spp. | Cestode | O | Ruminants, Canines | 815 | 0.64 | 44 |
| *Echinostomatid* spp. | Trematode | O(F) | Birds, Mammals, Reptiles, Fish | - | - | 45 |
| *Ehrlichia* spp. | Bacteria | V(T) | Deer, Canines, Rodents | - | - | 46 |
| *Entamoeba* | Amoeba | C;O(W) | - | - | - | 47 |
| *Enterococcus* spp. | Bacteria | O | Wide host range | - | - | 48 |
| *Enterocytozoon bieneusi* | Fungus | O(W) | Broad host range | - | - | 49 |
| *Escherichia coli* | Bacteria | O(F) | Broad host range including livestock | 6311 | 0.30 | 50 |
| *Eucoleus* spp. | Nematode | O | Canine and Feline | - | - | 51 |
| Everglades virus | Virus | V(MOS) | Rodents | - | - | 52 |
| *Fasciola hepatica* | Trematode | O(W)(F) | Bovine, Mollusca | - | - | 53 |
| *Francisella tularensis* | Bacteria | V(T,BF,MOS) | Rodents, Squirrels, Rabbits | 585 | 3.05 | 54 |
| *Giardia* spp. | Diplomonad | O | Wide range of hosts Including livestock and felines and canines | 18242 | 0.05 | 55 |
| *Gongylonema neoplasticum* | Nematode | O | Rodents | - | - | 56 |
| Hantavirus | Virus | A;C | Small mammals | 1236* | NA | 57 |
| *Haplorchis pumilio* | Trematode | O(F) | Fish, Canines, Felines, Pigs | - | - | 58 |
| Hepatitis E | Virus | O(F) | Broad host range including pigs, poultry, ruminants, rodents | - | - | 59 |
| *Hepatozoon canis* | Apicomplexa | V(T) | Dogs, hematophagous arthropods | - | - | 60 |
| *Heterakis spumosa* | Nematode | O | Rodents | - | - | 61 |
| Heterophyidae | Trematode | O(F) | Wide host range fish eating mammals and birds | - | - | 62 |
| *Hydatigera taeniaeformis* | Cestode | O | Felines, rodents | - | - | 63 |
| *Hymenolepis* spp. | Cestode | O(F) | Rodents, Beetles | - | - | 64,65 |
| *Inermicapsifer madagascariensis* | Cestode | O | Rodents, Beetles | - | - | 66 |
| Influenza A | Virus | A;C | Wide host range including Birds, Pigs | - | - | 67 |
| Japanese encephalitis virus | Virus | V(MOS) | Wild Birds, Pigs, Horses, Bovine | - | - | 68 |
| *Klebsiella pneumoniae* | Bacteria | A | Birds | - | - | 69 |
| *Leishmania* spp. | Trypanosome | V(BF) | Canines, Rodents, | - | - | 70 |
| *Leptospira* spp. | Bacteria | A;C;O(W) | Broad host range including rodents, livestock, pets | 725 | 3.08 | 71,72 |
| *Listeria* spp | Bacteria | O(F) | Wide range of host including livestock, canines and rodents | 2801 | 15.91 | 73 |
| Macacine herpesvirus 1 (McHV-1) | Virus | C | Primates | - | - | 74 |
| Mammarenavirus | Virus | A | Rodents | - | - | 75 |
| *Mastophorus muris* | Nematode | O | Rodents | - | - | 76 |
| *Mesocestoides* spp. | Nematode | O | Canines, felines, birds, mites, rodents | - | - | 77 |
| *Molineus* spp. | Nematode | O | Felines, Canines | - | - | 78 |
| *Moniliformis moniliformis* | Acanthocephalan | O | Rodents, Felines, Canines, Cockroach | - | - | 79 |
| *Mycobacterium avium* | Bacteria | A | Broad host range including livestock, rodents and birds | - | - | 80 |
| *Nematospiroides dubius* | Nematode | O | Rodents | - | - | 81 |
| *Nippostrongylus brasiliensis* | Nematode | C | Rodents | - | - | 82 |
| *Orientia tsutsugamushi* | Bacteria | V(MI) | Mite | - | - | 83 |
| *Paragonimus westermani* | Platyhelminth | O(F) | Pigs, Crustaceans, Molluscs | - | - | 84 |
| *Philophthalmus gralli* | Platyhelminth | O(W) | Birds, Molluscs | - | - | 85 |
| *Protospirura* spp. | Nematode | O | Rodents, Canines, Insects | - | - | 86,87 |
| Pseudorabies virus | Virus | A;O | Swine, Ruminants, Carnivores and Rodents | - | - | 88 |
| *Pterygodermatites* spp. | Nematode | O | Rodents, Canines, Felines | - | - | 89,90 |
| Rabies virus | Virus | C | Canines, Raccoon, Bats | 1.14 | 100.00 | 91 |
| *Rickettsia aeschlimannii* | Bacteria | V(T) | Ticks | - | - | 92 |
| *Rickettsia conorii* | Bacteria | V(T) | Ticks | - | - | 93 |
| *Rickettsia japonica* | Bacteria | V(T) | Rodents | - | - | 94 |
| *Rickettsia typhi* | Bacteria | V(F) | Rodents | - | - | 95 |
| *Salmonella enterica* | Bacteria | O(F) | Birds, Livestock | - | - | 96 |
| *Salmonella panama* | Bacteria | O(F) | Reptiles, Livestock | - | - | 97 |
| *Salmonella* spp. | Bacteria | O(F) | Wide range of hosts Including Livestock, Reptiles and Poultry | 90703 | 0.20 | 98 |
| *Schistosoma mansoni* | Trematode | O(W) | Molluscs | - | - | 99 |
| Seoul Hantavirus | Virus | A;C | Small mammals | - | - | 100 |
| Sin Nombre Virus (Hantavirus) | Virus | A;C | Small mammals | 1236* | NA | 101 |
| St. Louis encephalitis virus (SLEV) | Virus | V(MOS) | Birds | - | - | 102 |
| *Staphylococcus* spp. | Bacteria | A;C | Poultry, Cattle and Canines | - | - | 103 |
| *Streptococcus suis* | Bacteria | O(F) | Pigs, Poultry, Ruminants, Felines, Canines | - | - | 104 |
| *Strongyloides* spp. | Nematode | C | Rodents, Raccoon | - | - | 105,106,107 |
| *Syphacia* spp. | Nematode | O | Rodents | - | - | 108,109 |
| *Taenia* spp. | Cestode | O(F) | Broad host range including Felines, Bovine, Pigs, Canines and Rodents | - | - | 111,112,113 |
| *Theileria parva* | Apicomplexa | V(T) | Buffalo, Cattle | - | - | 114 |
| *Toxocara* spp. | Nematode | O | Felines, Canines, Cats, Rodents, birds, worms, molluscs | - | - | 115,116 |
| *Toxoplasma* spp. | Apicomplexa | O(F) | Wide host range including felines, birds and mammals | 199 | 2.11 | 117 |
| *Trichinella* spp. | Nematode | O(F) | Broad host range including birds, mammals and reptiles | 189 | 0.42 | 118 |
| *Trichosomoides crassicauda* | Nematode | O | Rodents | - | - | 119 |
| *Trichostrongylus* spp. | Nematode | O(F) | Livestock, Rabbits | - | - | 120 |
| *Trichuris* spp. | Nematode | O | Wide host range including domestic animals | - | - | 121 |
| *Trypanosoma* | Apicomplexa | O;V(GEN,TRI,FL) | Broad host range including rodents, domestic animals and livestock | - | - | 122,123,124 |
| *Uncinaria stenocephala* | Nematodes | O | Canines | - | - | 125 |
| Usutu virus (USUV) | Virus | V(MOS) | Birds | - | - | 126 |
| Variegated squirrel bornavirus 1 (VSBV-1) | Virus | A | Squirrels | - | - | 127 |
| West Nile Virus | Virus | V(MOS) | Birds | 383 | 9.13 | 128 |
| Western equine encephalomyelitis virus (WEEV) | Virus | V(MOS) | Birds, Horses | - | - | 129 |
| Whataroa virus | Virus | V(MOS) | Birds | - | - | 130 |
| *Yersinia pestis* | Bacteria | A;V(FL) | Broad incidental host range but mainly maintained in rodents | - | - | 131 |
| *Yersinia* spp. | Bacteria | A;O(W)(F);V(F) | Broad host range including domestic animals and rodents | 6780 | 0.07 | 132 |
| Zika virus | Virus | V(MOS) | Primates | - | - | 133 |

|  | References |
| --- | --- |
| 1 | Igbinosa, I.H., Igumbor, E.U., Aghdasi, F., Tom, M. and Okoh, A.I., 2012. Emerging Aeromonas species infections and their significance in public health. The Scientific World Journal, 2012. |
| 2 | Tăbăran, F., Sándor, A.D., Marinov, M., Cătoi, C. and Mihalca, A.D., 2013. Alaria alata infection in European mink. Emerging Infectious Diseases, 19(9), p.1547. |
| 3 | Rikihisa, Y., 2011. Mechanisms of obligatory intracellular infection with Anaplasma phagocytophilum. Clinical microbiology reviews, 24(3), pp.469-489. |
| 4 | Audicana, M.T. and Kennedy, M.W., 2008. Anisakis simplex: from obscure infectious worm to inducer of immune hypersensitivity. Clinical microbiology reviews, 21(2), pp.360-379. |
| 5 | Scharf, C., 2017. Dermoscopy Findings in Infectious and Parasitic Diseases. In The Microbiology of Skin, Soft Tissue, Bone and Joint Infections (pp. 303-313). Academic Press. |
| 6 | Barratt, J., Chan, D., Sandaradura, I., Malik, R., Spielman, D., Lee, R., Marriott, D., Harkness, J., Ellis, J. and Stark, D., 2016. Angiostrongylus cantonensis: a review of its distribution, molecular biology and clinical significance as a human pathogen. Parasitology, 143(9), pp.1087-1118. |
| 7 | Valente, R., Robles, M.D.R., Navone, G.T. and Diaz, J.I., 2018. Angiostrongylus spp. in the Americas: geographical and chronological distribution of definitive hosts versus disease reports. Memórias do Instituto Oswaldo Cruz, 113(3), pp.143-152. |
| 8 | Denegri, G., Bernadina, W. and Perez-Serrano, J., 1998. Anoplocephalid cestodes of veterinary and medical signiﬁcance: a review. Folia Parasitol (Praha), 45(1), pp.1-8. |
| 9 | Tyutin, A.V. and Izvekova, G.I., 2013. Infection of mollusks and fish by the trematode Apophallus muehlingi (Jagerskiold, 1898) and its interrelations with intermediate hosts. Inland water biology, 6(1), pp.52-56. |
| 10 | Mota, K.C.P., Grama, D.F., Fava, N.M.N., Úngari, L.P., Faria, E.S.M. and Cury, M.C., 2018. Distribution and risk factors of Ascarididae and other geohelminths in the soil of Uberlandia, Minas Gerais, Brazil. Revista do Instituto de Medicina Tropical de São Paulo, 60. |
| 11 | Derothe, J.M., Loubes, C., Orth, A., Renaud, F. and Moulia, C., 1997. Comparison between patterns of pinworm infection (Aspiculuris tetraptera) in wild and laboratory strains of mice, Mus musculus. International journal for parasitology, 27(6), pp.645-651. |
| 12 | Yabsley, M.J. and Shock, B.C., 2013. Natural history of zoonotic Babesia: role of wildlife reservoirs. International Journal for Parasitology: Parasites and Wildlife, 2, pp.18-31. |
| 13 | Hu, C.M., Humair, P.F., Wallich, R. and Gern, L., 1997. Apodemus sp. rodents, reservoir hosts for Borrelia afzelii in an endemic area in Switzerland. Zentralblatt für Bakteriologie, 285(4), pp.558-564. |
| 14 | Alho, A.M., Pita, J., Amaro, A., Amaro, F., Schnyder, M., Grimm, F., Custódio, A.C., Cardoso, L., Deplazes, P. and de Carvalho, L.M., 2016. Seroprevalence of vector-borne pathogens and molecular detection of Borrelia afzelii in military dogs from Portugal. Parasites & vectors, 9(1), pp.1-6. |
| 15 | Vayssier‐Taussat, M., Le Rhun, D., Bonnet, S. and Cotté, V., 2009. Insights in Bartonella host specificity. Annals of the New York Academy of Sciences, 1166(1), pp.127-132. |
| 16 | Sorvillo, F., Ash, L.R., Berlin, O.G.W., Yatabe, J., Degiorgio, C. and Morse, S.A., 2002. Baylisascaris procyonis: an emerging helminthic zoonosis. Emerging infectious diseases, 8(4), p.355. |
| 17 | Hyde, J.A., 2017. Borrelia burgdorferi keeps moving and carries on: a review of borrelial dissemination and invasion. Frontiers in immunology, 8, p.114. |
| 19 | Fryxell, R.T.T., Steelman, C.D., Szalanski, A.L., Kvamme, K.L., Billingsley, P.M. and Williamson, P.C., 2012. Survey of Borreliae in ticks, canines, and white-tailed deer from Arkansas, USA. Parasites & vectors, 5(1), p.139. |
| 20 | de Figueiredo, P., Ficht, T.A., Rice-Ficht, A., Rossetti, C.A. and Adams, L.G., 2015. Pathogenesis and immunobiology of brucellosis: review of Brucella–Host Interactions. The American journal of pathology, 185(6), pp.1505-1517. |
| 21 | Jainkittivong, A. and Langlais, R.P., 1998. Herpes B virus infection. Oral Surgery, Oral Medicine, Oral Pathology, Oral Radiology, and Endodontology, 85(4), pp.399-403. |
| 22 | Waddell, L., Pachal, N., Mascarenhas, M., Greig, J., Harding, S., Young, I. and Wilhelm, B., 2019. Cache Valley virus: A scoping review of the global evidence. Zoonoses and public health, 66(7), pp.739-758. |
| 23 | Fuehrer, H.P., 2014. An overview of the host spectrum and distribution of Calodium hepaticum (syn. Capillaria hepatica): part 2—Mammalia (excluding Muroidea). Parasitology Research, 113(2), pp.641-651. |
| 24 | Facciolà, A., Riso, R., Avventuroso, E., Visalli, G., Delia, S.A. and Laganà, P., 2017. Campylobacter: from microbiology to prevention. Journal of preventive medicine and hygiene, 58(2), p.E79. |
| 25 | Mediannikov, O., Aubadie-Ladrix, M. and Raoult, D., 2015. Candidatus ‘Rickettsia senegalensis’ in cat fleas in Senegal. New Microbes and New Infections, 3, pp.24-28. |
| 26 | Misdraji, J., 2010. Liver and bile duct infections. Diagnostic Pathology of Infectious Disease, p.255. |
| 27 | Pinto HA, Gonçalves NQ, López-Hernandez D, Pulido-Murillo EA, Melo AL. 2018. The life cycle of a zoonotic parasite reassessed: Experimental infection of Melanoides tuberculata (Mollusca: Thiaridae) with Centrocestus formosanus (Trematoda: Heterophyidae). PLoS ONE 13(4): e0194161. |
| 28 | Staples, J.E., Breiman, R.F. and Powers, A.M., 2009. Chikungunya fever: an epidemiological review of a re-emerging infectious disease. Clinical infectious diseases, 49(6), pp.942-948. |
| 29 | Knittler, M.R., Berndt, A., Böcker, S., Dutow, P., Hänel, F., Heuer, D., Kägebein, D., Klos, A., Koch, S., Liebler-Tenorio, E. and Ostermann, C., 2014. Chlamydia psittaci: new insights into genomic diversity, clinical pathology, host–pathogen interaction and anti-bacterial immunity. International Journal of Medical Microbiology, 304(7), pp.877-893. |
| 30 | Bachmann, N.L., Polkinghorne, A. and Timms, P., 2014. Chlamydia genomics: providing novel insights into chlamydial biology. Trends in microbiology, 22(8), pp.464-472. |
| 31 | Tang, Z.L., Huang, Y. and Yu, X.B., 2016. Current status and perspectives of Clonorchis sinensis and clonorchiasis: epidemiology, pathogenesis, omics, prevention and control. Infectious diseases of poverty, 5(1), p.71. |
| 32 | Dubey, J.P. ed., 2019. Coccidiosis in Livestock, Poultry, Companion Animals, and Humans. CRC Press. |
| 34 | Shamsi, S., Stoddart, A., Smales, L. and Wassens, S., 2019. Occurrence of Contracaecum bancrofti larvae in fish in the Murray–Darling Basin. Journal of helminthology, 93(5), pp.574-579. |
| 35 | Glaser, C. and Christie, L., 2010. Rickettsial and ehrlichial infections. In Handbook of Clinical Neurology (Vol. 96, pp. 143-158). Elsevier. |
| 36 | Fayer, R. and Ungar, B.L., 1986. Cryptosporidium spp. and cryptosporidiosis. Microbiological reviews, 50(4), p.458. |
| 37 | Valentine, M.J., Murdock, C.C. and Kelly, P.J., 2019. Sylvatic cycles of arboviruses in non-human primates. Parasites & vectors, 12(1), p.463. |
| 38 | Ekstam, B., Johansson, B., Dinnétz, P. and Ellström, P., 2011. Predicting risk habitats for the transmission of the small liver fluke, Dicrocoelium dendriticum to grazing ruminants. Geospatial health, pp.125-131. |
| 39 | Scholz, T. and Kuchta, R., 2016. Fish-borne, zoonotic cestodes (Diphyllobothrium and relatives) in cold climates: a never-ending story of neglected and (re)-emergent parasites. Food and waterborne parasitology, 4, pp.23-38. |
| 41 | McCall, J.W., Varloud, M., Hodgkins, E., Mansour, A., DiCosty, U., McCall, S., Carmichael, J., Carson, B. and Carter, J., 2017. Shifting the paradigm in Dirofilaria immitis prevention: blocking transmission from mosquitoes to dogs using repellents/insecticides and macrocyclic lactone prevention as part of a multimodal approach. Parasites & vectors, 10(2), p.525. |
| 42 | McCall, J.W., Varloud, M., Hodgkins, E., Mansour, A., DiCosty, U., McCall, S., Carmichael, J., Carson, B. and Carter, J., 2017. Shifting the paradigm in Dirofilaria immitis prevention: blocking transmission from mosquitoes to dogs using repellents/insecticides and macrocyclic lactone prevention as part of a multimodal approach. Parasites & vectors, 10(2), p.525. |
| 43 | Morens, D.M., Folkers, G.K. and Fauci, A.S., 2019. Eastern Equine Encephalitis Virus—Another Emergent Arbovirus in the United States. New England Journal of Medicine, 381(21), pp.1989-1992. |
| 44 | McManus, D.P., Zhang, W., Li, J. and Bartley, P.B., 2003. Echinococcosis. The Lancet, 362(9392), pp.1295-1304. |
| 45 | Toledo, R. and Fried, B., 2014. Helminth-Trematode: Echinostoma. |
| 47 | Skappak, C., Akierman, S., Belga, S., Novak, K., Chadee, K., Urbanski, S.J., Church, D. and Beck, P.L., 2014. Invasive amoebiasis: a review of Entamoeba infections highlighted with case reports. Canadian Journal of Gastroenterology and Hepatology, 28. |
| 48 | Torres, C., Alonso, C.A., Ruiz‐Ripa, L., León‐Sampedro, R., Del Campo, R. and Coque, T.M., 2018. Antimicrobial Resistance in Enterococcus spp. of animal origin. Antimicrobial Resistance in Bacteria from Livestock and Companion Animals, pp.185-227. |
| 49 | Guo, Y., Alderisio, K.A., Yang, W., Cama, V., Feng, Y. and Xiao, L., 2014. Host specificity and source of Enterocytozoon bieneusi genotypes in a drinking source watershed. Applied and Environmental Microbiology, 80(1), pp.218-225. |
| 50 | Wasteson, Y., 2002. Zoonotic Escherichia coli. Acta Veterinaria Scandinavica, 43(1), pp.1-6. |
| 51 | Elhamiani Khatat, S., Rosenberg, D., Benchekroun, G. and Polack, B., 2016. Lungworm Eucoleus aerophilus (Capillaria aerophila) infection in a feline immunodeficiency virus-positive cat in France. Journal of Feline Medicine and Surgery Open Reports, 2(1), p.2055116916651649. |
| 52 | Coffey, L.L., Crawford, C., Dee, J., Miller, R., Freier, J. and Weaver, S.C., 2006. Serologic evidence of widespread Everglades virus activity in dogs, Florida. Emerging infectious diseases, 12(12), p.1873. |
| 53 | Vázquez, A.A., de Vargas, M., Alba, A., Sanchez, J., Alda, P., Sabourin, E., Vittecoq, M., Alarcón-Elbal, P.M., Pointier, J.P. and Hurtrez-Boussès, S., 2019. Reviewing Fasciola hepatica transmission in the West Indies and novel perceptions from experimental infections of sympatric vs. allopatric snail/fluke combinations. Veterinary parasitology, 275, p.108955. |
| 54 | Ellis, J., Oyston, P. C., Green, M., & Titball, R. W. 2002. Tularemia. Clinical microbiology reviews, 15(4), 631–646. |
| 55 | Heyworth, M.F., 2016. Giardia duodenalis genetic assemblages and hosts. Parasite, 23. |
| 56 | da Costa Cordeiro, H., de Vasconcelos Melo, F.T., Giese, E.G. and Santos, J.N.D., 2018. Gongylonema parasites of rodents: A key to species and new data on Gongylonema neoplasticum. The Journal of parasitology, 104(1), pp.51-59. |
| 57 | Ramsden, C., Holmes, E.C. and Charleston, M.A., 2009. Hantavirus evolution in relation to its rodent and insectivore hosts: no evidence for codivergence. Molecular biology and evolution, 26(1), pp.143-153. |
| 58 | Anh, N.T.L., Phuong, N.T., Johansen, M.V., Murrell, K.D., Van, P.T., Dalsgaard, A., Thu, L.T. and Thamsborg, S.M., 2009. Prevalence and risks for fishborne zoonotic trematode infections in domestic animals in a highly endemic area of North Vietnam. Acta Tropica, 112(2), pp.198-203. |
| 59 | Van der Poel, W.H., 2014. Food and environmental routes of Hepatitis E virus transmission. Current opinion in virology, 4, pp.91-96. |
| 60 | Baneth, G.A.D., Samish, M., Alekseev, E., Aroch, I. and Shkap, V., 2001. Transmission of Hepatozoon canis to dogs by naturally-fed or percutaneously-injected Rhipicephalus sanguineus ticks. Journal of Parasitology, 87(3), pp.606-611. |
| 61 | Šnábel, V., Utsuki, D., Kato, T., Sunaga, F., Ooi, H.K., Gambetta, B. and Taira, K., 2014. Molecular identification of Heterakis spumosa obtained from brown rats (Rattus norvegicus) in Japan and its infectivity in experimental mice. Parasitology research, 113(9), pp.3449-3455. |
| 62 | Taraschewski, H., 1985. Transmission experiments on the host specificity ofHeterophyes species in 16 potential definitive hosts. Zeitschrift für Parasitenkunde, 71(4), pp.505-518. |
| 63 | Premaalatha, B., Chandrawathani, P., Tan, P.S., Tharshini, J., Jamnah, O., Ramlan, M. and NOR, I.S., 2016. Taenia taeniaeformis in wild rats. |
| 64 | Bogitsh, B.J., Carter, C.E. and Oeltmann, T.N., 2018. Human parasitology. Academic Press. |
| 65 | Thompson, R.C.A., 2015. Neglected zoonotic helminths: Hymenolepis nana, Echinococcus canadensis and Ancylostoma ceylanicum. Clinical Microbiology and Infection, 21(5), pp.426-432. |
| 66 | Khemiri, H., Jrijer, J., Neifar, L. and Nouira, S., 2017. A survey study on the helminth parasites of two wild jirds, Meriones shawi and M. libycus (Rodentia: Gerbillinae), in Tunisian desert areas. The European Zoological Journal, 84(1), pp.303-310. |
| 67 | Yoon, S.W., Webby, R.J. and Webster, R.G., 2014. Evolution and ecology of influenza A viruses. In Influenza Pathogenesis and Control-Volume I (pp. 359-375). Springer, Cham. |
| 68 | Mansfield, K.L., Hernández-Triana, L.M., Banyard, A.C., Fooks, A.R. and Johnson, N., 2017. Japanese encephalitis virus infection, diagnosis and control in domestic animals. Veterinary microbiology, 201, pp.85-92. |
| 69 | Davies, Y.M., Cunha, M.P.V., Oliveira, M.G.X., Oliveira, M.C.V., Philadelpho, N., Romero, D.C., Milanelo, L., Guimarães, M.B., Ferreira, A.J.P., Moreno, A.M. and Sá, L.R.M., 2016. Virulence and antimicrobial resistance of Klebsiella pneumoniae isolated from passerine and psittacine birds. Avian Pathology, 45(2), pp.194-201. |
| 70 | Bennai, K., Tahir, D., Lafri, I., Bendjaballah-Laliam, A., Bitam, I. and Parola, P., 2018. Molecular detection of Leishmania infantum DNA and host blood meal identification in Phlebotomus in a hypoendemic focus of human leishmaniasis in northern Algeria. PLoS Neglected Tropical Diseases, 12(6), p.e0006513. |
| 71 | Evangelista, K.V. and Coburn, J., 2010. Leptospira as an emerging pathogen: a review of its biology, pathogenesis and host immune responses. Future microbiology, 5(9), pp.1413-1425. |
| 72 | Nascimento, A.L.T.O.D., Verjovski-Almeida, S., Van Sluys, M.A., Monteiro-Vitorello, C.B., Camargo, L.E.A., Digiampietri, L.A., Harstkeerl, R.A., Ho, P.L., Marques, M.V., Oliveira, M.C. and Setubal, J.C., 2004. Genome features of Leptospira interrogans serovar Copenhageni. Brazilian journal of medical and biological research, 37(4), pp.459-477. |
| 73 | Dhama, K., Karthik, K., Tiwari, R., Shabbir, M.Z., Barbuddhe, S., Malik, S.V.S. and Singh, R.K., 2015. Listeriosis in animals, its public health significance (food-borne zoonosis) and advances in diagnosis and control: a comprehensive review. Veterinary Quarterly, 35(4), pp.211-235. |
| 74 | Magden, E.R., Mansfield, K.G., Simmons, J.H. and Abee, C.R., 2015. Nonhuman primates. In Laboratory animal medicine (pp. 771-930). Academic Press. |
| 75 | Hallam, S.J., Koma, T., Maruyama, J. and Paessler, S., 2018. Review of mammarenavirus biology and replication. Frontiers in Microbiology, 9, p.1751. |
| 76 | Lafferty, K.D., Hathaway, S.A., Wegmann, A.S., Shipley, F.S., Backlin, A.R., Helm, J. and Fisher, R.N., 2010. Stomach nematodes (Mastophorus muris) in rats (Rattus rattus) are associated with coconut (Cocos nucifera) habitat at Palmyra Atoll. Journal of Parasitology, 96(1), pp.16-20. |
| 77 | Zaleśny, G. and Hildebrand, J., 2012. Molecular identification of Mesocestoides spp. from intermediate hosts (rodents) in central Europe (Poland). Parasitology research, 110(2), pp.1055-1061. |
| 78 | Balasingam, E., 1963. Experimental infection of dogs and cats with Molineus barbatus Chandler, 1942, with a discussion on the distribution of Molineus spp. Canadian Journal of Zoology, 41(4), pp.599-602. |
| 79 | Berenji, F., Fata, A. and Hosseininejad, Z., 2007. A case of Moniliformis moniliformis (Acanthocephala) infection in Iran. The Korean journal of parasitology, 45(2), p.145. |
| 80 | Stevenson, K., Alvarez, J., Bakker, D., Biet, F., De Juan, L., Denham, S., Dimareli, Z., Dohmann, K., Gerlach, G.F., Heron, I. and Kopecna, M., 2009. Occurrence of Mycobacterium avium subspecies paratuberculosis across host species and European countries with evidence for transmission between wildlife and domestic ruminants. BMC microbiology, 9(1), p.212. |
| 81 | Bryant, V., 1973. The life cycle of Nematospiroides dubius, Baylis, 1926 (Nematoda: Heligmosomidae). Journal of helminthology, 47(3), pp.263-268. |
| 82 | Ogilvie, B.M. and Jones, V.E., 1971. Nippostrongylus brasiliensis: a review of immunity and the host/parasite relationship in the rat. Experimental parasitology, 29(1), pp.138-177. |
| 83 | Luce-Fedrow, A., Lehman, M.L., Kelly, D.J., Mullins, K., Maina, A.N., Stewart, R.L., Ge, H., John, H.S., Jiang, J. and Richards, A.L., 2018. A review of scrub typhus (Orientia tsutsugamushi and related organisms): then, now, and tomorrow. Tropical medicine and infectious disease, 3(1), p.8. |
| 84 | Kim, D.C., 1984. Paragonimus westermani: life cycle, intermediate hosts, transmission to man and geographical distribution in Korea. Arzneimittel-Forschung, 34(9B), pp.1180-1183. |
| 85 | Church, M.L., Barrett, P.M., Swenson, J., Kinsella, J.M. and Tkach, V.V., 2013. Outbreak of Philophthalmus gralli in four greater rheas (Rhea americana). Veterinary ophthalmology, 16(1), pp.65-72. |
| 86 | Smales, L.R., Harris, P.D. and Behnke, J.M., 2009. A redescription of Protospirura muricola Gedoelst, 1916 (Nematoda: Spiruridae), a parasite of murid rodents. Systematic Parasitology, 72(1), p.15. |
| 87 | El-Shehabi, F.S., Abdel-Hafez, S.K. and Kamhawi, S.A., 1999. Prevalence of intestinal helminths of dogs and foxes from Jordan. Parasitology research, 85(11), pp.928-934. |
| 88 | Müller, T., Hahn, E.C., Tottewitz, F., Kramer, M., Klupp, B.G., Mettenleiter, T.C. and Freuling, C., 2011. Pseudorabies virus in wild swine: a global perspective. Archives of virology, 156(10), p.1691. |
| 89 | Dewi, K., 2010. THE TAXONOMIC STATUS OF PTERYGODERMATITES SPP. AND THE SCANNING ELECTRONMICROSCOPY STUDY OF PTERYGODERMATITES WHARTONI (TUBANGUI, 1931)(NEMATODA: RICTULARIIDAE) FROM INDONESIAN MURIDS. Zoo Indonesia, 19(1). |
| 90 | Scioscia, N.P., Beldomenico, P.M. and Denegri, G.M., 2016. New host and distribution expansion for Pterygodermatites (Multipectines) affinis. Helminthologia, 53(1), pp.81-86. |
| 91 | Fooks, A., Cliquet, F., Finke, S. et al. 2017. Rabies. Nat Rev Dis Primers 3, 17091 . |
| 92 | Mokrani, N., Parola, P., Tebbal, S., Dalichaouche, M., Aouati, A. and Raoult, D., 2008. Rickettsia aeschlimannii infection, Algeria. Emerging infectious diseases, 14(11), p.1814. |
| 93 | Socolovschi, C., Gaudart, J., Bitam, I., Huynh, T.P., Raoult, D. and Parola, P., 2012. Why are there so few Rickettsia conorii conorii-infected Rhipicephalus sanguineus ticks in the wild?. PLoS Negl Trop Dis, 6(6), p.e1697. |
| 94 | Inokuma, H., Matsuda, H., Sakamoto, L., Tagawa, M. and Matsumoto, K., 2011. Evaluation of Rickettsia japonica pathogenesis and reservoir potential in dogs by experimental inoculation and epidemiologic survey. Clinical and Vaccine Immunology, 18(1), pp.161-166. |
| 95 | Peniche-Lara, G., Dzul-Rosado, K., Perez-Osorio, C. and Zavala-Castro, J., 2015. Rickettsia typhi in rodents and R. felis in fleas in Yucatán as a possible causal agent of undefined febrile cases. Revista do Instituto de Medicina Tropical de São Paulo, 57(2), pp.129-132. |
| 96 | Bäumler, A.J., Tsolis, R.M., Ficht, T.A. and Adams, L.G., 1998. Evolution of Host Adaptation inSalmonella enterica. Infection and immunity, 66(10), pp.4579-4587. |
| 97 | Pulford, C.V., Perez-Sepulveda, B.M., Rodwell, E.V., Weill, F.X., Baker, K.S. and Hinton, J.C., 2019. Salmonella enterica serovar Panama, an understudied serovar responsible for extraintestinal salmonellosis worldwide. Infection and immunity, 87(9), pp.e00273-19. |
| 98 | Giannella, R.A., 1996. Chapter 21: Salmonella. Medical microbiology, 4. |
| 99 | Johnson, P.T., Lund, P.J., Hartson, R.B. and Yoshino, T.P., 2009. Community diversity reduces Schistosoma mansoni transmission, host pathology and human infection risk. Proceedings of the Royal Society B: Biological Sciences, 276(1662), pp.1657-1663. |
| 102 | Diaz, A., Coffey, L.L., Burkett-Cadena, N. and Day, J.F., 2018. Reemergence of St. Louis encephalitis virus in the Americas. Emerging infectious diseases, 24(12), p.2150. |
| 103 | Hughes, J.M., Wilson, M.E., Wertheim, H.F., Nghia, H.D.T., Taylor, W. and Schultsz, C., 2009. Streptococcus suis: an emerging human pathogen. Clinical Infectious Diseases, 48(5), pp.617-625. |
| 104 | Staats, J.J., Feder, I., Okwumabua, O. and Chengappa, M.M., 1997. Streptococcus suis: past and present. Veterinary research communications, 21(6), pp.381-407. |
| 105 | SATO, H., SUZUKI, K., UNI, S. and KAMIYA, H., 2005. Recovery of the everted cystacanth of seven acanthocephalan species of birds from feral raccoons (Procyon lotor) in Japan. Journal of veterinary medical science, 67(12), pp.1203-1206. |
| 106 | Sato, H., Suzuki, K., Osanai, A., Kamiya, H. and Furuoka, H., 2006. Identification and characterization of the threadworm, Strongyloides procyonis, from feral raccoons (Procyon lotor) in Japan. Journal of Parasitology, 92(1), pp.63-68. |
| 107 | Fisher, M.C. and Viney, M.E., 1998. The population genetic structure of the facultatively sexual parasitic nematode Strongyloides ratti in wild rats. Proceedings of the Royal Society of London. Series B: Biological Sciences, 265(1397), pp.703-709. |
| 108 | Stahl, W., 1961. Syphacia muris, the rat pinworm. Science, 133(3452), pp.576-577. |
| 109 | Sato, Y., Ooi, H.K., Nonaka, N., Oku, Y. and Kamiya, M., 1995. Antibody production in Syphacia obvelata infected mice. The Journal of parasitology, pp.559-562. |
| 111 | Ntoukas, V., Tappe, D., Pfütze, D., Simon, M. and Holzmann, T., 2013. Cerebellar cysticercosis caused by larval Taenia crassiceps tapeworm in immunocompetent woman, Germany. Emerging infectious diseases, 19(12), p.2008. |
| 112 | Haby, M.M., Sosa Leon, L.A., Luciañez, A., Nicholls, R.S., Reveiz, L. and Donadeu, M., 2020. Systematic review of the effectiveness of selected drugs for preventive chemotherapy for Taenia solium taeniasis. PLoS neglected tropical diseases, 14(1), p.e0007873. |
| 113 | Pritchett-Corning, K.R. and Clifford, C.B., 2012. Parasitic Infections of Laboratory Mice. The Laboratory Mouse, ed. HJ Hedrich, pp.503-518. |
| 114 | Olds, C.L., Mason, K.L. and Scoles, G.A., 2018. Rhipicephalus appendiculatus ticks transmit Theileria parva from persistently infected cattle in the absence of detectable parasitemia: implications for East Coast fever epidemiology. Parasites & vectors, 11(1), p.126. |
| 115 | Rodan, I. and Sparkes, A.H., 2012. Preventive health care for cats. The Cat, p.151. |
| 116 | Despommier, D., 2003. Toxocariasis: clinical aspects, epidemiology, medical ecology, and molecular aspects. Clinical microbiology reviews, 16(2), pp.265-272. |
| 117 | Aguirre, A.A., Longcore, T., Barbieri, M., Dabritz, H., Hill, D., Klein, P.N., Lepczyk, C., Lilly, E.L., McLeod, R., Milcarsky, J. and Murphy, C.E., 2019. The one health approach to toxoplasmosis: epidemiology, control, and prevention strategies. EcoHealth, 16(2), pp.378-390. |
| 118 | Gottstein, B., Pozio, E. and Nöckler, K., 2009. Epidemiology, diagnosis, treatment, and control of trichinellosis. Clinical microbiology reviews, 22(1), pp.127-145. |
| 119 | Otto, G.M., Franklin, C.L. and Clifford, C.B., 2015. Biology and diseases of rats. In Laboratory animal medicine (pp. 151-207). Academic Press. |
| 120 | Bundy, D.A., Appleby, L.J. and Brooker, S.J., 2020. Nematodes Limited to the Intestinal Tract (Enterobius vermicularis, Trichuris trichiura, Capillaria philippinensis, and Trichostrongylus spp.). In Hunter's Tropical Medicine and Emerging Infectious Diseases (pp. 834-839). Content Repository Only!. |
| 122 | Crisi, P.E., Di Cesare, A. and Boari, A., 2018. Feline troglostrongylosis: current epizootiology, clinical features, and therapeutic options. Frontiers in Veterinary Science, 5, p.126. |
| 123 | Noireau, F., Diosque, P. and Jansen, A.M., 2009. Trypanosoma cruzi: adaptation to its vectors and its hosts. Veterinary research, 40(2), pp.1-23. |
| 124 | Lee C.M., Armstrong E. 2004. Rodent Trypanosomiasis: A Comparison Between Trypanosoma Lewisi and Trypanosoma Musculi. In: Encyclopedia of Entomology. Springer, Dordrecht. |
| 125 | Reinemeyer, C.R., 2016. Formulations and Clinical Uses of Pyrimidine Compounds in Domestic Animals. In Pyrantel Parasiticide Therapy in Humans and Domestic Animals (pp. 67-107). Academic Press. |
| 126 | Clé, M., Beck, C., Salinas, S., Lecollinet, S., Gutierrez, S., Van de Perre, P., Baldet, T., Foulongne, V. and Simonin, Y., 2019. Usutu virus: A new threat?. Epidemiology & Infection, 147. |
| 127 | Petzold, J., van den Brand, J.M., Nobach, D., Hoffmann, B., Hoffmann, D., Fast, C., Reusken, C.B., van Run, P.R., Schlottau, K., Beer, M. and Herden, C., 2019. Distribution of zoonotic variegated squirrel bornavirus 1 in naturally infected variegated and Prevost’s squirrels. Scientific reports, 9(1), pp.1-10. |
| 128 | Londono-Renteria, B. and Colpitts, T.M., 2016. A brief review of West Nile virus biology. In West Nile Virus (pp. 1-13). Humana Press, New York, NY. |
| 129 | Bergren, N.A., Haller, S., Rossi, S.L., Seymour, R.L., Huang, J., Miller, A.L., Bowen, R.A., Hartman, D.A., Brault, A.C. and Weaver, S.C., 2020. “Submergence” of Western equine encephalitis virus: Evidence of positive selection argues against genetic drift and fitness reductions. PLoS pathogens, 16(2), p.e1008102. |
| 130 | Tompkins, D.M., Paterson, R., Massey, B. and Gleeson, D.M., 2010. Whataroa virus four decades on: emerging, persisting, or fading out?. Journal of the Royal Society of New Zealand, 40(1), pp.1-9. |
| 131 | Gage, K.L. and Kosoy, M.Y., 2005. Natural history of plague: perspectives from more than a century of research. Annu. Rev. Entomol., 50, pp.505-528. |
| 132 | Percival, S.L. and Williams, D.W., 2014. Yersinia. In Microbiology of Waterborne Diseases (pp. 249-259). Academic Press. |
| 133 | Vorou, R., 2016. Zika virus, vectors, reservoirs, amplifying hosts, and their potential to spread worldwide: what we know and what we should investigate urgently. International Journal of Infectious Diseases, 48, pp.85-90. |
