# Supplementary material for: The role of invasive alien species in the emergence and spread of zoonoses
Source: Biol Invasions. 2022 Dec 20;25(4):1249–64. doi: 10.1007/s10530-022-02978-1 (PMC9763809; doi:10.1007/s10530-022-02978-1)
Supplement: Supplementary file 3 — Supplementary file3 (DOCX 56 kb) [file 10530_2022_2978_MOESM3_ESM.docx]

**Supplementary Information 3**

The clearest evidence of actual impacts on transmission were for the well-studied impacts of the Norwegian rat, *Rattus norvegicus*, and the black rat, *Rattus rattus*, on the spread and maintenance of serious human pathogens such as bacteria from the genera *Leptospira*, *Bartonella* and *Yersinia* following their widespread human-mediated introduction to new areas, especially islands, through shipping during the 16^th^ and 17^th^ centuries. The evidence for the actual impacts on transmission was based on global biogeographical studies of pathogens detected from rats and humans, facilitated by advances in molecular methods (Wells *et al.* 2015; Puckett *et al.* 2016; Kosoy & Bai 2019) alongside empirical studies on host-vector-pathogen interactions (Moseley *et al.* 2018). Other significant actual impacts come from more recent invaders to Europe, such as *Aedes* mosquito vectors including *Ae. albopictus* providing the conditions for autochthonous outbreaks of arboviruses such as Dengue and Chikungunya in Europe within a decade of initial invasion and establishment (Kraemer et al 2019). Additionally there is evidence that the raccoon dog, *Nyctereutes procyonoides,* has modulated rabies transmission in Eastern Europe (Laurimaa *et al.* 2015). Another clear example of where an IAS has already altered the distribution of human disease outbreaks, was the introduction of the brown ear tick *Rhipicephalus appendiculatus* to the Comoros Islands (Indian Ocean) through cattle imports that led to epidemics of East Coast Fever (*Theileria parva*) in 2004 (Yssouf *et al.* 2011).

*Mammals*

Of the 57 mammalian host-pathogen interactions assessed there were only 14 documented examples of the IAS and native host being similar with respect to pathogen prevalence and 16 cases in which the pathogen was less prevalent in the IAS host versus the native host. In most cases the pathogen was more prevalent in the IAS host than the native host (n=33). However, there was considerable variability even within a given host. As an example, of the six papers comparing 21 interactions between raccoon dogs, *N. procyonoides*, and native hosts (in all but one case red fox, *V. vulpes*), for 10 of the interactions the prevalence was lower in raccoon dogs than the native host, for five there was an increase and a further four the prevalence was the same while for two of the studies the results were mixed. Although interestingly the prevalence of *Leptospira* species was consistently higher in all cases of IAS host-pathogen interaction compared to native host-pathogen interactions, noting the IAS hosts included house mouse, *M. musculus*, Norway rat, *Rattus norvegicus*, black rat, *R. rattus*, and Indian musk shrew, *Suncus murinus*.

*Birds*

The role of alien bird species as hosts for zoonotic diseases was represented in relatively few studies from our review. Indeed only 26 interactions between invasive alien bird species and zoonotic pathogen systems were extracted from the papers we assessed. The majority were from prior to 2000 and few studies documented actual (as opposed to potential) impacts on zoonotic pathogen spillover, which is perhaps the most significant role that invasive alien birds could play in pathogen dynamics. Alien birds are known to play an important role as maintenance hosts for mosquito-borne arboviruses with spillover to humans (Pfeffer and Dobler, 2010). This is particularly relevant in the United States, where several alphaviruses (Eastern equine encephalitis virus, Western equine encephalomyelitis virus, Togaviridae: Alphavirus) and flaviviruses (West Nile virus, Saint Louise encephalitis virus, Flaviviridae: Flavivirus) are relatively widespread and cause febrile illnesses and encephalitis in people (occasionally resulting in neurological problems and death). These viruses tend to be maintained by cycles between infected birds and mosquitoes, with transmission to humans requiring “bridge vector” mosquitoes that bite mammals as well as birds. Many of the introduced bird species sampled in peridomestic settings in the United State in 2000 were found to have a very low prevalences (<5%) of Saint Louise encephailitis, Japanese encephalitis and Western equine encephalomyelitis viruses (Gruwell *et al.* 2000). By exception, the introduced sparrow, *Passer domesticus*, and the European starling, *Sturnus vulgaris*, had levels of viremia to Eastern equine encephalomyelitis that were as or more intense and lasted longer than those of other native bird and mammal species (Komar *et al.* 1999; Arrigo *et al.* 2010), making them likely maintenance hosts.

Of significance for public health is the finding that some birds that have been introduced to Europe can harbour influenza A viruses that have been implicated in human outbreaks, including the Mandarin duck, *Aix galericulata*, in its native range (H5N1 strains) (Lee *et al.* 2011) and the ring-necked parakeet, *Psittacula krameri*, in its invaded range (H9N2 strains) (Mase *et al.* 2001). Avian influenza viruses represent a major concern for animal and human health worldwide (Naguib *et al.* 2019). Indeed the emergence of avian influenza virus H5 (highly pathogenic) and H7N9 (low pathogenic) have led to socioeconomic losses in the poultry industry and fatal human infections. It is important to recognise the role of complex community assemblages, comprising multiple bird species, and their interactions within the wider ecosystem when considering the maintenance of influenza A viruses (Caron et al. 2017).

Several introduced birds, including Canada geese, *Branta canadensis*, and the ring-necked parakeet, *Psittacula krameri*, were implicated as hosts of *Crytosporidium* and *Giardia* in farmland settings in New Zealand (Chilvers *et al.* 1998) and the UK (Feare *et al.* 1999; Dickx *et al.* 2013), with human cases of Giardia being relatively common in New Zealand. Psittacosis is an acute respiratory disease (ranging from mild illness to pneumonia) caused by infection with the bacterium, *Chlamydia psittaci*, following inhalation of airborne particles secreted by bird hosts. Some bird IAS have been found to be key hosts of this pathogen in urban park or farmed settings in Europe and New Zealand (Dickx *et al.* 2013; Pisanu *et al.* 2018; Gedye *et al.* 2018) but the risk is soley restricted to the small number of people who come into direct contact with birds.

*Mosquitoes*

Previous studies have highlighted successive waves of invasion of vector mosquitoes since the 15^th^ century largely due to worldwide ship transport, most notably *Aedes aegpyti* and the *Culex pipiens* complex, as well as *Ae. albopictus* (Lounibos 2002). An increase in the spread of alien mosquitoes in Europe has been seen since the 1990s, linked to increased global trade and travel (Schaffner, Medlock & Van Bortel 2013), with six invasive *Aedes* mosquito species now established in Europe - *Ae.albopictus, Ae. aegypti, Ae. japonicus, Ae. koreicus, Ae. atropalpus*, and *Ae. triseriatus*. This intensification of trade and travel has not only promoted the expansion of alien mosquito species, but has also increased the rate of imported cases of dengue, chikungunya or Zika viruses in continental Europe, resulting in local outbreaks in several European countries (Martinet *et al.* 2019).

Mosquito species that occupy transportable container habitats, such as water-holding automobile tires, have been especially successful as recent invaders. Propagule pressure, previous success, and adaptations to human modified habitats appear to favor successful invasions by vectors (Lounibos 2002; Wilke, Benelli & Beier 2020). The most significant public health threats from alien mosquitoes arise from their ability to transmit zoonotic arboviruses, particularly flaviviruses, alphaviruses, bunyaviruses and orthabunyaviruses (Schaffner, Medlock & Van Bortel 2013) but they also transmit other important pathogens like filarial worms and protozoan parasites (Schaffner, Medlock & Van Bortel 2013). Since the invasion status of mosquito species is often not an explicit consideration in vector incrimination studies, particularly for long-standing invaders, our search terms captured only a fraction of relevant literature on interactions between invasive mosquitoes and pathogens. Therefore, we also included existing review studies (Lounibos 2002; Schaffner, Medlock & Van Bortel 2013; Martinet *et al.* 2019; Wilke, Benelli & Beier 2020) that have integrated field evidence and laboratory competence and infection studies and largely limit our focus here to mosquito species alien to Europe (Wilke, Benelli & Beier 2020).

*Ae. albopictus* has played the greatest role in facilitating the autochthonous transmission of arboviruses in Europe. This species spread dramatically across Europe, largely through the trade in used tyres, since it arrived in Albania in 1979. *Ae. albopictus* breeds in water containers in urban habitats and is aggressively anthropophilic, preferring and seeking humans as hosts, but also has the widest range of competence for arboviral pathogens amongst native and alien mosquito species in Europe (Martinet *et al.* 2019). Considering vector roles in the recent chikungunya virus outbreaks in 2007 (Italy, France), only *Ae. albopictus* (in France) and *Ae. japonicus* (Germany) have been shown to be competent for transmission and their adult activity seasons coincide with the seasonal peak of imported cases, promoting autochthonous transmission. *Ae. aegypti* and *Ae. albopictus* are the main vectors of chikungunya virus but *Ae. koreicus* has also been shown to be a competent vector under optimal rearing conditions (Ciocchetta *et al.* 2018). Temperature fluctuations reduce the risk of transmission by *Ae. koreicus* and so transmission by this vector species is likely to be limited in Europe. Furthermore, there is evidence that a substitution in the adaptive envelope glycoprotein in the Central African chinkungunya virus strain enhanced transmission by *Ae. albopictus* (Tsetsarkin *et al.* 2007). Sporadic local outbreaks of dengue in France and Croatia in 2010, 2013 and 2015 (causing >1500 cases from 2012-2016) have also been attributed to the establishment of *Ae. albopictus* (Schaffner, Medlock & Van Bortel 2013). *Ae. albopictus* and *Ae. aegypti* are the main vectors of dengue in urban cycles globally (Lounibos 2002; Schaffner, Medlock & Van Bortel 2013) and thus known to be competent, but *Ae. japonicus* populations in Germany have also shown competence to transmit tropical Dengue strains and the adult activity periods of all three of these species overlap with the part of the year when imported cases occur.

In terms of future potential risk of Zika virus transmission in Europe, *Ae. albopictus* is also expected to take the most prominent vector role. Only *Ae. albopictus* (France, Germany) and *Ae. japonicus* (Germany) are competent mosquitoes, the latter only poorly competent, whilst native *Culex* species have not been found competent for Zika virus (Martinet *et al.* 2019). Thus, Martinet and others (Martinet *et al.* 2019) conclude that the three species alien to Europe *Ae. albopictus, Ae. koreicus* and *Ae. japonicus* are the species most likely to play a role in circulation of chikungunya, dengue and Zika virus in Europe^[[1]](#footnote-1)^. By contrast, native *Culex* species would likely play a stronger role in transmission of Usutu and West Nile viruses, because of high competence and their ability to act as bridge vectors between avian and mammalian hosts (Martinet *et al.* 2019).

These six *Aedes* species have been linked to a wide range of other arboviruses in their native ranges as well as elsewhere in their invaded ranges, through field infection rates and laboratory competence studies (Schaffner, Medlock & Van Bortel 2013), though their precise roles in transmission under natural conditions is often still unclear. For a full list of these interactions see (Schaffner *et al.* 2009; Martinet *et al*. 2019). Furthermore, several mosquito species including *Ae. aegypti*, *Cx. pipiens*, *Ae. japonicus*, and *Ae. albopictus*, have been linked to transmission of zoonotic filaroid helminths, *Dirofilaria repens* and *D. immitis*, outside the native range of the mosquito species, either through laboratory competence studies or findings of infective stages of the pathogens in wild-caught adults (Bocková *et al.* 2015). These filarioid helminths use domestic and wild canids as main hosts and mosquitoes as vectors, and only occasionally infect humans, though autochthonous cases of human dirofilariosis are now increasing in Italy, anecdotally linked with *Ae. albopictus*.

Alien mosquitoes, as vectors of pathogens that cause disease in humans, are managed in cooperation with WHO Europe, the European Centre for Disease Prevention and Control, the European Mosquito Control Association and Member States, under a regional framework for surveillance and control of invasive mosquitoes and re-emerging mosquito-borne diseases. Guidelines have also been developed (Schaffner *et al.* 2013). However, a similar framework has not yet been developed for other IAS that are vectors, hosts or ectoparasites.

Overall, it is clear that introduced arthropod species can alter the transmission of zoonotic pathogens quite rapidly (<10 years) following their introduction to new regions. Increasing travel and pathogen introduction, expansion of vector distribution, and both environmental and climatic changes are likely to raise the risk of pathogen transmission in Europe, and indeed globally, by these alien *Aedes* mosquitoes (Benedict *et al.* 2007; Schaffner, Medlock & Van Bortel 2013; Kraemer *et al.* 2019; Montarsi *et al.* 2019; Martinet *et al.* 2019). Several research priorities have been highlighted previously that would improve our ability to predict health outcomes from *Aedes*-parasite interactions (Petrić *et al.* 2014; Wilke, Benelli & Beier 2020). These include understanding how and why alien vector species are becoming locally abundant in urban habitats, understanding their basic behaviour, the developmental and life-history parameters that contribute to vectorial capacity alongside vector competence, and how these are modulated by environmental variability (Petrić *et al.* 2014).

## *Alien arthropods (Crustacea) as hosts for enteric bacterial diseases and food-borne trematodes*

In one study from the UK, an introduced barnacle, *Austrominius (Elminius) modestus*, was found to act as a significant reservoir of coliform enteric bacteria on shell-fish beds, harbouring higher concentrations of the bacteria than other barnacle species or the native mussel, *Mytilus edulis* (Clements *et al.* 2013). Surface-dwelling barnacles, such as *A. modestus*, are commonly associated with shellfish and are recognised as a potential source of contaminants for commercially harvested mussels and so pose a risk to humans when shellfish are ingested. In terms of introduced Crustacea listed as IAS of Union concern, the mitten crab, *Eriocheir sinensis*, and the red swamp crayfish, *Procambarus clarkii*, are intermediate hosts for the lung fluke, *Paragonimus westermani*, in Asia which, if eaten, can cause tuberculosis-like and influenza-like symptoms in humans (Hulme 2014; Zhu *et al.* 2019). This lung fluke is widely distributed in South-East Asia and Japan but only rarely found in Europe.

## *Alien molluscs as intermediate hosts for zoonotic pathogens*

Invasive alien molluscs, especially freshwater species, can act as intermediate hosts for nematode, trematode and Platyhelminthes and can infect humans that eat raw or undercooked molluscs. Our literature search identified 11 interactions between IAS of mollusc and such parasitic worms (Table 6), in North and South America, though it was rare for the introduced species to have been explicitly linked to human cases of disease. *Melanoides tuberculata*, the red-rimmed melania, was found to be an intermediate host for a particularly wide range of such parasitic worm pathogens, being linked to human haplorchiasis cases in Venezuela. This species is native to eastern Africa and the Middle East but has spread throughout the tropics, with sporadic records in Spain, Netherlands and Germany, and established populations in Italy. The giant African land snail, *Achatina fulica*, is implicated as an intermediate host of the rat lung worm, but again human cases have rarely been linked to this species (Zhu *et al.* 2019), and the species is localized (Spain) or under eradication in Europe (Italy). Some species of Planorbid snail that have been recently introduced into Eastern Europe (Romania), *Biomphalaria glabrata* and *B. tenagophila* (Majoros *et al.* 2008), can act as intermediate hosts for *Schistosoma mansoni*. This species of trematode causes schistosomiasis, and the associated intermediate host snails are increasingly imported into Europe by immigrants from endemic areas (Hulme 2014). However, explicit links between these species and schistosomiasis transmission have yet to be made for Europe. The establishment of another tropical *Schistosoma* species, *Schistosoma haematobium*, in southern Europe has been linked to transmission by native snail species in Corsica (Mulero *et al.* 2019).

## *Alien fish species as hosts for food-borne zoonotic pathogens*

Alien fish species can act as an important source of zoonotic pathogens, especially such as trematode, cestode and nematode parasitic worms that are acquired by consuming raw or undercooked fish products (Zhu *et al.* 2019) and can cause wide-ranging symptoms, depending on the pathogen species. For example, the WHO estimates that over 2 million disability-adjusted life years worldwide are lost to food-borne trematode parasites each year, concentrated in Africa, Asia and Latin America. We identified 5 interactions between introduced fish species and fish-borne pathogens (Table 7) while Zhu *et al.* (2019) identify a further 60 interactions for IAS in China, though few studies document actual as opposed to potential impact on human disease cases or outbreaks. Of greatest significance to Europe is the finding that introduced salmon species, *Oncorhynchus keta* and *Oncorhynchus nerka*, used for food, harbour the nematode *Anisakis simplex*, and were the source of several human cases of Anisakidosis, an emerging disease in Europe.

## *Alien amphibians as hosts for zoonotic pathogens*

The search terms we used did not identify some of the potential interactions amongst IAS and pathogens. No references were revealed through the structured search relating to amphibians and zoonotic disease. However, there are a few examples in the literature such as the isolation of *Aeromonas hydrophila* from American bullfrog, *Lithobates catesbeianus* (Mauel, Miller & Frazier 2002).

## *Alien reptile species (non-avian Reptilia) as hosts for zoonotic pathogens*

Many studies examining interactions between turtles and tortoises and zoonotic pathogens have been conducted in captive populations, particularly for bacterial pathogens such as Chlamydia and Salmonella. For free-living IAS populations, three such interactions were identified (Table 9), the most important for Europe being the ability of the red-eared slider, *Trachemys scripta elegans*, to harbour both Chlamydia and Salmonella. A high proportion of captive *Testudo hermanni* and *Testudo marginata* populations tested positive for Chlamydiaceae in Poland, but have not yet been detected in free-living populations. It should be noted that some sources of information were not captured by the structured review and as examples the LIFE Project *Trachemys* includes further information on disease in *T. scripta elegans* (see LIFE09 NAT/ES/000529 Demonstration strategy and techniques for the eradication of invasive freshwater turtles) and there has been an observation of anti-leptospiral agglutinins from *T. scripta elegans* in three urban ponds in Italy (Miranda *et al.* 2020).

## *Alien parasites as causal agents for zoonotic diseases in Europe*

There is evidence that several alien endoparasite species have been introduced to Europe (Supplementary Informastion 2) (Hulme 2014). Of most immediate significance is the introduction of the threadworm, *Strongyloides stercoralis*, into Europe thought to be associated with the increase in movement of people travelling to and from the tropics and subtropics (where this threadworm is considered endemic), and consequent increased in strongyloidiasis (respiratory, skin and/or digestive symptoms, with complications in immune-compromised individuals) case and infection rates in people. Domesticated small ruminants act as reservoir hosts and are thought to be enhancing establishment of this parasite in Europe (Puthiyakunnon *et al.* 2014). Also significant is the raccoon roundworm, *Baylisascaris procyonis* that causes a severe or fatal neural condition called baylisascariasis in infected people. Though only one human case has been reported in Europe to date, transmission of this parasite is now widespread in the invaded range of the raccoon dog in Europe (see Table 12), the public health consequences of which should be monitored carefully, given the severe clinical symptoms in infected people. A number of Platyhelminthes have also been introduced, linked to the trade and consumption of fish, particularly where fish are not frozen during transport, but these have caused low numbers of human cases in Europe to date (Supplementary Information 2).

1. For updated information on Aedes spp. in Europe: <https://www.ecdc.europa.eu/en/disease-vectors/surveillance-and-disease-data/mosquito-maps> [↑](#footnote-ref-1)
